# Deciphering Recent Developments in Photocatalysis of Multimetallic Metal–Organic Frameworks and Their Composite Heterojunctions

**DOI:** 10.1002/cssc.71005

**Published:** 2026-09-24

**Authors:** Youjian Chen, Zahra Davoudi, Seyedeh Zeinab Hashemi, Mohammad Mazraeh, Fahime Bigdeli, Amarajothi Dhakshinamoorthy, Wei Dong, Ali Morsali, Hermenegildo Garcia

**Affiliations:** ^1^ School of Chemistry and Chemical Engineering Nanjing University of Science and Technology Nanjing P. R. China; ^2^ Department of Chemistry Faculty of Basic Sciences Tarbiat Modares University Tehran Iran; ^3^ School of Chemistry Madurai Kamaraj University Madurai Tamil Nadu India; ^4^ Departamento de Quimica Universitat Politecnica de Valencia Valencia Spain; ^5^ Instituto de Tecnología Química Consejo Superior de Investigaciones Científicas‐Universitat Politecnica de Valencia Valencia Spain

**Keywords:** heterojunctions, multimetallic‐organic frameworks, photocatalysis, S‐scheme, semiconductors, Z‐scheme

## Abstract

This review summarizes the use of multimetallic‐organic framework (MMOF) composites as photocatalysts, with an emphasis on the role of their constituent components and their heterojunctions. Metal‐organic frameworks (MOFs), with unique features such as regular and uniform porosity, high specific surface area, designable structure, and the possibility of post‐synthetic modification to introduce new active centers, have high potential in the light absorption and charge transfer process. Some of the disadvantages of MOFs, including low stability, charge recombination, and slow charge transfer, can be improved through multimetallic introduction into structure of MOFs and the combination with other semiconductor materials, thereby achieving high photocatalytic efficiency. This review focuses on summarizing the photocatalytic activity of MMOF composites for the degradative removal of organic pollutants, CO_2_ reduction, H_2_ production, and H_2_ production. The definition of MMOF composites can be categorized into main parts that includes (1) single metallic‐organic frameworks (such as Fe‐MIL‐101) with multiple‐metal partner (such as ZnIn_2_S_4_) composites, (2) multimetallic‐organic frameworks (such as MIL‐53(Fe/Cr)) with single‐metal partner (such as CeO_2_) composites, (3) multimetallic‐organic frameworks with multiple‐metal partner composites, (4) MMOFs with carbon‐based partner (such as graphene oxide (GO)) composites, and (5) multiple partners introduction into MOFs (such as NH_2_‐MIL‐125(Ti)/Ti_3_C_2_/ZnIn_2_S_4_). Eventually, the role of MMOF composite in photocatalysis and their possible mechanistic charge carrier dynamic and heterojunctions such as S‐scheme, Z‐scheme, type I, II and other possible mechanism are appropriately discussed.

## Graphical abstract

This review explores the potential of multimetallic organic framework (MMOF) composites as photocatalysts. It demonstrates the impact of the composite architecture on the photocatalytic performance and existing challenges. The paper highlights heterojunction mechanisms and the role of each partner composites in degradation, CO_2_ reduction, H_2_ production, and other applications.

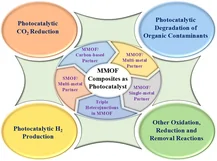

## 1 Introduction

The process of photocatalysis, with the long‐term ambition of converting solar energy into chemical energy, is one of the best ways to drive many chemical reactions, which is of great importance given the energy shift towards green energy sources in the world today [1]. Typically, diverse factors can affect the photocatalysis efficiency, such as light absorption, charge separation, and recombination [2]. To avoid weak charge transportation and to maximize light absorption, the system often requires using various materials, such as semiconductor nanoparticles (NPs), for efficient redox reactions. Poor light absorption is not only pertinent to narrow absorption windows but also refers to low optical extinction coefficients. Also, the plasmonic effect can interact between photons and free carriers in the UV−vis−near‐IR range, which is most commonly observed on metals [3, 4]. So far, many semiconductor photocatalysts have been developed to achieve highly efficient photocatalytic processes [5]. MOFs and their engineering materials with features such as high surface area, tunable functionality, homogeneously distributed pores, active sites, the ability to encapsulate guests, and doping possess not only have potential options for photocatalytic applications [6, 7, 8, 9] but also exhibit unique capability in various applications such as electrocatalysis [10, 11, 12, 13], separation [14, 15], medicine [16], and sensors [17]. MOFs, with these unique properties, provide additional pathways for light‐induced charge separation, which is a key event in photocatalysis [5]. Photoactive MOFs have recently gained attention as a tunable option for heterogeneous photocatalytic reactions, including solar‐to‐chemical energy conversion. Some MOFs, such as MOF‐5 and UiO‐66‐NH_2_ [18, 19], exhibit semiconducting properties. However, pristine MOFs still have some disadvantages, such as low stability, low electrical conductivity, and recombination of h^+^ and e^−^. Therefore, it is necessary to adopt methods to rationally design MOFs and enhance their charge separation efficiency and stability [18, 19]. In addition, the light absorption capacity of most MOFs in the visible range is weak, which prevents the optimal use of solar energy. The fabrication of MOF‐based composites is one of the best ways to overcome these limitations in the photocatalysis of a single MOF material [20]. In MOF composites, which are prepared by combining MOFs with other materials, such as metal NPs and other MOFs, the performance of the composite increases by creating a synergistic effect with the strengths of the constituent components [21]. Large surface areas are a desired parameter in any catalytic process since they favor the availability of active centers to reactants. In composites, MOFs with the largest specific surface area among solids provide a favorable environment for Lewis acid and redox reactions, making it possible to reach optimal catalytic performance [22]. Since the metal center is considered the key active site in many processes and can determine some of the important properties of MOFs, introducing multimetallic active sites into the materials is a suitable method to increase the intrinsic activity of single metallic MOFs without changing their structure [7, 23, 24, 25]. The presence of various metals with different electron affinities can improve catalytic activity and selectivity by adjusting the electron density at the active sites of the native metal [26]. Most MOFs have low charge carrier mobility, resulting in an unsatisfactory quantum efficiency [27]. Researchers have attempted to overcome this weakness by designing a heterogeneous system or by promoting electrical conductivity [28, 29]. Under light irradiation, electrons are generally transferred from the highest occupied molecular orbital (HOMO) of the organic ligands to the lowest unoccupied molecular orbital (LUMO) belonging to the metal ion of the MOF [19]. In this process, the linker ligands of the MOFs act as antennas for energy harvesting, as well as charge transfer from organic linkers to metal nodes (LMCT) [30]. Due to the polarization already existing in the coordinative bonds between metal nodes and linkers, MOFs have unique capabilities as photocatalysts, with potential performance even higher than that of conventional inorganic semiconductor materials. Researchers have attempted to extend the light absorption of MOFs into the visible region by post‐synthetic modifications [31] or rational selection of bridging ligands [32]. Energy band gap and band potentials are important factors in photocatalytic performance, and the characteristics of MOFs, such as morphology, organic ligands, and metal ions, have an effect on their activity [33]. The nature of metal clusters in the secondary building units (SBUs) of MOFs can determine the band structure, and on the other hand, the proximity of the conduction band (CB) and valence band (VB) energies to the redox potential of the radicals is important and leads to an increase in quantum efficiency [34]. In general, diminution of the band gap energy in the visible wavelength range to a value about 2.3 eV has been considered as a reasonable approach to achieve optimal photocatalytic activity. However, variation of the CB and VB energy values can result in poor light adsorption by band gap opening, unwanted electron–hole charge separation caused by inadequate orbital overlap, and slow reaction kinetics due to insufficient overpotential [35]. Therefore, finding solutions for band energy engineering and the control of complex electron–hole interactions is essential to overcome such limitations for effective photocatalytic performance [36].

Besides the band gap energy, the relative positions of the CB and VB, or the LUMO and HOMO levels in MOFs, are also important factors affecting photocatalytic performance. While the band gap determines the range of light that can be absorbed, the band alignment controls the migration of photogenerated electrons and holes across the heterojunction. Therefore, reducing the band gap alone is not sufficient to improve photocatalytic activity; suitable band alignment is also required to promote charge separation while maintaining a sufficient redox potential for photocatalytic reactions. In conventional type‐II heterojunctions, electrons and holes migrate to different semiconductors according to their band positions, resulting in effective charge separation but often at the expense of redox capability. By contrast, Z‐scheme and particularly S‐scheme heterojunctions have attracted increasing attention because they combine efficient charge separation with strong redox capability, making them attractive for photocatalytic reactions such as CO_2_ reduction and H_2_ production. In S‐scheme heterojunctions, the built‐in electric field and band bending promote the selective recombination of charge carriers with weaker redox ability while preserving highly reducing electrons and strongly oxidizing holes. This enables efficient charge separation without sacrificing redox capability and has made S‐scheme heterojunctions an increasingly important design strategy for high‐performance photocatalysts. The exact charge–transfer pathway, however, should be evaluated carefully as mechanistic assignments are often based on indirect experimental evidence and may require complementary characterization and theoretical analysis for reliable confirmation.

MMOFs possess unique electrical properties and often exhibit efficient catalytic activity better than a single‐metal MOF [37]. Doping with metal ions not only creates additional active sites in MMOFs but also improves the light absorption properties of MOFs. Metal‐to‐metal charge transfer (MMCT) between different metal ions enhances the charge transfer and photocatalytic efficiency. In addition, in bimetallic MOFs with a well‐mixed spatial arrangement of metals, charge carrier separation can be improved [38]. In transition‐metal ions with incompletely filled d orbitals, visible light absorption can occur via a d–d transition. Therefore, transition‐metal ions are often applied to the structure of MOFs to increase visible light catalytic performance [39]. Furthermore, the structural flexibility of MMOFs can be controlled by using different metal ions in the SBU [40]. The most common doped metals are noble metals such as Ru, Rh, Ag, Au, and Pt and transition metals such as Ti, Mn, Fe, Co, Ni, Cu, and Zn [41]. The use of transition metals is more common due to their lower cost and better availability compared to noble metals [42].

Organic ligands bonded with various metal ions in MMOFs enhance thermal and chemical stability [43]. MMOFs can be made from two (bimetallic MOFs), three (trimetallic MOFs), and four different metals (tetra‐metallic MOFs) [44, 45]. MMOFs can be synthesized by methods such as direct mixing [44], sacrificial templating [46], and post‐synthesis modification [47]. The synthetic conditions, such as solution pH, solvent, and the ratio of metal ligand and mixed metals, may affect the nucleation process and MOF structure [48].

As mentioned, the rapid recombination of charge carriers in MOFs is the most important obstacle to the desired photocatalytic efficiency. According to research, optimal charge separation and photocatalytic efficiency can be enhanced by creating heterogeneous interfaces between MOFs and other materials. Creating a heterogeneous interface can provide a new pathway for charge transport and effectively enhance charge separation [49, 50]. Therefore, to increase efficiency, the combination of MOFs with conductive materials such as conductive polymers and graphene is considered an effective method [51]. Several different types of heterojunctions such as traditional type‐II heterojunction photocatalysts, Z‐scheme, and S‐scheme photocatalysts exhibit superior photocatalytic activity due to their lower charge recombination rates and enhanced oxidation capabilities [52, 53] and have been used to increase the light absorption range and electron–hole separation [54] Among these, S‐scheme heterojunctions have received increasing attention in recent years because they effectively combine efficient charge separation with strong redox capability, while Z‐scheme heterojunctions remain widely used to explain the behavior of many reported photocatalytic systems [55]. In the Z design, suitable semiconductors for charge transport are selected to lower the CB level and raise the VB level, which leads to the facilitation of the redox reaction. In the Z design, p‐type and n‐type semiconductor junctions are used, which are facilitated by the enhanced electric field [56]. The mode of operation of 2‐S‐scheme heterogeneities is similar to Z‐scheme heterogeneities, except that, in the 2S‐scheme, two photocatalysts must be in direct contact for charge transfer, while Z‐scheme heterojunctions typically require an intermediary to shuttle charges between the two photocatalysts [28, 49].

In recent years, the understanding of charge–transfer mechanisms in semiconductor heterojunctions has continued to evolve. Many systems that were originally described as direct Z‐scheme photocatalysts have increasingly been interpreted as S‐scheme heterojunctions because the S‐scheme model provides a more physically consistent explanation of charge separation based on band bending and the built‐in electric field. Since many of the studies discussed in this review were published before this updated interpretation became widely accepted, we retain the terminology used in the original publications where appropriate while acknowledging the current understanding of these charge–transfer mechanisms [57, 58, 59].

Finding active materials with good compatibility with MOFs could be a turning point in the synthesis of high‐performance composites [22]. The synthesis methods of MOF composites affect their morphology, structure, interactions, and distribution of components, which play an important role in their photocatalytic performance [60, 61, 62]. Various synthetic methods, including solvothermal [63, 64], mechanochemical solid grinding [65], solution impregnation [66], molding, and rotational chemical vapor deposition methods [67], have been used to develop MMOF composites. However, it seems that the solvothermal method has been most frequently used to prepare these composites [29]. There are two main techniques for the synthesis of MMOFs: single‐step synthesis and post‐synthesis modification. Currently, single‐step synthesis is used due to its greater ease [68]. Although the post‐synthesis modification method is relatively complex, it has advantages such as targeted implantation of desired metals and higher controllability in determining metal sites [69]. Li et al. have described in detail various synthesis methods for MMOF composites in a review article [70].

**FIGURE 1:**
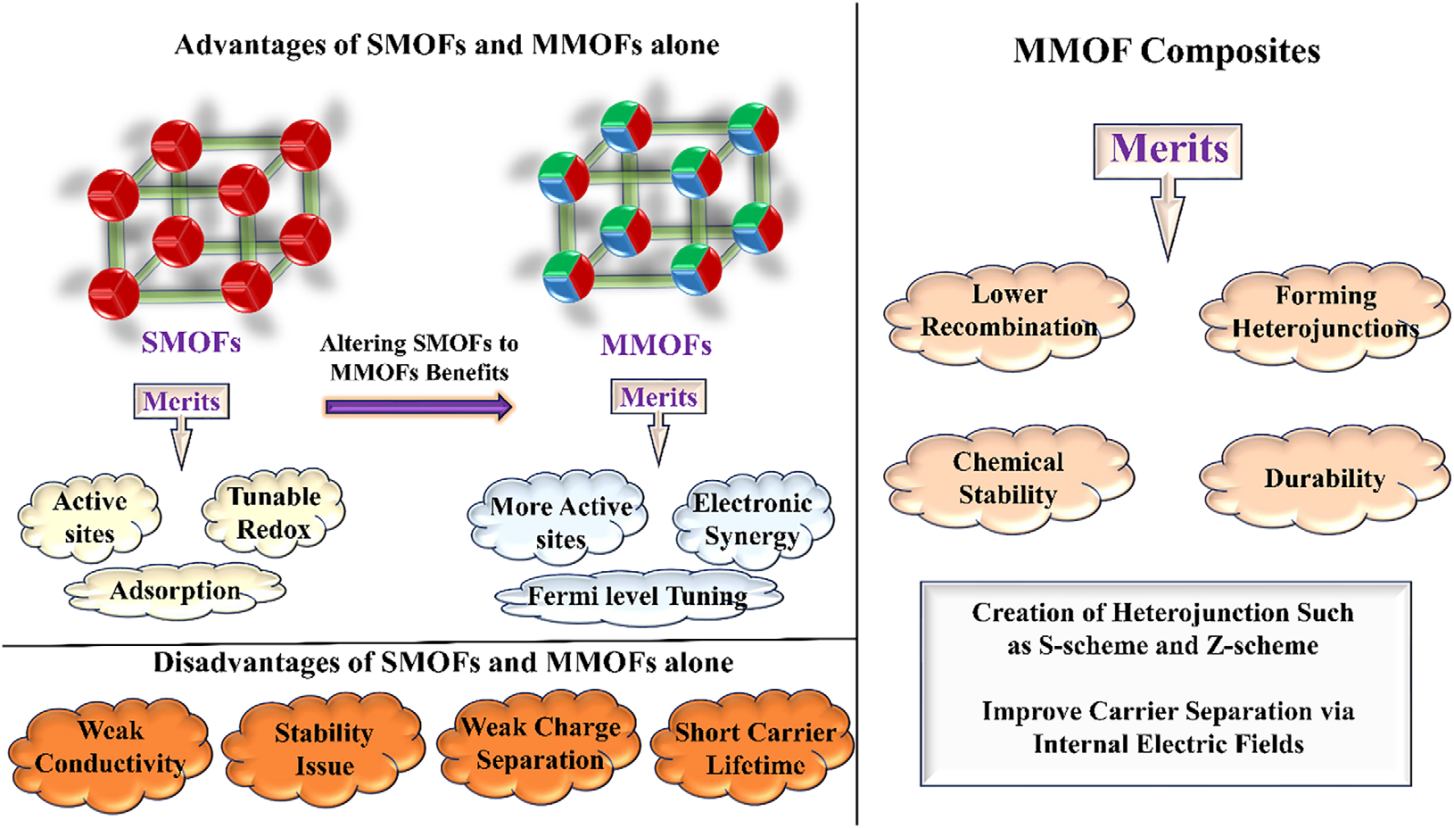
Advantages and disadvantages of SMOFs and MMOFs (left) and merits of using MMOF composites in photocatalytic procedures (right).

The review articles published so far on MMOF composites have focused mostly on synthetic methods [70, 71], and no review article exclusively discusses the field of photocatalytic activity based on the classification of MMOF types and the description of the role of each composite component alongside their heterojunction based on the multimetallic assignments in the photocatalytic performance; therefore, new categorization based on the position of multimetal could be helpful. We presented a classification framework that analyzes the direct impact of the metal composite architecture on the internal electric field. The number of reports shows that recently researchers have moved towards investigating the potential of MMOF composites for photocatalytic activities, so that the number of articles has been growing due to their superior activity compared to the single metallic‐organic frameworks (SMOFs) and MMOFs alone (Figure 1). Because the studies summarized in this review were performed under different experimental conditions, including variations in light source, irradiation intensity, catalyst loading, reactant concentration, and reaction time, direct comparison of photocatalytic performance between different reports should be made with caution. Therefore, the comparisons presented in this review are intended to highlight general trends in material design and photocatalytic behavior rather than establish the superiority of one photocatalyst over another. While some of the lone MMOFs can have some demerits, such as higher band gaps and slow electron–hole charge recombination, the introduction of second partners to the MMOFs can be useful for photocatalytic applications. This review focuses on examining the types of MMOF composites based on the number of metal centers in each of the composite components to discuss their potential in photocatalytic applications. Also, recent advances in the applications of various MMOF composites to perform photocatalytic processes and possible charge carrier dynamics for reactions in diverse applications such as pollutant removal, H_2_ production, carbon dioxide conversion, hydrogen production, and water oxidation are approached. Description of the advantages and disadvantages of MOFs as well as the performance of each composite component in the composite performance is also scrutinized.

## 2 SMOFs/Multimetallic Partner Composites

In recent years, a new generation of semiconductors has emerged including iron oxide‐based multimetal composites, titanium oxide‐based multimetal composites, perovskite composites, layered double hydroxides (LDHs), Bi_2_MO_6_, MVO_4_, and bimetallic sulfides. Despite their improved performance, multimetal composites still face certain challenges, such as limited efficiency under visible light and stability issues. These limitations can be effectively mitigated by constructing heterojunctions or coupling them with other advanced materials, such as MOFs [72].

**FIGURE 2:**
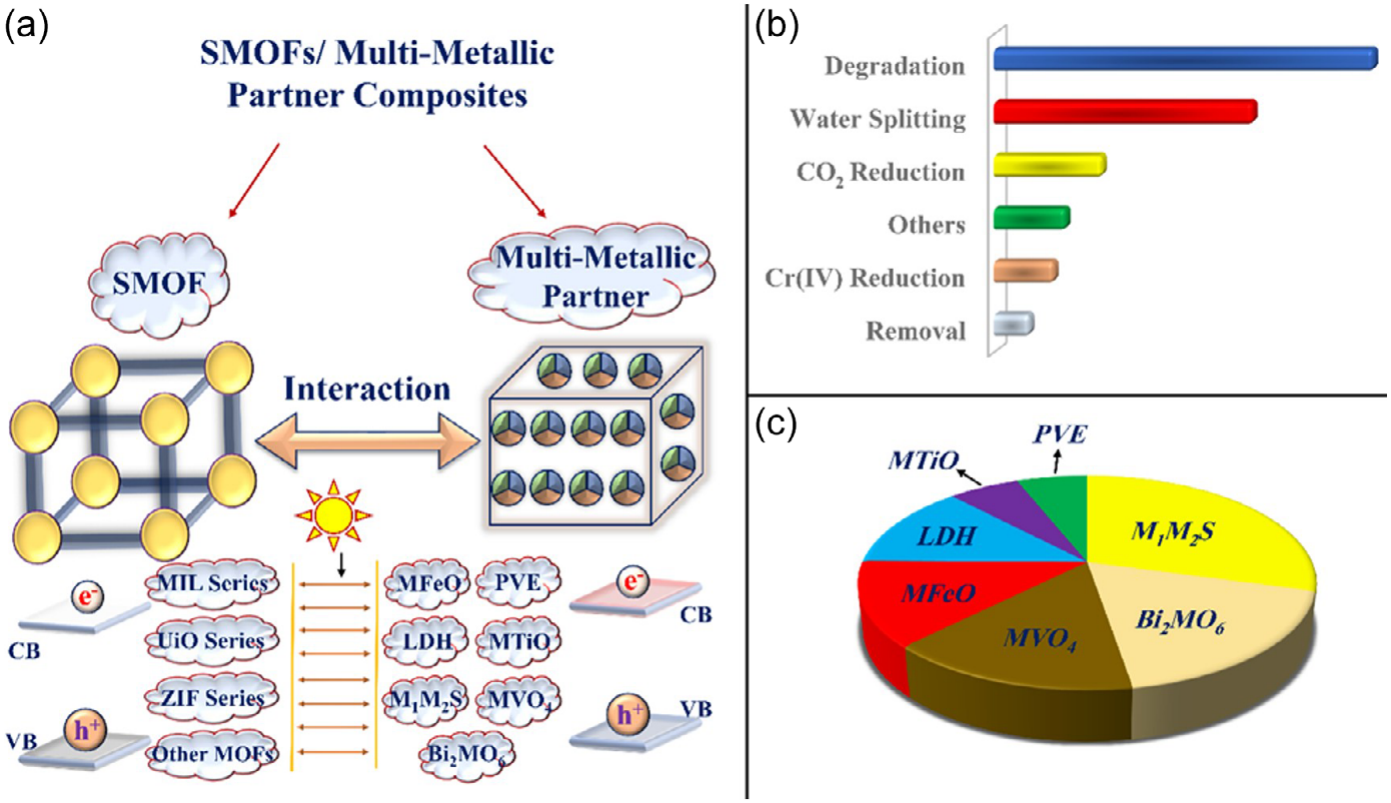
(a) Overall schematic illustration of SMOFs/multimetallic partner composites in this section. (b) Numerical investigation of SMOFs/multimetallic partner composites in diverse photocatalysis procedures. (c) Variety of SMOFs/multimetallic partner composites (M_1_M_2_S, MFeO, LDH, MTiO, and PVE in order denoted as bimetallic sulfides, bimetallic iron‐based oxides, LDH, bimetallic titanium‐based oxides, and perovskites respectively).

According to Figure 2, various classes of multimetallic partner semiconductors and their heterojunction with SMOFs are discussed in this section. Three types have attracted significant attention in MOF‐based composite studies: bimetallic sulfides, MVO_4_‐type vanadates, and Bi_2_MO_6_ Aurivillius‐phase oxides. These materials have attracted significantly more research attention compared to other bimetallic systems, as evidenced by their higher representation in the published literature. This preference can be attributed to several advantages, for instance, low cost and convenient preparation of sulfides [73], close band gap, simple preparation, controllable crystalline aspect, and specific electronic structure of MVO_4_ [74] and also the unique layered structures of Bi_2_MO_6_ [75]. The highly reducing electrons are located in the CB of the SMOF, and the highly oxidizing holes are located in the VB of the multimetallic partner, and the spatial and energy‐efficient separation of the charges is achieved. As a result, reactive species such as radicals and anions are simultaneously generated, driving the photocatalytic performance (Figure 2a). Statistics also reveal that among wide photocatalytic applications of composites, degradation of organic and inorganic pollutants and water splitting have been studied more than the other (Figure 2b). Figure 2c shows multimetallic partner semiconductors, which have obtained considerably more research interest than other bimetallic systems.

### 2.1 MOF/Bi_2_MO_6_ Composites

Bi_2_WO_6_ and Bi_2_MoO_6_ are semiconductors belonging to the Aurivillius oxide family, which have been extensively studied as photocatalysts due to their unique layered structures [76, 77, 78, 79]. Furthermore, these materials exhibit several advantageous properties, including a narrow band gap of approximately 2.7 eV, excellent photochemical stability, visible light absorption, low toxicity, and cost‐effectiveness [80, 81]. Despite these favorable attributes, pure Bi_2_WO_6_ and Bi_2_MoO_6_ face several limitations, such as limited adsorption capacities that impede their practical photocatalytic performance [75, 78]. Another significant challenge is their tendency toward rapid agglomeration. While Bi‐based semiconductors exhibit optimal photocatalytic performance at the nanoscale, such as in the form of nanorods, nanosheets, or nanoflowers, their nanoscale particles tend to aggregate quickly, making their recovery and reuse difficult [82].

Recent studies have shown that coupling Bi_2_WO_6_ and Bi_2_MoO_6_ with other semiconductors, such as MOFs, can effectively address these limitations. This coupling leads to the formation of various heterojunctions, including type‐I [75], S‐scheme [80], Z‐scheme [79, 83, 84, 85], and core–shell [78] heterojunctions. Notably, a lower charge‐transfer resistance is directly correlated with superior photocatalytic performance. Electrochemical analyses have demonstrated that MOF/Bi_2_MO_6_ composites exhibit significantly lower charge‐transfer resistance compared to their components, further underscoring their enhanced efficiency [75]. In the case of heterojunctions in MOF/Bi_2_MO_6_, a range of composites, including NH_2_‐MIL‐101(Fe)/Bi_2_MoO_6_ [80], Co‐MOF/Bi_2_MoO_6_ [79], MIL‐88B(Fe)/Bi_2_WO_6_ [86], Ni‐MOF/Bi_2_WO_6_ [83], and MIL‐101(Fe)/Bi_2_WO_6_ [87], have been reported to exhibit Z‐ or S‐scheme charge–transfer pathways. This interpretation is primarily based on observations such as the simultaneous detection of ^**.**^O_2_^−^ and ^**.**^OH radicals, which cannot be generated under a standard type‐II configuration due to the limited band potentials of Bi_2_MO_6_ [79, 83]. In the proposed Z/S‐scheme systems, photoexcited electrons from the CB of Bi_2_MO_6_ recombine with holes in the VB (or HOMO) of the MOF. This process effectively maintains highly reducing electrons within the MOF's LUMO and strongly oxidizing holes within Bi_2_MO_6_'s VB, ensuring an efficient spatial and energetic separation of charges. As a result, reactive species such as ^**.**^O_2_^−^, ^**.**^OH, and ^1^O_2_ are generated simultaneously, driving photocatalytic processes including CO_2_ reduction, dye degradation, and antibiotic mineralization. The preference for a Z‐ or S‐scheme mechanism has been attributed to the intrinsic band alignment of Bi_2_MO_6_, where its CB lies slightly more positive than the O_2_/^**.**^O_2_^−^ potential (approximately −0.33 V vs. NHE). In a type‐II configuration, electrons would accumulate in this less negative CB, producing an insufficient reduction potential for reactive oxygen species formation. Conversely, the Z‐ or S‐scheme arrangement retains electrons on the more negative CB or LUMO of the MOF, preserving reduction capability while maintaining the oxidative strength of Bi_2_MO_6_. In S‐scheme systems such as MIL‐101(Fe)/Bi_2_WO_6_ [87] and NH_2_‐MIL‐125(Ti)/Bi_2_WO_6_ [88], the difference in work functions between the two semiconductors gives rise to a built‐in interfacial electric field (IEF). This field drives directional charge movement, ensuring efficient carrier separation and improved photocatalytic activity. A few exceptions, including MOF‐5/Bi_2_WO_6_ [75] and Bi‐MOF/Bi_2_WO_6_ [89], exhibit type‐I or type‐II charge transfer. These occur when band offsets and Fermi‐level positions allow direct carrier migration to Bi_2_WO_6_ without significantly reducing redox potential. Although such systems perform well in dye degradation, their weaker redox characteristics limit their effectiveness in more demanding multielectron reactions, such as CO_2_ reduction.

### 2.2 MOF/MVO_4_ Composites

As an n‐type semiconductor, BiVO_4_ has recently emerged as a promising photocatalyst due to its narrow band gap of approximately 2.4 eV, which enables strong visible‐light absorption. Additionally, its well‐positioned VB edge makes it highly suitable for photocatalytic applications as it partially facilitates the formation of S‐scheme heterojunctions when integrated with other materials [72, 90]. Other advantageous properties include nontoxicity, excellent photostability, low cost, and resistance to corrosion, rendering BiVO_4_ an efficient photocatalyst with potential applications in a wide range of photocatalytic reactions [91]. However, despite these benefits, its performance is hindered by limitations such as a lack of active sites, low surface area, slow electron‐transfer rates, and high recombination rates of photogenerated charge carriers [74, 92, 93]. To address these limitations and enhance its photocatalytic properties, composition with MOFs to form heterojunctions can be effective. This strategy not only increases the surface area of BiVO_4_ but also significantly improves the separation and migration of charge carriers, thereby enhancing the photocatalytic efficiency [94]. The formation of Z‐scheme and S‐scheme heterojunctions has been shown to significantly enhance the photocatalytic efficiency by facilitating the effective transfer of photogenerated charge carriers at the interface [95]. This improves the oxidation and reduction capabilities of the valence and conduction bands [96]. However, there is no established method to precisely control electron pathways in photocatalytic reactions to consistently form Z‐ or S‐scheme heterojunctions [95]. Nevertheless, studies have demonstrated that the combination of MOFs and BiVO_4_ holds high potential for forming Z‐scheme heterojunctions due to the differences in their band structures and Fermi energy levels. The disparity in Fermi energy levels between MOFs and BiVO_4_ promotes the formation of built‐in electric fields at the interface, which facilitate efficient charge carrier migration. This enhances electron transfer between MOFs and BiVO_4_, thereby boosting the overall photocatalytic performance [95]. For instance, Zhou and coworkers fabricated a BiVO_4_/CAU‐17 Z‐scheme heterojunction photocatalyst by the in situ growth of BiVO_4_ nanosheets on CAU‐17 using a hydrothermal method. They reported that the construction of a proposed Z‐scheme heterojunction significantly enhanced the photodegradation ability of the composite for RhB and Congo Red (CR) compared to that of pure BiVO_4_ and CAU‐17. They showed that the removal rate of RhB reached 99.3%, which was 1.9 times higher than pristine BiVO_4_ and 2.0 times higher than CAU‐17. Similarly, the removal rate for CR achieved was 97.3%, which was 1.4 times higher than BiVO_4_ and 1.2 times higher than pristine MOF. The composite also achieved synergistic removal of pollutants in the RhB/CR binary system, highlighting its potential for the treatment of dye wastewater [92]. Furthermore, studies have shown that combining BiVO_4_ with MOFs such as MIL‐101(Fe) [97], MIL‐101‐NH_2_ [93], and UiO‐66‐NH_2_ [95] facilitates the formation of Z‐scheme heterojunctions, further enhancing photocatalytic performance. Similarly, coupling BiVO_4_ with various MOFs has been proposed to generate S‐scheme heterojunctions. S‐scheme heterojunctions consist of an oxidation photocatalyst (OP) and a reduction photocatalyst (RP) with a staggered band structure [90]. Selecting appropriate OP and RP materials with narrow band gaps can significantly enhance the photocatalytic efficiency. For the OP, the VB position must be sufficiently positive to enable effective oxidation reactions. The narrow band gap and high chemical stability of BiVO_4_ make it an ideal candidate for the OP in S‐scheme heterojunctions. Conversely, semiconductors with a negatively positioned CB are well suited to function as the RP. Based on the reported band alignment and characterization results, combining MOFs with BiVO_4_ has been proposed to favor S‐scheme charge–transfer pathways [90]. For example, Li et al. proposed that integrating Ni‐MOF‐74 with BiVO_4_ forms an S‐scheme heterojunction based on the reported experimental evidence and attributed the enhanced photocatalytic H_2_ production to this charge–transfer pathway [98]. Similarly, Zhao et al. confirmed that a 2D Zn‐MOF/BiVO_4_ composite also follows the S‐scheme mechanism and shows proper photocatalytic performance in CO_2_ conversion [90].

Furthermore, Ag_3_VO_4_, as an n‐type semiconductor, is another metal‐based vanadate that has been reported to enhance the photocatalytic activity of MOFs when coupled with them [99, 100]. However, they face challenges such as particle agglomeration [101]. Integrating Ag_3_VO_4_ with various MOFs can significantly enhance the photocatalytic efficiency of the resulting composite materials. Despite its potential, the number of studies on Ag_3_VO_4_‐MOF composites remains significantly lower compared to those on BiVO_4_‐based composites. Ag_3_VO_4_ has garnered scientific interest due to its favorable band gap range (2.0–2.5 eV) [102]. For instance, Zhai et al. demonstrated that synthesizing an Ag_3_VO_4_/ZIF‐8 composite significantly enhanced the photocatalytic performance compared to bare ZIF‐8 and Ag_3_VO_4_ in the degradation of RhB. This improvement was attributed to the modification of the MOF's band gap through coupling with Ag_3_VO_4_. Their findings revealed a substantial reduction in the MOF's band gap from 5.1 to 2.11 eV, leading to improved photocatalytic efficiency. They demonstrated that the final composite exhibited 60% degradation of RhB that was approximately 4.2 times higher than pure Ag_3_VO_4_ and 22.8 times higher than bare ZIF‐8 [99]. Furthermore, Zhai et al. reported that coupling MIL‐125(Ti) with Ag_3_VO_4_ decreased its band gap from 3.6 to 2.2 eV, thereby enhancing its photocatalytic performance in the degradation of RhB. They showed that the MIL‐125(Ti)/Ag_3_VO_4_ composite exhibited photocatalytic performance 3.7 times greater than that of pure Ag_3_VO_4_ and 13.1 times higher than that of the pristine MOF [101].

Furthermore, regarding mechanistic routes in MOF/MVO_4_, vanadates like BiVO_4_ and Ag_3_VO_4_ have CB edges located near or slightly more positive than the O_2_/^**.**^O_2_^−^ redox potential (≈−0.33 V vs. NHE) [102, 103]. Consequently, in a typical type‐II arrangement, electrons would accumulate in the vanadate CB, where their reducing ability is insufficient to form reactive oxygen species such as superoxide (^**.**^O_2_^−^). However, experimental evidence, including radical trapping studies, in situ XPS analysis, Mott–Schottky measurements, and band‐bending calculations, indicates that these systems simultaneously exhibit strong oxidative and reductive activities. The detection of both ^**.**^O_2_^−^ and ^**.**^OH radicals, as well as direct hole oxidation, contradicts the behavior expected from type‐II transfer, supporting instead the operation of Z‐ or S‐scheme pathways that preserve high redox power on both components [90, 93, 97]. Configurations demonstrated in BiVO_4_/CAU‐17, ZIF‐67/Ag_3_VO_4_, UiO‐66‐NH_2_/BiVO_4_, and Zn‐MOF/BiVO_4_ exhibited that photoexcited electrons from the vanadate CB recombine with holes from the MOF VB (or HOMO) at the interface, a process facilitated by built‐in IEF and Fermi‐level equilibration. This recombination removes the low‐energy carriers while retaining strongly reducing electrons within the MOF's LUMO or metal nodes and strongly oxidizing holes within the vanadate's VB [90, 92, 95, 102]. Such spatial and energetic separation allows the generation of reactive species (^**.**^O_2_^−^, ^**.**^OH, and h^+^), which drive dye mineralization, antibiotic degradation, organic pollutant decomposition, nitrate reduction, and CO_2_ conversion with high efficiency.

### 2.3 MOF/Bimetallic Sulfide Composites

**FIGURE 3:**
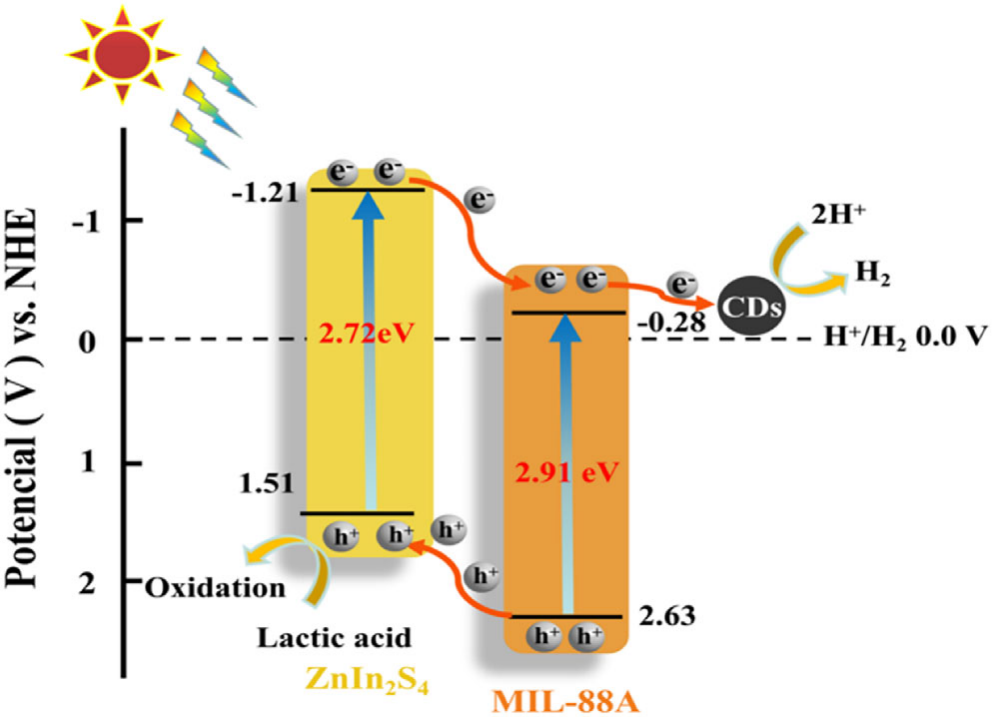
Proposed mechanism of H_2_ production in CDs/ZnIn_2_S_4_@MIL‐88A composites. Reproduced with permission [121]. Copyright 2023, American Chemical Society.

Materials such as MnCdS, ZnCdS, ZnIn_2_S_4_, and CdIn_2_S_4_ exhibit controlled band gaps, tunable electronic properties, cost‐effectiveness, and excellent light absorption, making them highly efficient photocatalysts [104]. However, certain limitations such as low visible light utilization, limited carrier separation efficiency, poor dispersion of active sites, and a tendency for rapid agglomeration exist [105, 106]. MOFs, known for their semiconductor‐like behavior, high surface area, and porosity, present an excellent candidate to overcome these challenges [107]. By forming heterojunctions with bimetallic sulfide solid solutions, the synergistic effects between these materials can effectively suppress the agglomeration, enhance the dispersion of active sites, and optimize the MOF's band gap [105]. Moreover, the formation of chemical interfacial bonds between MOFs and bimetallic sulfides significantly enhances the separation efficiency of photoexcited electrons and holes [106]. This integration also improves the stability of bimetallic sulfides in aqueous environments [108]. As a result, the composite materials demonstrate significantly improved photocatalytic performance, particularly in H_2_ production. MCdS (M = Mn, Zn) semiconductors have been widely reported to couple with various MOFs and are primarily utilized as photocatalysts for H_2_ production. For instance, Li and coworkers fabricated a novel 0D/2D CdZnS/Ni‐MOF‐74 composite via hydrothermal and physical mixing methods. This composite demonstrated exceptional photocatalytic H_2_ production performance, achieving 1713.2 μmol within 5 h, approximately 10 times higher than that of bare CdZnS (170.9 μmol). The enhanced performance was attributed to the composite's unique spatial structure and the formation of a direct Z‐scheme heterojunction between CdZnS and Ni‐MOF‐74, which facilitated efficient charge separation and transfer [109]. Beyond hydrogen production, these composites have been employed in environmental purification applications, including Cr(VI) reduction [106] and dye degradation [110, 111]. Furthermore, ZnIn_2_S_4_ has garnered significant attention as a photocatalyst due to its multilayered structure, optimal band gap, high absorption efficiency, and robust physicochemical stability [108, 112, 113, 114]. Additionally, the suitable alignment of its conduction (−1.06 V_RHE_) and valence band (1.24 V_RHE_), coupled with its sufficiently negative potential for proton reduction in water, makes ZnIn_2_S_4_ an excellent candidate for photocatalytic H_2_ production [115, 116]. Additionally, the porous structure of MOFs can serve as ordered nanoreactors, enabling ZnIn_2_S_4_ to be incorporated within their channels, thereby enhancing the photocatalytic efficiency [117, 118]. The layered crystal structure of ZnIn_2_S_4_ also facilitates intimate interfacial contact with MOFs, increasing the contact surface area and promoting material exchange and electron transport [117, 119, 120]. Furthermore, this integration improves the composite's stability in aqueous environments [119]. These types of composites are widely employed as photocatalysts, particularly in the field of H_2_ production. For instance, Fan et al. prepared CDs/ZnIn_2_S_4_@MIL‐88A (CDs: carbon dots) photocatalysts through the in situ loading of CDs and ZnIn_2_S_4_ nanosheets onto MIL‐88A(Fe) to form a ternary heterojunction. The composite exhibited significantly enhanced photocatalytic hydrogen production performance (1259.63 μmol g^−1^ h^−1^), whereas pristine MIL‐88A(Fe) showed no photocatalytic performance; in comparison, the H_2_ production rate of ZnIn_2_S_4_ under visible light irradiation was 821.17 μmol g^−1^ h^−1^. The improved performance was attributed to the efficient separation and migration of photoinduced charge carriers, facilitated by the close interfacial contact, synergistic effects, and an optimized band structure. As shown in Figure 3, due to the close contact and internal electric field, the electrons of CB in ZnIn_2_S_4_ flow to the LUMO of MIL‐88A(Fe), and the holes on the HOMO of MIL‐88A(Fe) transfer to the VB of ZnIn_2_S_4_. Due to the electron sink effect of CDs, the electrons excited by light irradiation on MIL‐88A(Fe) are mostly transferred to CDs to produce hydrogen [121]. Representative composites such as Cd_0.5_Zn_0.5_S/Ni‐MOF‐74 [109], CdLa_2_S_4_/MIL‐88A(Fe) [122], Cu‐MOF/Cd_0.5_Zn_0.5_S [123], and ZnIn_2_S_4_@PCN‐224 (PCN denoted as porous coordination network) [124] exhibit strong experimental confirmation, via ESR analysis, PL quenching, and Mott–Schottky measurements, that their superior hydrogen production is best explained by a Z‐ or S‐scheme charge transfer model. Under visible‐light illumination, both the MOF and the bimetallic sulfide generate electron–hole pairs. The photoinduced electrons in the MOF's CB (or LUMO) recombine with holes in the sulfide's VB (or HOMO) at the interface. This selective interfacial recombination leaves highly reductive electrons on the sulfide CB and strongly oxidative holes on the MOF VB, thereby preserving strong redox potentials on both sides of the junction. This configuration is especially effective because the CBs of bimetallic sulfides, such as CdIn_2_S_4_ (≈–0.9 to –1.0 V vs. NHE), are sufficiently negative to drive H^+^ reduction to H_2_, while the VBs of Fe‐, Ti‐, or Cu‐based MOFs are positive enough to support the oxidation of sacrificial reagents or water [109, 119, 122]. Thus, the Z‐ and S‐scheme pathways together enable robust redox activity, efficient charge separation, and long‐term stability. The S‐scheme heterojunction, particularly seen in Cu‐MOF/Cd_0.5_Zn_0.5_S [123] and UiO‐66‐NH_2_/CdIn_2_S_4_ [107], adds another layer of efficiency through the creation of IEF arising from Fermi‐level differences between the n‐type and p‐type components. This built‐in potential establishes a natural driving force for charge migration, with electrons concentrating on the sulfide CB (reduction site) and holes retained on the MOF VB (oxidation site) [107, 123]. By refining the classical Z‐scheme, the S‐scheme structure suppresses unwanted charge recombination and facilitates self‐driven carrier transfer without external bias, resulting in higher H_2_ production rates and enhanced photostability during extended irradiation. Conversely, type‐II heterojunctions, as in Cu‐MOF/ZnIn_2_S_4_ [125] and UiO‐66‐NH_2_/ZnIn_2_S_4_ [126], follow a distinct electron flow: electrons move from the sulfide CB to the MOF's LUMO while holes migrate in the reverse direction. Although this architecture enhances charge separation, it also reduces the redox strength of both carriers since electrons in the MOF become less reducing. As a result, type‐II junctions are more commonly applied in pollutant degradation or CO_2_ reduction processes, where moderate redox power is sufficient rather than in high‐efficiency HER systems. Some hybrids, including Ni‐MOF/MnCdS [120] and CIZS/Ni‐MOF [127], exhibit p–n or quasi‐type‐II behavior, where the MOF acts as an electron mediator or energy relay platform. The metallic centers (Ni^2+^, Cu^2+^) within the MOFs contribute directly as active sites for hydrogen production, promoting charge transfer across metal–sulfur coordination bridges (Ni─S, Cu─S) that enhance conductivity and suppress photocorrosion, an essential advantage for sulfide‐based photocatalysts [120, 127].

### 2.4 MOF/Iron Oxide‐Based Multimetal Partner Composites

**FIGURE 4:**
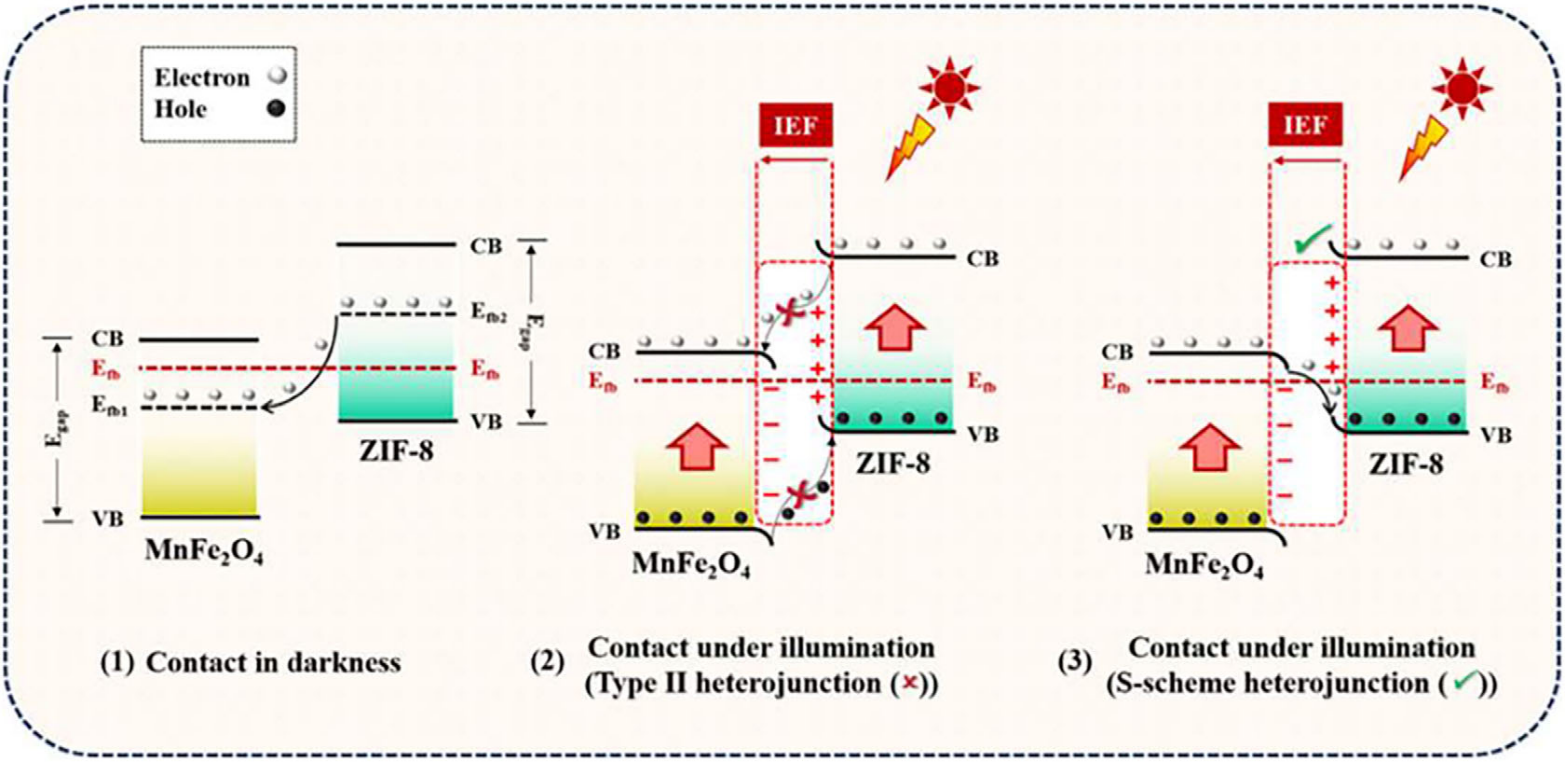
Schematic illustration for probable charge transfer pathway and photocatalytic mechanism of TC disintegration using MnFe_2_O_4_/ZIF‐8 photocatalyst with Xenon light irradiation. Reproduced with permission [134]. Copyright 2024, Elsevier.

Ferrites have demonstrated significant potential in photocatalytic applications due to their unique properties, including high stability, suitable band gap, magnetic capacity, efficient light absorption, ease of synthesis, and compatibility with a wide range of materials [128]. Despite their advantages, ferrites face limitations such as particle agglomeration and low surface area which hinder their photocatalytic efficiency [129]. To address these challenges, researchers have explored coupling ferrites with MOFs, which offer high‐surface areas, tunable porosity, and semiconductor‐like behavior [130]. The synergistic interaction between ferrites and MOFs not only mitigates agglomeration but also enhances charge separation and light absorption, as demonstrated by the reduced band gaps of composite materials. The coupling of MOFs and ferrites can facilitate the formation of Z‐scheme or S‐scheme charge transfer processes as the Fermi level and band structures of MOFs and ferrites differ, enabling efficient charge separation and transfer within the composite [52]. The most notable characteristic of ferrites is their magnetic properties [131, 132]. Therefore, MFe_2_O_4_@MOF composites possess inherent magnetic properties, allowing separation from reaction mixtures through the application of an external magnetic field [133]. Studies have reported that MFe_2_O_4_@MOF heterojunctions generally exhibit narrower band gaps than the corresponding pristine MOFs, contributing to enhanced visible‐light absorption. For instance, as exhibited in Figure 4, Moslem Azqandi et al. synthesized a MnFe_2_O_4_/ZIF‐8 composite that was interpreted as an S‐scheme heterojunction, achieving a significant reduction in the band gap from 5.19 eV (pristine ZIF‐8) to 1.53 eV (composite). This reduction markedly enhanced visible light absorption and improved the efficiency of tetracycline (TC) degradation [134]. According to Figure 4, the Fermi level of ZIF‐8 is higher than that of the MnFe_2_O_4_ semiconductor. Therefore, when ZIF‐8 is in contact with MnFe_2_O_4_ without light irradiation, electrons are transferred from ZIF‐8 with a smaller flat band potential (E_fb_) to MnFe_2_O_4_ with a larger E_fb_ until the two semiconductors reach equilibrium, and a new Fermi level is formed. Similarly, Jianan Liu et al. reported that the ZnFe_2_O_4_/UiO‐66‐NH_2_ composite exhibited a band gap of 2.39 eV, compared to 2.84 eV for pure UiO‐66‐NH_2_, leading to enhanced photocatalytic activity for alcohol oxidation [52].

Bismuth ferrite, another iron‐based oxide semiconductor, encompasses structural variants such as perovskite, mullite, and sillenite [135, 136]. Among these, the perovskite‐type BiFeO_3_ has attracted considerable attention as a photocatalyst due to its favorable properties, including a suitable band gap of 2.2 eV, as well as its unique combination of ferroelectric, ferromagnetic, and chemical characteristics [136, 137]. However, its practical application in photocatalysis is limited by challenges such as low adsorption capacity, short carrier lifetimes, and limited surface area [138, 139]. To address these limitations, coupling BiFeO_3_ with porous materials, particularly MOFs, has emerged as an effective strategy. This approach not only reduces electron–hole recombination rates but also enhances adsorption capacity and contributes to the reduction of the MOF's band gap, resulting in significantly improved photocatalytic performance. For instance, Jamshid and coworkers synthesized a BiFeO_3_@ZIF‐67 nanocomposite via the “bottle‐around‐ship” method and evaluated its photocatalytic efficiency for moxifloxacin (MXF) degradation. Their results show that due to the synergic effect between MOF and BiFeO_3_, the BiFeO_3_@ZIF‐67 nanocomposite exhibited superior photocatalytic performance, achieving 87% MXF degradation, higher than its components, BiFeO_3_ (64.25%) and ZIF‐67 (60.64%) [138]. In MOF/ferrite heterojunctions, the dominant charge–transfer mechanisms are Z‐scheme and S‐scheme. Ferrites such as ZnFe_2_O_4_, CoFe_2_O_4_, MnFe_2_O_4_, and BiFeO_3_ form built‐in electric fields when interfaced with MOFs like UiO‐66‐NH_2_, MIL‐101(Fe), ZIF‐8, or NH_2_‐MIL‐53(Fe) due to differences in work functions and Fermi levels. Upon contact, electron migration causes band bending at the interface, creating an internal electric field that directs the recombination of the low‐energy carriers (electrons in the ferrite CB and holes in the MOF VB) while preserving the high‐energy electrons in the MOF and strong oxidative holes in the ferrite. These preserved redox carriers enable oxidation of OH^−^/H_2_O to ^**.**^OH at the ferrite VB and reduction of O_2_ to O_2_^−^ at the MOF CB, which matches the observed photocatalytic activities such as alcohol oxidation and antibiotic degradation [52, 53, 134, 139]. Type‐II transfer is ruled out because it cannot generate these radicals based on the actual band potentials [52, 139]. Overall, the incorporation of ferrites and BiFeO_3_ into MOFs is a strategic approach for band gap engineering and interfacial charge management. By exploiting the electrochemical potential mismatch between these components, such heterogeneous junctions effectively transition from conventional type‐II mechanisms to more efficient Z‐ or S‐ pathways, leading to the favorable charge transfer and separation.

### 2.5 MOF/Titanium Dioxide‐Based Multimetal Partner Composites

**FIGURE 5:**
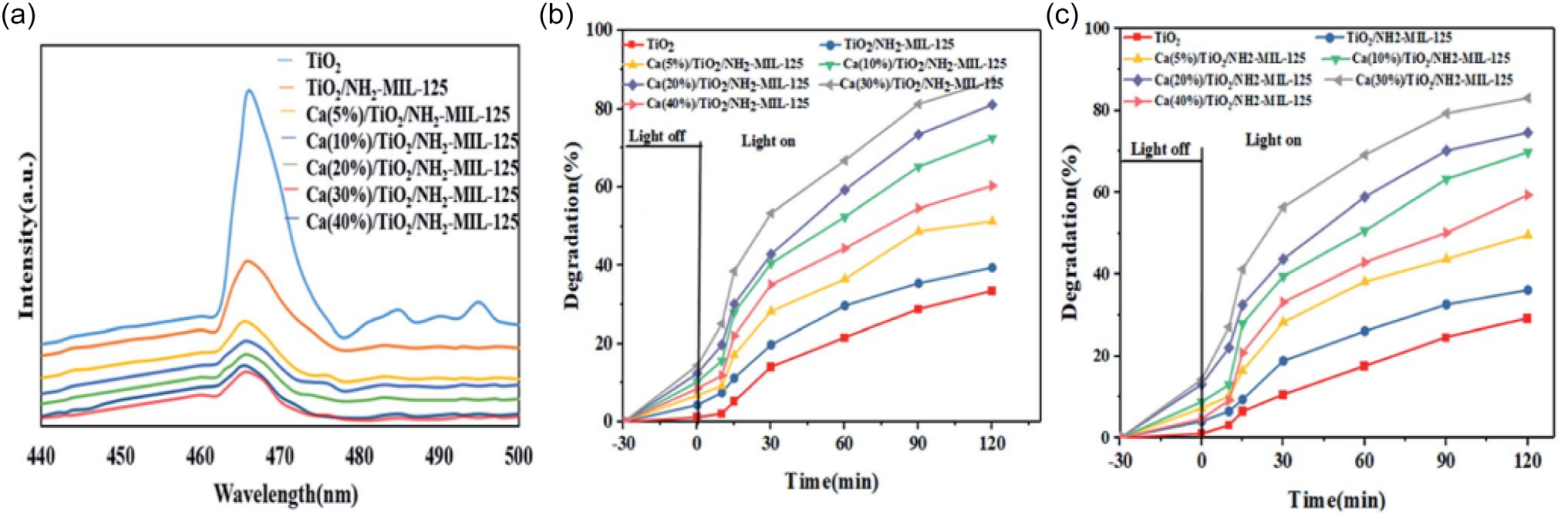
(a) Photoluminescence (PL) analysis of Ca/TiO_2_/NH_2_‐MIL‐125 photocatalyst, visible light induced photodegradation efficiency of Ca/TiO_2_/NH_2_‐MIL‐125 photocatalyst over a 120 min time period for (b) MO and (c) RhB. Reproduced with permission [144]. Copyright 2020, Royal Society of Chemistry.

Titanium dioxide (TiO_2_) is widely recognized for its excellent photocatalytic activity, attributed to its unique properties such as high stability, low cost, nontoxicity, environmental compatibility, and a sufficiently positive VB potential (approximately 2.9 V) [140]. Despite these advantages, TiO_2_ faces significant limitations that hinder its photocatalytic efficiency. A major drawback is the high recombination rate of photogenerated electron–hole pairs, which results in a low quantum efficiency. Additionally, its wide band gap restricts its activation to UV light, limiting its applicability in visible‐light‐driven photocatalytic processes [141]. To address these challenges, TiO_2_ is often coupled with other materials, particularly semiconductors with well‐matched band gaps, to develop visible‐light‐responsive photocatalysts. Among these, MOFs have emerged as promising candidates. Furthermore, since photocatalytic reactions primarily occur on the catalyst's surface, the high surface area provided by MOFs facilitates better interaction with reactants, prevents TiO_2_ particle agglomeration, and improves the catalytic efficiency [142, 143]. Additionally, in such hybrid systems, the presence of metal NPs can act as cocatalysts, significantly enhancing photocatalytic performance [144]. For instance, introducing electron‐accepting cocatalysts (e.g., Ag, Pt, Pd, or Au) into TiO_2_ leads to the creation of additional cathodic sites, thereby improving photocatalytic performance‐particularly in HER [145]. Furthermore, plasmonic photocatalysis utilizing noble metal NPs dispersed in semiconductors like TiO_2_ offers advantages like the formation of a Schottky junction and localized surface plasmon resonance (SPR). These synergistic effects promote efficient charge transfer, minimize the electron–hole recombination rate, and enhance visible light absorption [144, 146]. For instance, decorating TiO_2_ with Ag NPs has been shown to enhance electron–hole separation and significantly improve light absorption in the visible region. Coupling Ag‐doped TiO_2_ with MOFs further facilitates electron transfer, leading to enhanced photocatalytic performance [147, 148]. For example, Mohaghegh et al. prepared MIL‐88B(Fe)/Ag TiO_2_ nanotubes/Ti plates for methyl orange (MO) degradation. They proved that a final composite exhibit 90% dye degradation efficiency, whereas MIL‐88B(Fe)/TiO_2_ nanotubes/Ti plates and pure TiO_2_ nanotubes/Ti plates achieved only 46.1% and 44.5% degradation, respectively [148]. On the other hand, Liu et al. synthesized Au–TiO_2_@NH_2_‐UiO‐66 and demonstrated that Au NPs within this heterojunction serve as electron mediators, significantly enhancing the transfer of photogenerated electrons and thereby improving the overall photocatalytic performance of the composite. They showed degradation performance of ethyl acetate for Au–TiO_2_@NH_2_‐UiO‐66, where 85% was 12.1, 10.6, and 2.83 times higher than that of TiO_2_, NH_2_‐UiO‐66, and TiO_2_@NH_2_‐UiO‐66, respectively [141]. In another study, Ahmadpour et al. developed Ca/TiO_2_/NH_2_‐MIL‐125 and revealed that incorporating alkaline‐earth metals increases the number of surface reactive sites on TiO_2_, facilitating adsorption and promoting charge separation. As demonstrated in Figure 5, this enhancement results in greater photocatalytic efficiency and also a reduction of the photogenerated electron–hole recombination rate of the final composite in the degradation of organic dyes [144].

### 2.6 MOF/LDH Composites

LDHs possess several advantageous properties, such as a large surface area, strong ion‐exchange capabilities, broad photoresponse, efficient electron‐transfer pathways, and abundant structural defects [149, 150, 151]. However, despite these benefits, LDHs face several limitations such as high recombination rate of electron–hole pairs, wide band gap, poor crystalline structure, and low electrical conductivity, which significantly restrict their photocatalytic efficiency [152, 153, 154]. Moreover, the aggregation of irreversibly exfoliated nanosheets within LDHs obstructs the migration of photoexcited electrons and holes, ultimately suppressing photocatalytic reactions [153]. Establishing heterojunctions between two types of semiconductor photocatalysts is a promising strategy to overcome these limitations and enhance the photocatalytic activity [155, 156, 157]. Heterojunctions can be incorporated into MOF/LDH‐based materials, facilitating the separation of photogenerated electron–hole pairs. The interlayer spaces of LDHs not only provide a supportive environment for organic ligands but also act as sites for the nucleation and growth of MOFs. Hence, using LDHs as substrates for MOF growth is expected to harness the advantages of both parent materials, producing functional composites with significant potential [154]. Furthermore, the metal atoms on the surface of LDHs exhibit unsaturated coordination, which serves as activation sites for directed epitaxial growth [149, 150]. As a result, the combined benefits of the large surface area of MOFs and the efficient electron transport channels of LDHs endow the resulting composites with outstanding photocatalytic performance [158, 159]. A wide variety of MOF/LDH composites have been extensively studied as photocatalysts for diverse reactions. For instance, Maseeh and coworkers synthesized Zn‐MOF/MgAl‐LDH through the in situ nucleation of Zn‐MOF nanostructures on MgAl‐LDH nanosheets based on their complementary electronic properties and opposite surface potentials. Their results demonstrated that the MgAl‐LDH/Zn‐MOF (129 mmol g^−1^) composite exhibited enhanced photocatalytic H_2_ production activity compared to the individual Zn‐MOF (85.07 mmol g^−1^) and MgAl‐LDH (27.82 mmol g^−1^) components. This showed 1.52‐ and 4.65‐fold improvements in activity relative to Zn‐MOF and MgAl‐LDH alone, respectively [152].

Typical LDHs, such as NiAl‐LDH, CoAl‐LDH, NiFe‐LDH, ZnTi‐LDH, and MgAl‐LDH, when coupled with MOFs like ZIF‐67, MIL‐53(Fe), MIL‐88B(Fe), or Zn‐MOFs, an interfacial charge redistribution occurs. Electrons migrate between the LDH and the MOF until Fermi‐level equilibration is achieved, forming electron‐rich and electron‐poor regions on opposite sides of the interface [152, 155, 159]. This charge migration establishes an IEF that induces band bending and provides a built‐in driving force for directional carrier motion [153, 160, 161]. The preference for the S‐scheme and Z‐scheme over type‐II transfer arises from four key factors: the preservation of strong redox potentials across both sides of the heterojunction, the suppression of electron–hole recombination losses, the utilization of internal electric fields as self‐sustaining carrier‐separation forces, and the creation of spatially distinct active sites that align with the multistep nature of HER and CO_2_ conversion [153, 155, 159, 162].

### 2.7 MOF/Perovskite Composites

Perovskites can have exceptional properties in photocatalysis, such as a tunable band gap, high charge carrier mobility, and strong light absorption, making them highly effective for solar‐driven photocatalysis [163, 164]. However, despite their promising attributes, metal halide perovskites face significant challenges in photocatalytic applications. Their sensitivity to moisture limits their stability, and issues such as low CO_2_ capture efficiency restrict their use primarily to CO_2_ reduction. To address these limitations, coupling perovskites with MOFs has emerged as a promising strategy for developing hybrid photocatalysts with enhanced performance. In such composite systems, MOFs act as cocatalysts, improving charge separation, suppressing agglomeration, and leveraging their high surface area and porosity to facilitate CO_2_ adsorption and accelerate reaction kinetics [164, 165]. Furthermore, MOFs provide numerous highly active sites, significantly boosting the photocatalytic activity and efficiency of the composite [164]. In terms of band gap engineering, the integration of perovskites with MOFs effectively optimizes the band gap properties of MOFs, enhancing their photocatalytic performance. The formation of heterogeneous junctions in a stepped manner at the contact between the perovskite and MOF causes the photoexcited electrons to transfer from the CB of the perovskite at a higher position to the CB of the MOF at a lower position. In this way, a larger driving force is provided for the electron transfer from a perovskite layer to the MOF, and the photocatalytic performance is enhanced. For instance, the CsPbBr_3_ QDs/UiO‐66 NH_2_ (QDs: quantum dots) nanojunction exhibits a band gap of 2.38 eV compared to 2.89 eV for the pristine MOF [166]. Similarly, the Cs_2_AgBiBr_6_ QDs/Ce‐UiO‐66‐H composite achieves a reduced band gap of 2.11 eV, whereas the bare MOF possesses a band gap of 2.43 eV [167]. Numerous studies have reported the successful synthesis of various MOF/perovskite composites for photocatalytic CO_2_ reduction. For example, Xi et al. synthesized a CsPbBr_3_/2D‐Ni‐based MOF and demonstrated that the enhanced photocatalytic activity stems from improved charge separation and elevated CO_2_ adsorption/activation capacity facilitated by the MOF. Additionally, the elongation of CsPbBr_3_ increases the Δ*E* between the CB of CsPbBr_3_ and the MOF, creating a larger driving force for electron transfer from CsPbBr_3_ to the MOF and significantly enhancing the photocatalytic performance [164]. Moreover, Asadi et al. demonstrated that coupling CsPbBr_3_ with MOF‐808 significantly improves the stability of the perovskite in aqueous environments. The MOF‐808 protective shell effectively shields CsPbBr_3_ from harsh external conditions, enhancing its stability and enabling its use in the photocatalytic degradation of Cr(VI) [168].

Halide perovskites possess broad visible‐light absorption and highly negative CBs, endowing them with strong electron‐donating ability for reduction reactions such as CO_2_ to CO, CH_4_, and C_2_H_6_ [163, 164, 169]. On the other hand, many MOFs feature relatively positive VB or HOMO levels, enabling them to act as efficient hole collectors [163, 168]. When these two semiconductors come into contact, their Fermi‐level equilibration generates an IEF that drives charge redistribution. In the resulting Z‐ or S‐scheme configuration, the less energetic electrons and holes recombine at the interface, leaving highly reducing electrons on the perovskite CB and strongly oxidizing holes on the MOF VB [163, 168, 170, 171, 172]. This charge arrangement enhances both the reduction and oxidation processes, promoting efficient CO_2_ photoreduction, Cr(VI) reduction, and organic pollutant oxidation under visible‐light irradiation. In contrast, certain composites such as CsPbBr_3_/2DNi‐MOF and Cs_2_AgBiBr_6_/UiO‐66‐NH_2_ exhibit type‐II heterojunction behavior, arising when the MOF's CB lies below that of the perovskite. In these cases, electrons transfer from the perovskite CB to the MOF, while holes migrate in the opposite direction, facilitating efficient carrier separation but diminishing redox power [164, 167]. As a result, these type‐II structures are typically optimized for selective CO production, which requires moderate reduction strength, rather than for multielectron processes such as CH_4_ or H_2_ generation that demand stronger redox potentials. Z‐ and S‐scheme pathways dominate in MOF/perovskite heterojunctions because they simultaneously preserve the high reduction potential of the perovskite CB and maintain the strong oxidation ability of the MOF valence states, while the IEF promotes directional charge migration and effectively suppresses electron–hole recombination; together, these features allow the system to support multielectron reactions that demand substantial redox energy [163, 171, 173].

### 2.8 Summary of Section

In summary, SMOFs/multimetallic partner composites, Table 1 indicates a prevalence of reported composites in various photocatalytic procedures. In degradation applications, MOF/Bi_2_MoO_6_, MOF/MVO4, MOF/ferrite, and MOF/TiO_2_‐based multimetal partner composites have all shown promising photocatalytic performance. However, because these studies were carried out under different experimental conditions, including variations in light source, catalyst loading, pollutant concentration, and reaction time, direct comparison of their photocatalytic activities should be made with caution. Moreover, the unique properties of MOF/bimetallic sulfide composites explain the widespread use of these composites as photocatalysts for H_2_ production. The sulfur atoms are generally considered active sites for the adsorption and desorption of oxygen‐containing intermediates, thereby helping to reduce the reaction energy barrier [73]. Many studies on MOF/perovskite and MOF/LDH composites have focused on photocatalytic CO_2_ reduction, suggesting that these materials also warrant investigation in other photocatalytic applications. This implies that further research may investigate the application of these materials in additional photocatalytic reactions. Furthermore, among single metallic‐organic framework/multimetallic partner heterojunction photocatalysts, Z‐scheme and S‐scheme charge–transfer pathways are the mechanisms most frequently proposed in the literature. These models have been widely invoked to explain the combination of efficient charge separation and the preservation of strong redox capability, which are often associated with the superior photocatalytic performance of these composites. While many studies have interpreted the observed photocatalytic behavior based on Mott–Schottky analysis, ESR spectroscopy, fluorescence quenching, in situ XPS, ultraviolet photoelectron spectroscopy (UPS), and related characterization techniques, these methods generally provide indirect evidence and may not, individually, unambiguously distinguish Z‐scheme or S‐scheme mechanisms from alternative charge–transfer pathways, such as type‐II heterojunctions. Consequently, the proposed mechanistic assignments should be interpreted with appropriate caution and, where possible, supported by complementary experimental approaches and theoretical calculations. Nevertheless, the widespread application of Z‐scheme and S‐scheme models highlights their importance as valuable conceptual frameworks for understanding and designing high‐performance MMOF‐based photocatalysts.

**TABLE 1:** List of SMOFs/multimetallic partner composites and their photocatalytic performances.

Name	Metals	Band gap (eV)	Application	Performance	References
UiO‐66(Zr)/Bi_2_MoO_6_	Zr/Bi/Mo	—	Degradation	96% RhB	[81]
NH_2_‐MIL‐125 (Ti)/Bi_2_MoO_6_	Ti/Bi/Mo	1.89	Degradation	93.28% dichlorophen/92.19% trichlorophenol	[78]
Bi‐MOF‐M/Bi_2_MoO_6_	Bi/Mo	2.79	Degradation	93.6% TC	[82]
MOF‐5/Bi_2_WO_6_	Zn/Bi/W	—	Degradation	99.31% RhB	[75]
NH_2_‐MIL‐88B(Fe)/Bi_2_WO_6_	Fe/Bi/W	3.18	Degradation	89.4% TC	[174]
MIL‐88A(Fe)/Bi_2_WO_6_	Fe/Bi/W	2.5	Degradation	96% RhB and 70.6% TC	[76]
Ni‐MOF/Bi_2_WO_6_	Ni/Bi/W	—	Degradation	98.8% MB	[83]
UiO‐66‐NH_2_/Bi_2_MoO_6_	Zr/Bi/W	2.64	Degradation	100% OFL/96% CIP	[85]
UiO‐66‐NH_2_/Bi_2_WO_6_	Zr/Bi/W	2.24	Degradation	100% RhB/88% phenol	[84]
UiO‐66‐NH_2_/Bi_2_WO_6_	Zr/Bi/W	—	Degradation	99.7%. RhB	[175]
MIL‐88B(Fe)/Bi_2_WO_6_	Fe/Bi/W	2.55	Degradation	96.4% TC	[86]
MIL‐101(Fe)/Bi_2_WO_6_	Fe/Bi/W	2.77	Degradation	81% TC	[176]
MIL‐125(Ti)/Bi_2_WO_6_	Ti/Bi/W	—	Degradation	73% TC	[177]
NH_2_‐MIL‐68(In)/Bi_2_WO_6_	In/Bi/W	2.16	Degradation	84.3% LEV	[178]
Bi‐MOF/Bi_2_WO_6_	Bi/W	3.06	Degradation	96% TC/80% RhB	[153]
NH_2_‑MIL‑125(Ti)/Bi_2_WO_6_	Ti/Bi/W	—	Degradation	98% RhB	[88]
ZIF‐8/Bi_2_MoO_6_	Zn/Bi/W	—	Degradation	66.88% MB	[179]
UiO‐66/Bi_2_WO_6_	Zr/Bi/W	—	Degradation	Almost 100% RhB	[180]
CAU‐17/Bi_2_WO_6_	Bi/W	2.34	Degradation	80% RhB	[181]
NH_2_‐MIL‐53(Fe)/BiVO_4_	Fe/Bi/V	2.46	Degradation	90.2% MB/80.0% CIP	[182]
CAU‐17/BiVO_4_	Bi/V	2.4	Degradation	99.3% RhB/97.7% CR	[92]
ZIF‐67/Ag_3_VO_4_	Co/Ag/V	—	Degradation	98.5% VEN	[102]
MIL‐125‐NH_2_/Ag_3_VO_4_	Ti/Ag/V	2.27	Degradation	92.0% MB and 73.8% RhB	[183]
MIL‐100(Fe)/BiVO_4_	Fe/Bi/V	—	Degradation	88% BR46	[94]
ZIF‐8/BiVO_4_	Zn/Bi/V	—	Degradation	80% MB	[184]
HKUST‐1‐MOF/BiVO_4_	Cu/Bi/V	1.86	Degradation	99.98% DB and 98.76% Rb	[185]
Ce MOF/mcBiVO_4_	Ce/Bi/V	2.2	Degradation	88.8% MB, 86.07% RhB	[103]
ZIF‐8/Ag_3_VO_4_	Zn/Ag/V	2.11	Degradation	60% RhB	[99]
MIL‐101(Cr)/Cd_0:8_Zn_0:2_S	Cr/Cd/Zn	—	Degradation	99.2% MB	[111]
UiO‐66‐(SH)_2_/ZnIn_2_S_4_	Zr/Zn/In	—	Degradation	96% RhB	[186]
ZIF‐67/Cd_0.5_Zn_0.5_S	Co/Cd/Zn	1.33	Degradation	96.6% MV	[110]
ZIF‐8/NiFe_2_O_4_	Zn/Ni/Fe	—	Degradation	94% MB	[187]
MIL‐53(Fe)/CoFe_2_O_4_	Fe/Co	2.1	Degradation	99.35% DR23	[131]
Cu ‐MOF/MgFe_2_O_4_	Cu/Mg/Fe	—	Degradation	94.71% RB and 97.90% Rh6G	[188]
MIL‐53(Fe)/NiFe_2_O_4_	Fe/Ni	—	Degradation	95.18% RhB	[132]
UiO‐66(Zr)/MgFe_2_O_4_	Zr/Mg/Fe	2.28	Degradation	94% TC	[133]
MIL‐101(Fe)/CoFe_2_O_4_	Fe/Co	2.46	Degradation	80% TC	[53]
NH_2_‐MIL‐53(Fe)/N‐doped BiFeO_3_	Fe/Bi	1.85	Degradation	99% TC‐H/76% CIP/91% sulfamethoxazole	[139]
ZIF‐8/MnFe_2_O_4_	Zn/Mn/Fe	1.53	Degradation	75.4% TC	[134]
NH_2_‐MIL‐125(Ti)/CoFe_2_O_4_	Ti/Co/Fe	2.98	Degradation	68.2% COD, 21.1% DOX/24.5% 5‐FU	[189]
Co‐TiO_2_@ZIF‐8	Co/Ti/Zn	1.56	Degradation	42% thiosulfate	[142]
AgI@TiO_2_/UiO‐66 NH_2_	Ag/Ti/Zr	2.30	Degradation	82.9% RhB	[147]
Ca/TiO_2_/NH_2_‐MIL‐125	Ca/Ti	—	Degradation	82.87% RhB/86.22% MO	[144]
Au–TiO_2_@NH_2_‐UiO‐66	Au/Ti/Zr	2.81	Degradation	85% ethyl acetate	[141]
Ag/TiO_2_/MIL‐88B(Fe)	Ag/Ti/Fe	—	Degradation	100% *E. coli*/90% MO	[148]
MIL‐88B(Fe)/ZnTi‐LDH	Fe/Zn/Ti	—	Degradation	93.51% TC	[190]
TMU‐5/NiTi‐LDH	Zn/Ni/Ti	—	Degradation	98% SMZ	[149]
Cu‐MOF/NiAl‐LDH	Cu/Ni/Al	1.38	Degradation	99% MO	[150]
MIL‐53(Al)/MgFe‐LDH@HC	Al/Mg/Fe	1.55	Degradation	99% TC/99% betamethasone/99% oxytetracycline/99% metsulfuron methyl/99% nicosulfuron	[191]
Metal oxide QDs immobilized‐MOF‐5/NiCo‐LDH	Zn/Ni/Co	2.99	Degradation	96.2% MB, 95.8% RB/99.6% MG	[192]
Ce‐MOF/Bi_2_MoO_6_	Ce/Bi/W	2.71 and 3.31	CO_2_ reduction	113.87 μmol g^−1^ h^−1^ CH_4_/40.59 μmol g^−1^ h^−1^ CH_3_OH/73.48 μmol g^−1^ h^−1^ HCOOH	[193]
NH_2_‐MIL‐101(Fe)/Bi_2_MoO_6_	Fe/Bi/Mo	—	CO_2_ reduction	67 μmol g^−1^ h^−1^ to CO	[80]
ZIF‐8/ZnAl‐LDH	Zn/Al	3.11	CO_2_ reduction	9.03 μmol g^−1^ h^−1^	[162]
MIL‐100/NiMn‐LDH	Mn/Ni	1.98	CO_2_ reduction	2.84 μmol h^−1^ to CH_4_	[158]
MOF‐199/ZnAl‐LDH	Cu/Zn/Al	—	CO_2_ reduction	89.45 μmol g^−1^	[194]
2D‐Ni‐MOF/CsPbBr_3_	Ni/Cs/Pb	—	CO_2_ reduction	81.0 μmol g ^−1^ h^−1^ to CO	[164]
UiO‐66‐NH_2_/CsPbBr_3_	Zr/Cs/Pb	2.87	CO_2_ reduction	98.57 μmol g^−1^ to CO	[166]
Ce‐UiO‐66‐H/Cs_2_AgBiBr_6_	Ce/Cs/Ag/Bi	2.29	CO_2_ reduction	309.01 μmol g^−1^ h^−1^ to CO	[167]
UiO‐66‐NH_2_/Cs_2_AgBiBr_6_	Zr/Cs/Ag/Bi	—	CO_2_ reduction	13.4 μmol g^−1^ h^−1^ for CO and 0.72 μmol g^−1^ h^−1^ for CH_4_	[173]
ZIF‐67/CsPbBr_3_	Co/Cs/Pb	—	CO_2_ reduction	29.63 µmol g^−1^ h^−1^	[165]
ZIF‐8/CsPbBr_3_	Zn/Cs/Pb			15.50 µmol g^−1^ h^−1^	
ZIF‐9‐III/Cs_3_Cu_2_I_5_	Co/Cs/Cu	—	CO_2_ reduction	110 mmol g^−1^ h^−1^ to CO and 5 mmol g^−1^ h^−1^ to CH_4_	[169]
Ni‐MOF–74/BiVO_4_	Ni/Bi/V	—	H_2_ production	245.4 μmol	[98]
Ni‐MOF/CuInZnS	Ni/Cu/In/Zn	—	H_2_ production	2642 μmol g^−1^ h^−1^	[127]
NH_2_‐MIL‐125/CdIn_2_S_4_	Ti/Cd/In	2.17	H_2_ production	2550 μmol g^−1^ h^−1^	[195]
NH_2_‐MIL‐125(Ti)/ZnIn_2_S_4_/CdS	Ti/Zn/In/Cd	1.84	H_2_ production	2.367 mmol g^−1^ h^−1^	[113]
ZIF‐8/ZnIn_2_S_4_	Zn/In	2.07	H_2_ production	5208 μmol g^−1^ h^−1^	[112]
UiO‐66/ZnIn_2_S_4_	Zr/Zn/In	2.55	H_2_ production	3061.61 μmol g^−1^ h^−1^	[119]
NH_2_‐MIL‐125(Ti)/ZnIn_2_S_4_	Ti/Zn/In	2.40	H_2_ production	2204.2 μmol g^−1^ h^−1^	[196]
MIL‐68(In)/ZnIn_2_S_4_	In/Zn	—	H_2_ production	28.2 mmol g^−1^ h^−1^	[115]
Cu‐MOF/ZnIn_2_S_4_	Cu/Zn/In	—	H_2_ production	0.3 mmol g^−1^ h^−1^	[125]
UiO‐66‐NH_2_/ZnIn_2_S_4_	Zr/Zn/In	—	H_2_ production	5275 μmol g^−1^ h^−1^	[197]
MIL‐88A/ZnIn_2_S_4_	Fe/Zn/In	—	H_2_ production	1259.63 μmol g^−1^ h^−1^	[121]
MOF‐5/Zn_0.2_Cd_0.8_S	Zn/Cd	—	H_2_ production	15.08 mmol g^−1^ h^−1^	[198]
ZIF‐67/ZnCdS	Co/Zn/Cd	—	H_2_ production	23264.6 μmol g^−1^ h^−1^	[105]
Cu‐MOF/Cd_0.5_Zn_0.5_S	Cu/Cd/Zn	—	H_2_ production	18986 μmol g^−1^ h^−1^	[123]
DUT‐67/NiS/CdS	Zr/Ni/Cd	—	H_2_ production	9618 μmol g^−1^ h^−1^	[199]
Thiol‐UiO‐66/Mn_0.5_Cd_0.5_S	Zr/Mn/Cd	2.12	H_2_ energy recovery	3179.36 μmol g^−1^ h^−1^	[200]
MIL‐88A(Fe)/CdLa_2_S_4_	Fe/Cd/La	—	H_2_ production	7677.5 μmol g^−1^ h^−1^	[122]
MIL‐125‐NH_2_(Ti)/Zn_0.5_Cd_0.5_S	Ti/Zn/Cd	—	H_2_ production	92498 μmol g^−1^ h^−1^	[201]
Ni‐MOF‐74/Cd_*x*_Zn_1−*x*_S	Ni/Cd/Zn	—	H_2_ production	1712.3 μmol	[109]
UIO‐66(Ce)/ZnCdS	Ce/Zn/Cd	2.21	H_2_ production	1281.1 μmol g^−1^ h^−1^	[202]
Ni‐MOF‐74/Mn_0.2_Cd_0.8_S	Ni/Mn/Cd	—	H_2_ production	7.104 mmol g^−1^ h^−1^	[120]
Cu‐MOF/Mn_0.05_Cd_0.95_S	Cu/Mn/Cd	—	H_2_ production	547.5 mmol g^−1^ h^−1^	[203]
MIL‐101/Au@CdS	Cr/Au/Cd	—	H_2_ production	250 mmol h^−1^/10 mg	[204]
Ni‐MOF/CuFe_2_O_4_	Ni/Cu/Fe	2.0	H_2_ production	570 mmol g^−1^ h^−1^	[205]
UiO‐66‐NH_2_/CuFe_2_O_4_	Zr/Cu/Fe	2.60	H_2_ production	62.5 µmol g^−1^ h^−1^	[206]
ZIF‐67/NiV‐LDH	Co/Ni/V	—	H_2_ production	1599 mmol g^−1^ h^−1^	[207]
Zn‐MOF/MgAl‐LDH	Zn/Mg/Al	1.47	H_2_ production	129 mmol g^−1^	[152]
NH_2_‐UiO‐66/NiCo‐LDH	Zr/Ni/Co	2.30	H_2_ production	2458 μmol g^−1^ h^−1^	[208]
MIL‐53(Fe)/NiAl‐LDH	Fe/Ni/Al	—	H_2_ production	1021.8 μmol g^−1^ h^−1^	[160]
ZIF‐67@NiAl‐LDH	Co/Ni/Al	—	H_2_ production	2.99 mmol g^−1^ h^−1^	[209]
NH_2_‐MIL‐125(Ti)/ZnCr‐LDH	Ti/Zn/Cr	—	H_2_ production	40.8 mmol g^−1^ h^−1^	[210]
MIL‐100(Fe)/BiVO_4_	Fe/Bi/V	—	Water oxidation	333.3 μmol g^−1^ h^−1^	[72]
MIL‐101(Cr)/ZnIn_2_S_4_	Cr/Zn/In	2.32	Cr(VI) reduction	95%	[211]
MIL‐88A(Fe)/ZnIn_2_S_4_	Fe/Zn/In	2.63	Cr(VI) reduction	100%	[212]
MOF‐808/CdIn_2_S_4_	Zr/Zn/In	2.14	Cr(VI) reduction	95.45%	[213]
UiO‐66‐NH_2_/MnFe_2_O_4_	Zr/Mn/Fe	2.5	Cr(VI) reduction	100%	[214]
MIL–101–NH_2_/BiVO_4_	Fe/Bi/V	2.3	Removal	91.2% Cr(VI)	[93]
UiO‐66‐NH_2_/CdIn_2_S_4_	Zr/Cd/In	—	Removal	97.26% Cr(VI)/close to 100% TC	[215]
MOF‐808/NiFe_2_O_4_	Zr/Ni/Fe	3.38	Removal	100% meropenem/100% Cr(VI)	[216]
NH_2_‑MIL‐125/ZnIn_2_S_4_	Ti/Zn/In	—	4‐Nitroaniline reduction to PPD	99%	[217]
NH_2_‐MIL‐101(Fe)/BiVO_4_	Fe/Bi/V	1.56	Nitrate reduction to N_2_	94.8%	[97]
HKUST/Bi_2_WO_6_	Cu/Bi/W	2.7	Benzyl alcohol oxidation to benzaldehyde	85% conversion	[218]
UiO‐66‐NH_2_/ZnFe_2_O_4_	Zr/Zn/Fe	2.39	Benzyl alcohol oxidation to benzaldehyde	68% conversion	[52]
MIL‐53(Fe)/BiFeO_3_	Fe/Bi	2.66	Benzyl alcohol oxidation to benzaldehyde	58.5% conversion	[219]
UiO‐66‐NH_2_/BiVO_4_	Zr/Bi/V	—	Thioanisole conversion	95.21% to sulfoxide	[95]
MIL‐101/CdS‐Ni	Cr/Cd/Ni	—	Dehydrogenation	87.9% N‐heterocycles	[220]
MIL‐101(Fe)/Bi_2_WO_6_	Fe/Bi/W	—	Decontamination	97.2% Cr(VI)/82.8% TC	[87]
UiO‐66‐NH_2_/ZnIn_2_S_4_	Zr/Zn/In	—	Detoxification	96.7% CEES	[221]
PCN‐224/ZnIn_2_S_4_	Zr/Zn/In	1.80	H_2_ production/degradation	0.284 mmol h^−1^ and 99.9% TC	[120]
NH_2_‐UiO‐66/ZnIn_2_S_4_	Zr/Zn/In	—	H_2_ production/degradation	2199 μmol g^−1^ h^−1^/98% MG	[126]
UiO‐66‐NH_2_/MgIn_2_S_4_	Zr/Mg/In	—	H_2_ and O_2_ production	493.8 μmol h^−1^ H_2_ and 258.6 μmol h^−1^ O_2_	[108]
UiO‐66‐NH_2_/Cd_0.2_Zn_0.8_S	Zr/Cd/Zn	—	H_2_ production and CO_2_ reduction	5846.5 mmol g^−1^ h^−1^ and 6.8 mmol g^−1^ h^−1^ CO_2_ to CH_3_OH	[107]
ZIF‐67/CuFe‐LDH	Co/Cu/Fe	1.84	H_2_ production/CO_2_ reduction	7.4 mmol g^−1^ h^−1^/227 μmol g^−1^ h^−1^ to CH_3_OH	[155]
Cs_2_AgBiBr_6_/UiO‐66	Cs/Ag/Bi/Zr	—	CO_2_ reduction/degradation	72.15 μmol g^−1^ to CO, CH_4_, and C_2_H_6_	[163]
				90.5% RhB	
MOF‐808/CsPbBr_3_	Zr/Cs/Pb	2.25	Reduction/degradation	92% Cr(VI) reduction/97% CEF	[168]
				87% ceftazidime	
UiO‐66−NH_2_/CuInS_2_	Zr/Cu/In	—	N_2_ fixation/H_2_O_2_ production	398 μmol g^−1^ h^−1^/4073 μmol g^−1^ h^−1^	[222]

Abbreviations: 5‐FU, 5‐fluorouracil; BR46, Basic Red 46; CEES, 2‐chloroethyl ethyl sulfide; CEF, ceftriaxone; CIP, ciprofloxacin; COD, chemical oxygen demand; DB, disulfine blue; DOX, doxorubicin hydrochloride; MB, methylene blue; MG, malachite green; MV, methyl violet; OFL, ofloxacin; PPD, *p*‐phenylenediamine; RB, Rose Bengal; Rh 6G, rhodamine 6G; RhB, rhodamine B; SMZ, sulfamethazine; TC, tetracycline; VEN, venlafaxine; LEV, levofloxacin.

## 3 Triple Heterojunctions

**FIGURE 6:**
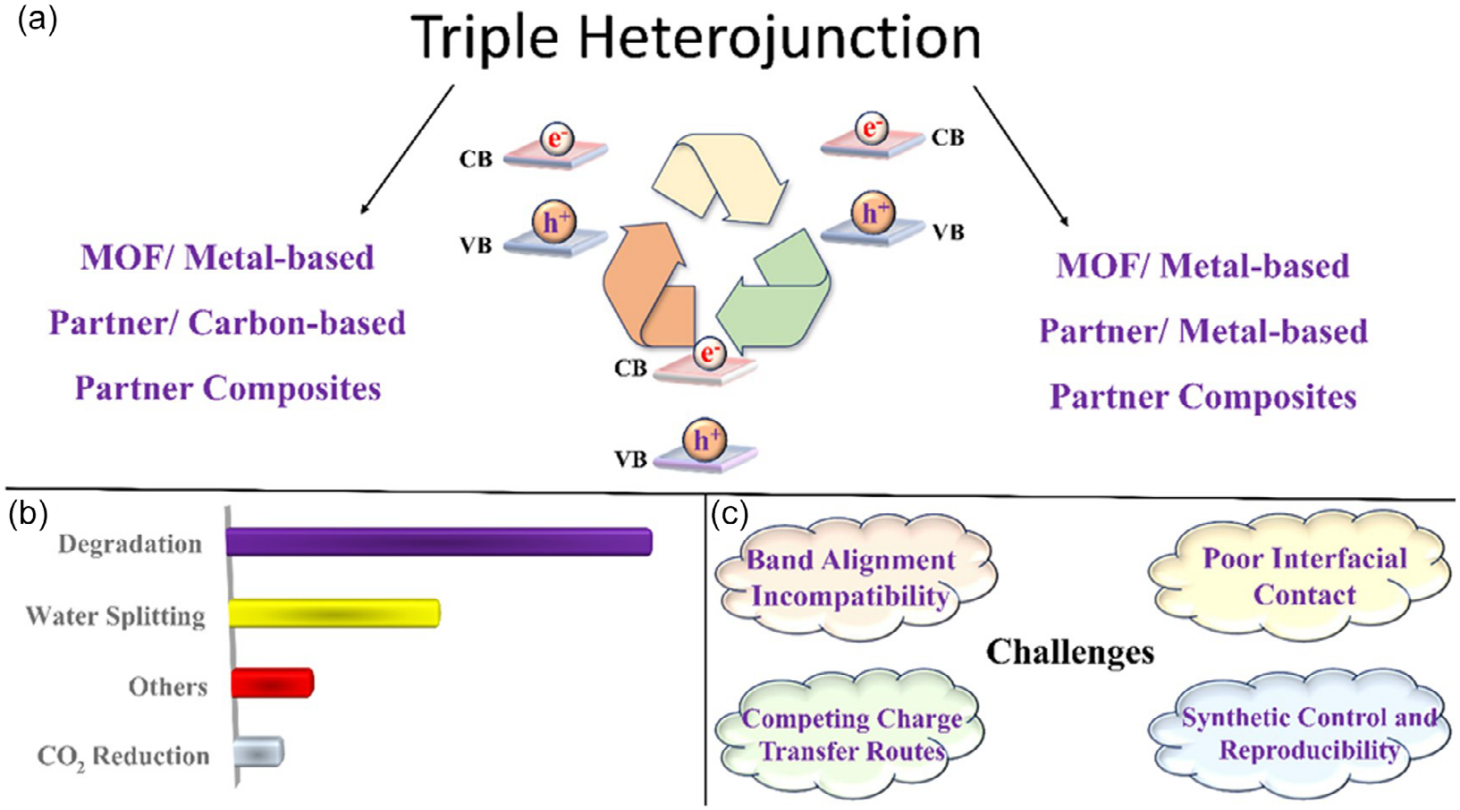
(a) Description of triple heterojunction discussed in this review. (b) Representative applications of triple heterojunction discussed in this review. (c) Illustration of challenges for triple heterojunction in the field of photocatalysis.

MOFs have unique properties, such as a high surface area, accessible active sites, and tunable structural and morphological features. Despite these advantages, MOFs exhibit poor semiconducting properties. Therefore, the use of pristine MOFs for photocatalytic applications is limited [223]. Photocatalysis can be enhanced by employing multiple partners alongside MOFs or MMOFs. As illustrated in Figure 6a, the first form, the partner's components are both metallic. However, in the second form, just one component contains a metallic element, while the other lacks metallic elements and usually consists of nonmetallic materials. To optimize the structure and utilize the advantages of multimetallic semiconductors and MOFs, these compounds are generally combined to create multicomponent MOF‐based partners. The desirable performance in these composites results from the synergistic combination of multiple components and MOFs. Compared to bimetallic composites, the combination of multicomponent, multimetallic partners, and MOFs or MMOFs provides advantages, including varied and adjustable porous structures, greater surface area, enhanced charge transport, and a decreased recombination rate. In this case, the degradation applications in photocatalytic procedures benefit more from triple heterojunctions (Figure 6b) [173]. However, critical challenges also exist in creating triple heterojunctions in the field of photocatalysis that must be noticed more in the future (Figure 6c).

### 3.1 MOF/Metal‐Based Partner/Metal‐Based Partner Composites

Composites containing MIn_2_S_4_ have attracted much attention among the reported compounds for these type of materials in the first form [224, 225, 226, 227, 228]. For example, Bariki et al. reported the synthesis of the multimetallic composite UiO‐66–NH_2_/CdIn_2_S_4_/CaIn_2_S_4_. This composite showed excellent performance for the photocatalytic degradation of asulam. Under a pollutant concentration of 8 ppm and irradiation with a 250 W Xenon lamp, the composite achieved an efficiency of over 93% within 120 min using 20 mg of the catalyst, corresponding to a degradation rate constant of 0.02 min^−1^. In comparison, the individual components exhibited significantly lower efficiencies of 20%–35% under the same conditions. This improved performance was attributed to the strong light‐harvesting ability of CdIn_2_S_4_ and CaIn_2_S_4_ and their narrow band gap. Also, in the resulting composite, electron–hole pair recombination was inhibited, which ultimately increased the photocatalytic activity [226]. In another study, Liu et al. reported the synthesis of the NH_2_‐MIL‐125(Ti)/Ti_3_C_2_ MXene QDs/ZnIn_2_S composite and investigated it for photocatalytic degradation of antibiotics. The composite showed an outstanding performance of 96% in 50 min for TC degradation (concentration: 20 ppm) under a 300 W Xenon lamp using 15 mg of the catalyst, with a rate constant of 0.0487 min^−1^. For SMZ (concentration: 30 ppm), the composite achieved 98% degradation in 40 min under the same light source and catalyst loading, with a rate constant of 0.0731 min^−1^. In this study, the band gap value was reduced from 2.57 eV for the MOF and 2.43 eV for ZnIn_2_S_4_ to 2.16 eV for the composite. Also, the presence of Ti_3_C_2_ MXene QDs in the composite increased the electron transfer between the composite components [227]. In 2022, the use of core–shell composite MOF‐5/CuO@ZnIn_2_S_4_ for the photocatalytic degradation of phenol and 2,4‐dichlorophenol (2,4‐DCP) was reported by Yang et al. The composite achieved a performance of 97.3% for the degradation of phenol and 98.7% for the degradation of 2,4‐DCP (pollutant concentration: 10 ppm) within 120 min under a 300 W Xenon lamp using 30 mg of catalyst. In the resulting composite, the electron–hole pair is separated more effectively, and the creation of a core–shell structure by adding ZnIn_2_S_4_ increased the specific surface area and stability of the composite. In addition, the number of active sites has also increased [228]. In addition to MIn_2_S_4_, several studies have been reported on using Ag_3_PO_4_ in this composite type, indicating that these photocatalysts can be used for the removal and degradation of dyes and pollution [229, 230, 231]. For instance, Mohaghegh et al. synthesized an Ag_3_PO_4_/BiPO_4_/Cu(tpa) graphene composite using Ag_3_PO_4_, which showed excellent performance for the photocatalytic degradation of atrazine. In this study, improved performance can be attributed to the increased surface area of the composite and the improved electron transport due to the presence of Cu(tpa) and graphene. Also, the existence of Ag_3_PO_4_/BiPO_4_ particles creates more possible electron pathways in the composite structure, which creates more active sites and ultimately increases the photocatalytic activity [229]. In another example, the synthesis of the Ag_3_PO_4_/MIL‐101/NiFe_2_O_4_ (APO/MOF/NFO) composite was reported by Zhou et al., which displayed exceptional performance for the degradation of RhB, achieving an efficiency of 93% in 30 min. However, APO and APO/NFO achieved efficiencies of 64% and 71%, respectively, in 20 min. The low efficiency of APO and APO/NFO was attributed to the rapid recombination of electron and hole pairs. The results show that the final composite has a longer fluorescence lifetime, lower impedance, and larger transient photocurrent, which is due to the synergistic effect of the composite components and the rapid charge transfer from APO to NFO facilitated by the 3D framework of the MOF [230]. There are also some studies of the utilization of iron‐ or cobalt‐based MOFs in this class of multimetallic composites for this specific application. For example, Bakhtian et al. employed the multimetallic composite NiCo_2_O_4_@MOF‐801@MIL‐88A for the photocatalytic degradation of Meropenem and achieved an efficiency of 85% in 75 min, which is higher than that of their constituent compounds. The addition of MIL‐88A to the NiCo_2_O_4_@MOF‐801 composition significantly reduced the band gap from 4.1 to 2.55 eV, which had a significant impact on the photocatalytic performance. Additionally, it improved electron transfer and reduced recombination charge carriers in the composite [232]. In a similar example, Hajiali et al. synthesized a ZnO nanorods/Bi_2_MoO_6_/MIL‐101(Fe) composite and investigated it for the photocatalytic degradation of TC. This composite achieved an efficiency of 96.1% in 90 min. The addition of MIL‐101(Fe) to the ZnO/Bi_2_MoO_6_ composite reduced the band gap from 2.48 to 2.3 eV and reduced the recombination of charge carriers, which ultimately led to an increase in the photocatalytic activity of the composite [233]. In another study, Gholizadeh Khasevani et al. synthesized a multimetallic composite BiOI/ZnFe_2_O_4_/MIL‐88B(Fe) containing ZnFe_2_O_4_ NPs. The resulting composite showed greater efficiency than its precursor compounds for the removal of anionic dye (Acid Blue 92), reaching an efficiency of 73% after 120 min [234]. Among cobalt‐based MOFs, ZIF‐67 has been employed more than the other compounds. For example, Flihh et al. synthesized the ZIF‐67@CoWO_4_@CoS composite and utilized it for the photocatalytic degradation of RhB. This composite showed an outstanding performance of 100% after 10 min, which is higher than that of ZIF‐67@CoWO_4_ and ZIF‐67@CoS. This enhancement can be attributed to the high surface area of the composite due to the presence of ZIF‐67 and also the improved optical properties of ZIF‐67 due to the presence of two optical structures CoWO_4_ and CoS, which effectively separate the charge carriers and reduce the recombination rate of the generated charge carriers. In addition, it can also be attributed to the indirect photosensitization of MB and the MB adsorption equilibrium [235]. Hydrogen production is another category of common application for this type of multimetal composite. Multimetal composites based on UiO‐66 have attracted great attention in hydrogen production applications. UiO‐66 is a porous structure consisting of zirconium clusters linked by terephthalates. These compounds display outstanding stability at high‐pressure temperatures and also possess semiconductor properties. For example, Wang et al. reported the synthesis of the Ni_2_P@UiO‐66‐NH_2_/Zn_0.5_Cd_0.5_S composite, which exhibited excellent performance in photocatalytic hydrogen production. The hydrogen production rate reached 40.91 μmol g^−1^ h^−1^, which is 3.3 times larger than that of Zn_0.5_Cd_0.5_S. The addition of Ni_2_P@UiO‐66‐NH_2_ increases the surface area of the final composite and creates abundant reaction sites. In addition, it creates an efficient charge‐transfer channel, which increases the photoelectron‐transfer efficiency of the composite and ultimately improves the photocatalytic activity [236]. In another study, the synthesis of another composite based on UiO‐66 was reported by Pan. The Na_*x*_‐C_3_N_4_/Pt@UiO‐66 composite exhibited good performance for hydrogen production, with approximately 10 times better photocatalytic activity than that of single Na_0.02_‐C_3_N_4_. This improvement is attributed to the increased light‐harvesting ability and more efficient electron transfer, which ultimately led to enhanced photocatalytic activity [237]. In 2024, Biswal et al. reported the synthesis of a composite (TiO_2_/Ti_3_C_2_–TiC/Ce/Zr‐UiO‐66‐NH_2_) that revealed excellent performance for photocatalytic hydrogen production. The results displayed that the visible light H_2_ production rate is 4 times higher than that of TiO_2_/Ti_3_C_2_–TiC. The excellent performance of the photocatalyst was attributed to the large‐surface area, more efficient charge separation, and increased light absorption capacity. Additionally, the addition of Ce/Zr‐UiO‐66‐NH_2_ to the composite increased the active sites for the photocatalytic reaction [238]. In another studies in triple heterojunctions, the successful synthesis of PdS QDs@thiol‐functionalized UiO‐66@Cd_0.5_Zn_0.5_S)PdS@UiOS@CZS) composite was reported by Mao et al. This composite exhibited an outstanding H_2_ production rate of 460.8 μmol h^−1^, This performance is 512 and 9.2 times higher than that of UiOS and CZS, respectively. UiOS acts as a charge‐transport medium for the transport of charge carriers. Additionally, the PdS adsorb the photogenerated holes into the pores of UiOS, and the photogenerated electrons migrate to CZS, resulting in the suppression of the recombination of these charge carriers and the enhancement of the photocatalytic activity [239]. In a similar study, the Au nanodots@thiol‐UiO66@ZnIn_2_S_4_ composite was synthesized and showed exceptional performance for H_2_ production rate of 391.6 μmol h^−1^, which is 61.2 times higher than that for ZnIn_2_S_4_. The reason for this improvement is the more efficient separation and migration of charge carriers, and thiol‐UiO66 acts as a charge‐transfer medium [118]. In a study by Mu et al., the synthesis of UiO‐66‐(COOH)_2_/MoS_2_/ZnIn_2_S_4_ was reported. The composite exhibited outstanding photocatalytic H_2_ production (18.794 μmol h^−1^). The results showed that by combining UiO‐66‐(COOH)_2_ and ZnIn_2_S_4_, the electron–hole transfer between ZnIn_2_S_4_ and UiO‐66‐(COOH)_2_ was enhanced. Also, the surface area was significantly increased compared to that of ZnIn_2_S_4_. The incorporation of MoS_2_ in the composite structure increased the light absorption of the composite, ultimately enhancing the photocatalytic activity due to the high surface area, suitable band gap, and faster transfer of photogenerated electrons [240].

In terms of heterojunctions for MOF/metal‐based partner/metal‐based partner composites, several reported composites have been interpreted as operating through a Z‐scheme charge–transfer mechanism, which has been proposed to enhance redox capability and photocatalytic degradation performance. Collectively, these studies suggest that appropriate band alignment and interfacial engineering may favor a Z‐scheme charge–transfer pathway, thereby preserving stronger oxidative and reductive potentials than would be expected for a conventional type‐II heterojunction. For example, the Ti‐MOF/Ag/NiFe‐LDH composite employs a Z‐scheme mechanism for charge transfer [241]. In the Z‐scheme Ti‐MOF/Ag/NiFe‐LDH system, electrons excited to the CB of the Ti‐MOF are transferred to the VB of NiFe‐LDH via plasmonic Ag NPs, forming intimate interfaces and enhancing charge separation. This results in electron accumulation in the CB of NiFe‐LDH (−0.88 eV vs. NHE) with high reduction potential and hole accumulation in the VB of Ti‐MOF (2.12 eV vs. NHE) with strong oxidizing ability. The accumulated electrons generate abundant ^**.**^O_2_^−^ radicals, which further form ^**.**^OH radicals, while the holes directly oxidize adsorbed pollutants. This proposed charge–transfer pathway is consistent with the enhanced photocatalytic activity reported for dye degradation, demonstrating the efficiency of the Z‐scheme mechanism over conventional type‐II heterojunctions. In Z‐scheme cases, some multimetallic MOF‐based composites have been reported to operate in diverse photocatalytic procedures, including TiO_2_/Ti_3_C_2_–TiC/Ce/Zr‐UiO‐66‐NH_2_ [238], CdS‐P25/ZIF‐67 [242], MOF‐5/CuO@ZnIn_2_S_4_ [228], NH_2_‐MIL‐125(Ti)/Ti_3_C_2_ QDs/ZnIn_2_S_4_ [227], UiO‐66‐(COOH)_2_/MoS_2_/ZnIn_2_S_4_ [240], and BiOI/ZnFe_2_O_4_/MIL‐88B(Fe) [234]. Notably, several related composites also exhibit S‐scheme behavior, such as UiO‐66(–NH_2_)/CdIn_2_S_4_/CaIn_2_S_4_ [226], further emphasizing the importance of band‐engineered heterojunctions in advanced photocatalysis. Unlike most MMOF composites that follow Z‐scheme or S‐scheme pathways, a small group of systems behave as exceptions and rely on the classical type‐II charge–transfer mechanism. In these composites, the two semiconductors feature staggered band positions, so photogenerated electrons drift toward the component with the lower conduction band, while holes migrate to the one with the higher valence band. This alternative route still promotes charge separation and suppresses recombination, though through a fundamentally different layout of energy levels. Representative examples include PdS@UiOS@CZS [239], Au@UiOS@ZIS [118], PCN‐222‐PW_12_/TiO_2_ [243], and ZnO nanorods/Bi_2_MoO_6_/MIL‐101(Fe) [233], each of which has demonstrated efficient performance in H_2_ production or pollutant degradation despite operating through this type‐II mechanism.

### 3.2 MOF/Metal‐Based Partner/Carbon‐Based Partner Composites

**FIGURE 7:**
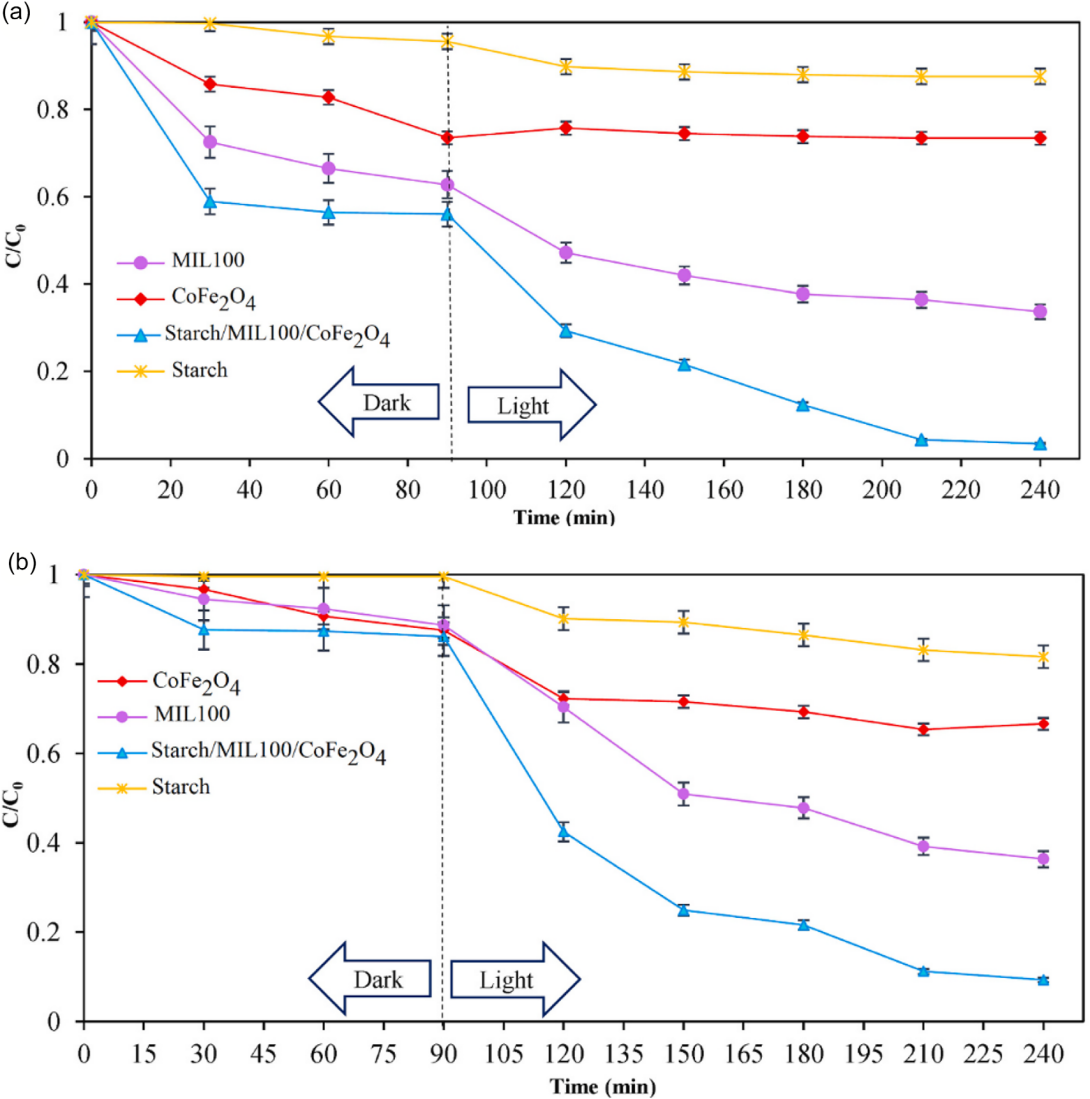
(a) Degradation of MB by composite and its constituent compounds. (b) Degradation of TC by composite and its constituent compounds. Reproduced with permission [248]. Copyright 2024, Elsevier.

This section discusses multimetallic composites based on MOF, which have two partner component that one of them does not contain a metal species [100, 244]. These partners often have a substrate based on graphene or poly(vinylidene fluoride) (PVDF). Graphene is a strong electron acceptor and provides effective charge carrier separation, which, in turn, helps improve photocatalytic activity [245]. As addition, GO has the potential to enhance the optical and structural properties of the composite, increasing the specific surface area and pore volume while significantly suppressing the recombination of photogenerated electron–hole pairs, ultimately resulting in superior photocatalytic performance. On the other hand, PVDF has excellent chemical resistance, high thermal stability, and mechanical strength, and it is also low‐cost [246]. Therefore, these compounds can work well as substrates in multicomponent MOF‐based multimetallic composites. For instance, Bagherzadeh et al. [245]. synthesized a multimetallic composite (MIL‐101(Fe)/CoFe_2_O_4_/(3%)GO). The band gap value in the resulting composite was reduced from 2.5 to 2.29 eV compared to MIL‐101(Fe). The charge carrier recombination rate and charge‐transfer resistance in the final structure were also reduced. As a result, this improvement in catalytic activity is due to the synergistic effect of integrating the MOF with graphene and cobalt ferrite particles. Compared to the initial components, the resulting composite showed the best performance for visible light photocatalytic degradation and photo‐Fenton‐like degradation of Direct Red 23 (DtR‐23) dyes, Reactive Red 198 (ReR‐198) dyes, and also the antibiotic tetracycline hydrochloride (TC‐H). So, the degradation of the dye using the composite reached more than 99% after 70 min of visible light irradiation. In another study, Chen et al. showed that a multimetallic CoFe‐MOF/TiO_2_/PVDF composite showed excellent performance for photo‐Fenton‐like degradation of TC, reaching an efficiency of 92.9%. This efficiency can be attributed to the hydrophilicity and optimization of the membrane porosity achieved by combining TiO_2_ and the CoFe‐MOF. The results also indicate that the incorporation of the CoFe‐MOF and TiO_2_ facilitates the separation of photoexcited electrons and holes and helps them to be transported more easily in the structure [247]. In 2024, Rabeie et al. [248]. reported the synthesis of a multimetallic composite containing iron. The starch/MIL100/CoFe_2_O_4_ composite was utilized for the photocatalytic degradation of MB and TC. The results show that the performance of the composite has significantly improved from 12% to 96.6% for the degradation of MB and from 18% to 91.2% for the degradation of TC compared to that of starch after 240 min (Figure 7). This significant improvement in photocatalytic activity is related to the presence of magnetic cobalt ferrite particles and MIL‐100 due to the high production of active species and electron–hole pairs.

**FIGURE 8:**
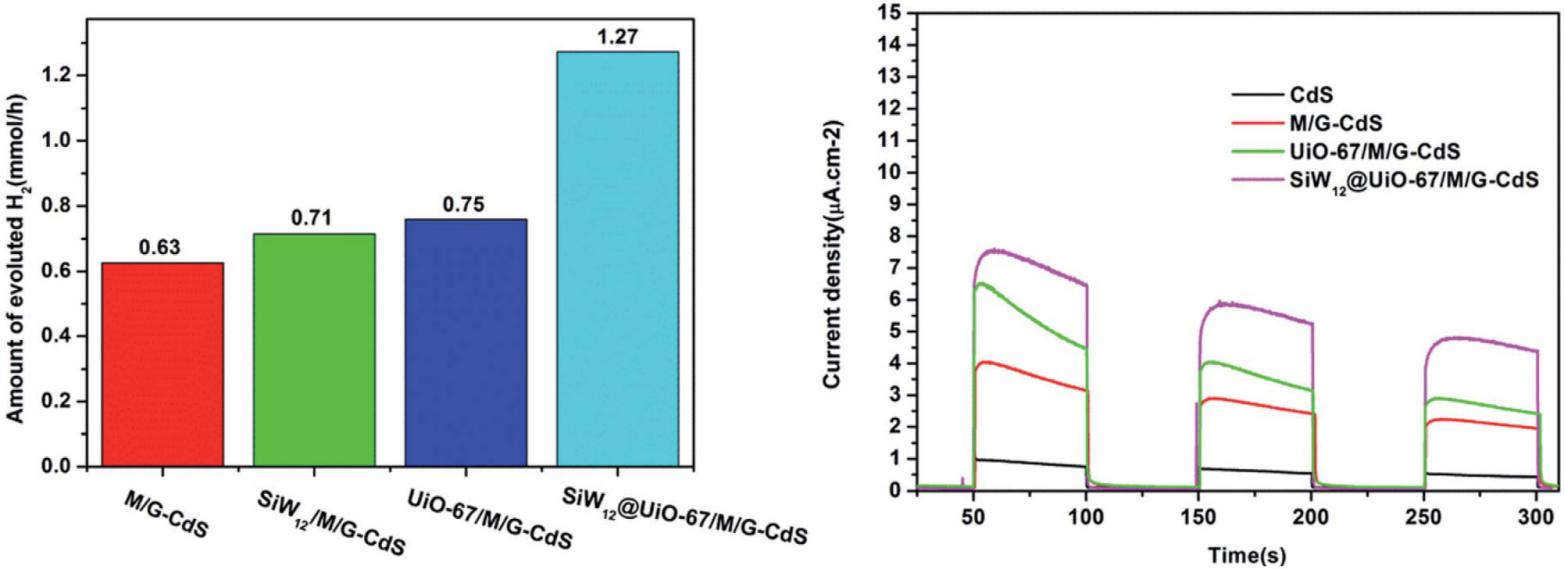
Hydrogen production process using various photocatalysts under visible light illumination and transient photocurrent response of various photocatalysts under visible light irradiation. Reproduced with permission [254]. Copyright 2016, Royal Society of Chemistry.

In addition to the use of iron‐based MOFs, ZnFe_2_O_4_ NPs with a suitable band gap (1.9 eV) are used in this specific photocatalytic application. For example, see Sharafinia et al. [249] synthesized the multimetallic composite g‐C_3_N_4_/ZnFe_2_O_4_/UiO‐66. The resulting composite showed a significant improvement in the degradation of RhB, achieving 98% degradation after 90 min. This performance improvement is due to the reduction of the band gap from 3.6 eV for the MOF to 2.8 eV for the composite. There is also a more effective separation of electrons and holes and an increase in the surface area of the composite. In addition, graphitic carbon nitride (g‐C_3_N_4_) has unique properties, such as physicochemical stability, high conductivity, and good conductivity in the visible‐light response. The employment of silver‐based photocatalysts has also been considered in photocatalytic degradation. For example, El‐Fawal et al. reported the synthesis of a multimetallic composite containing AgFeO_2_ and tested it for the degradation of pharmaceutical pollutants. The photocatalytic degradation efficiency of real pharmaceutical wastewater pollutants by the AgFeO_2_‐graphene/Cu_3_(BTC)_2_ composite reached ~ 91% in 7 h, which is much higher than that of the starting materials. This performance improvement can be attributed to the reduction of the band gap from 1.95 to 1.72 eV as well as the unique SPR of Ag NPs. The SPR properties of Ag NPs are very suitable for improving the photocatalytic ability through more efficient separation of charge carriers [250]. In an additional study, Akbarzadeha et al. utilized Ag_3_VO_4_ NPs in the synthesis of the Ag_3_VO_4_/Cu‐MOF/rGO (rGO denoted as reduced graphene oxide) composite, and the resulting composite showed excellent performance for photocatalytic dye removal [100]. Furthermore, UiO‐66 is commonly used in photocatalytic degradation. This MOF has abundant active sites and is also highly stable in water. Therefore, it is a suitable material for employment in photocatalytic degradation in aqueous environments. To cite an instance, Wang et al. synthesized the N‐K_2_Ti_4_O_9_/g‐C_3_N_4_/UiO‐66 composite using UiO‐66 and investigated it for the photocatalytic degradation of RhB. The resulting composite showed the highest degradation rate, which could be attributed to the efficient electron and hole transport generated in the composite, as well as the high adsorption activity of the composite due to the presence of UiO‐66 [244]. In another example, An et al. reported the synthesis of a ZnIn_2_S_4_/UiO‐66/calcined graphite felt (ZU/C‐GF) composite. The composite exhibited a photocatalytic degradation rate of about 97% for TC degradation after 120 min, which is approximately 5 times greater than that of ZnIn_2_S_4_/graphite felt and 3.76 times greater than that of ZnIn_2_S_4_/calcined graphite felt [251]. Also, in another study, the synthesis of a multimetallic composite α‐SnWO_4_/UiO‐66‐NH_2_/g‐C_3_N_4_ was reported by Wei et al. The resulting composite showed outstanding performance in removing Ibuprofen, reaching an efficiency of 95.5% in 2 h, while each component alone had a degradation rate of 20–40%. This improved performance is attributed to the synergistic effect of these three components in enhancing the absorption of Ibuprofen and the effective separation of photogenerated carriers [252]. Other composites that have been utilized for photocatalytic degradation include the CQDs/TiO_2_@Ti‐TPA‐MOF/Cu_0.5_Zn_0.5_S (CQDs: carbon quantum dots) composite, synthesized by Abbassian et al. This composite increased the photocatalytic degradation of gentamicin by 6 times compared to the Ti‐TPA‐MOF and reached a yield of 95% after 80 min. This increase in performance has been attributed to the narrow band gap (reduction from 3.63 eV for the MOF to 2.28 eV for the composite), increased active sites, and reduced electron–hole pair recombination due to the presence of CQDs and Cu_0.5_Zn_0.5_S nanorods [253]. Additionally, Hu et al. reported the synthesis of the Bi_2_WO_6_/MIL‐53(Al)/PVDF composite, which exhibited a high efficiency of 95.3% for the degradation of RhB after 60 min. The results showed that the combination of MOF and Bi_2_WO_6_ could reduce the recombination rate of electron–hole pairs and improve the photocatalytic activity [246]. Other applications of these MOF‐based multimetallic composites include H_2_ production [252, 254, 255, 256]. Bu et al. reported the synthesis of the SiW_12_@UiO‐67/M/G–CdS multimetallic composite. As shown in Figure 8a, this composite exhibited excellent H_2_ production performance compared to that of its constituent components. This excellent photocatalytic performance can be attributed to the large specific surface area of SiW_12_@UiO‐67, which significantly enhances its reactivity. Furthermore, the incorporation of SiW_12_ into UiO‐67 not only facilitates electron transport in the composite structure but also contributes to the effective separation of electron–hole pairs by suppressing the fast recombination of CdS charge carriers (Figure 8b). In addition, the composite shows a significant reduction in the band gap energy, which decreases from 3.5 eV in SiW_12_@UiO‐67 to 2.3 eV in the final composite. This significant reduction is attributed to the synergistic presence of CdS NPs and MoS_2_/rGO (M/G), which collectively enhance the optical and catalytic properties of the composite [254]. Also, for hydrogen production in the NH_2_‐MIL‐125(Ti)/g‐C_3_N_4_/NiPd composite synthesized by Xu et al., 322‐fold improvement in performance over NH_2_‐MIL‐125(Ti)/CN and a 1.3‐fold improvement over NH_2_‐MIL‐125(Ti)/NiPd have been achieved with an outstanding performance of 8.7 mmol g^−1^ h^−1^. This improvement can be attributed to the close interfacial contact between NH_2_‐MIL‐125(Ti) and CN, which ultimately accelerates charge transfer. Also, the presence of NiPd NPs improves the charge transfer and increases the light absorption capacity of NH_2_‐MIL‐125(Ti)/CN [255].

In terms of MOF/metal‐based partner/carbon‐based partner heterojunctions, for example, systems containing MIL‐101(Fe)/CoFe_2_O_4_/GO [245] commonly operate through this mechanism. In the Z‐scheme heterojunction, electrons remain in the CB of MIL‐101(Fe), while holes accumulate in the VB of CoFe_2_O_4_, creating strong charge separation. Although CoFe_2_O_4_ cannot generate ^**.**^OH directly from water, the CB potential of MIL‐101(Fe) is sufficiently negative to produce ^**.**^OH through the H_2_O_2_/^**.**^OH pathway, and the Fe^3+^/Fe^2+^ cycle further enhances hydroxyl‐radical formation through photo‐Fenton reactions. The key distinction between the Z‐scheme and the type‐II mechanism is the much higher production of superoxide radicals. In the Z‐scheme, ^**.**^O_2_^−^ can form both by direct reduction of O_2_ at the CB of MIL‐101(Fe) and by oxidation of H_2_O_2_ at the VB of CoFe_2_O_4_, whereas type‐II systems rely only on the weaker H_2_O_2_ pathway. Because ^**.**^O_2_^−^ plays a major role in pollutant degradation, the Z‐scheme pathway is considered the dominant mechanism in this system. The Z‐scheme charge–transfer pathway has been widely observed. Numerous composites such as CoFe‐MOF/TiO_2_/PVDF [247], α‐SnWO_4_/UiO‐66‐NH_2_/g‐C_3_N_4_ [252], AgFeO_2_/G@Cu_3_(BTC)_2_ [250], g‐C_3_N_4_/Ti_3_C_2_/Sn‐Bi‐MOF [257], and BiVO_4_/MOF−808/CN/CQD [258] have been shown to operate through this mechanism, consistently exhibiting efficient charge separation and strong redox capability. These examples reinforce the tendency of mixed‐component MOF systems to favor Z‐scheme behavior over the traditional type‐II mechanism. Furthermore, a representative example is the S‐scheme heterojunction formed between UiO‐66 and ZIS. When these two materials come into contact, electrons naturally migrate from ZIS to UiO‐66 until their Fermi levels align, creating surface charge accumulation and an IEF. This built‐in field bends the energy bands and directs photogenerated electrons from UiO‐66 toward ZIS, where they recombine with low‐energy holes. As a result, the system retains the highly reducing electrons in the CB of ZIS and the strongly oxidizing holes in the HOMO of UiO‐66, preventing recombination and enhancing the formation of reactive radicals. Such S‐scheme behavior has also been reported in other MMOF‐based composites, demonstrating its effectiveness in boosting photocatalytic degradation [251].

In the case of heterojunctions, most of them favor Z‐scheme or S‐scheme charge–transfer mechanisms, as these mechanisms enable efficient separation of photogenerated electrons and holes while preserving strong redox potentials. These mechanisms enhance the formation of reactive species such as ^**.**^O_2_^−^ and ^**.**^OH, boosting photocatalytic performance in applications like pollutant degradation, H_2_ production, and CO_2_ reduction. Only a few exceptional composites follow the traditional type‐II mechanism. Overall, the prevalence of Z‐ and S‐scheme pathways highlights the importance of band‐engineered heterojunctions in designing highly efficient MOF‐based photocatalysts.

### 3.3 Summary of Section

Table 2 summarizes the reported triple‐heterojunction MMOF composites and provides an overview of the current research in this area. As can be seen from this table, the use of UiO‐66 for this type of composite is more common. Among the advantages that have made this MOF popular over other MOFs are its high‐surface area and exceptional thermal stability. In addition, it shows good structural stability in aqueous, acidic, and alkaline environments [236, 240, 261]. In terms of charge–transfer mechanisms, most reported triple‐heterojunction MMOF composites have been interpreted as operating through Z‐scheme or S‐scheme pathways as these models provide a reasonable explanation for the combination of efficient charge separation and strong redox capability observed in many studies. These mechanistic assignments are commonly inferred from characterization techniques such as band structure analysis, radical trapping experiments, ESR spectroscopy, and photoelectrochemical measurements. However, it should be noted that such evidence is often indirect and may not unambiguously distinguish Z‐scheme or S‐scheme pathways from other charge–transfer mechanisms. Therefore, complementary in situ/operando characterization techniques together with theoretical calculations remain important for rigorous mechanistic verification. Only a limited number of reported composites have been interpreted as following the conventional type‐II mechanism. Overall, the widespread use of Z‐scheme and S‐scheme models highlights the importance of band‐engineered heterojunctions as a valuable design strategy for MMOF‐based photocatalysts.

**TABLE 2:** Summary of the photocatalytic performance in MMOF composites containing triple heterojunctions.

Name	Metals	Band gap (eV)	Application	Performance	References
Bi_*x*_Ti_*y*_O_z_/MOF‐In_2_S_3_/CdIn_2_S_4_	Bi/Ti/In/Cd	—	Degradation	64.1% TC	[224]
ZnO nanorods/Bi_2_MoO_6_/MIL‐101(Fe)	Zn/Bi/Mo/Fe	2.3	Degradation	96.1% TC	[233]
Ti‐MOF/Ag/NiFe‐LDH	Ti/Ag/Ni/Fe	2.09	Degradation	95% RhB/92% LVE	[241]
ZIF‐67@CoWO_4_@CoS	Co/W	2.67	Degradation	100% MB	[235]
PCN‐222‐PW_12_/TiO_2_	Mn/Zr/W/Ti	2.41	Degradation	98.5% RhB/94.83% OFL	[243]
Ag_3_PO_4_/MIL‐101/NiFe_2_O_4_	Ag/Cr/Ni/Fe	2.24	Degradation	93% RhB	[230]
ZnIn_2_S_4_/UiO‐66/TiO_2_/Ti	Zn/In/Zr/Ti	2.3	Degradation	98.01% RhB	[225]
BiOI/ZnFe_2_O_4_/MIL‐88B(Fe)	Bi/Zn/Fe	—	Degradation	73% AB92/65.47% MB/59.52% phenol	[234]
La‐Bi_2_WO_6_‐BiFeWO_*x*_‐WO_3_@MOF	La/Bi/W/Fe	1.86	Degradation	98.62% phenol/92.41% nicotine/81.74% benzopyrene/98.23% nitrosamines	[259]
Ag_3_PO_4_/Bi_2_S_3_‐HKUST‐1‐MOF	Ag/Bi/Cu	2.07	Degradation	98.44% TB/99.36% VS	[231]
AgFeO_2_‐/G@Cu_3_(BTC)_2_	Ag/Fe/Cu	1.72	Degradation	92.9% AMC/91.4% DCF	[250]
ZnIn_2_S_4_/UiO‐66/calcined graphite felt	Zn/In/Zr	1.57	Degradation	97% TC	[251]
CQDs/TiO_2_@Ti‐TPA‐MOF/Cu_0.5_Zn_0.5_S	Ti/Cu/Zn	2.28	Degradation	95% Gentamicin	[253]
Starch/MIL‐100/CoFe_2_O_4_	Co/Fe	2.7	Degradation	96.6% MB/91.2% TC	[248]
MIL‐101(Fe)/CoFe_2_O_4_/GO	Fe/Co	2.29	Degradation	99.93% direct red 23/99.65% reactive red/2% TC	[245]
g‐C_3_N_4_/ZnFe_2_O_4_/UiO‐66	Zn/Fe/Zr	2.8	Degradation	98% RhB	[249]
Bi_2_WO_6_/MIL‐53(Al)/PVDF	Bi/W/Al	—	Degradation	95.3% RhB/76.8% MB/77.2% MG	[246]
α‐SnWO_4_/UiO‐66‐NH_2_/g‐C_3_N_4_	Sn/W/Zr	2.48	H_2_ production/Degradation	95.5% IPF/H_2_: 2105 μmol g^−1^ h^−1^	[252]
NH_2_‐MIL‐125(Ti)/Ti_3_C_2_ QDs/ZnIn_2_S_4_	Ti/Zn/In	2.16	H_2_ production/Degradation	96% TC/98% SMZ/H_2_: 2931.9 μmol g^−1^ h^−1^	[227]
UiO‐66–NH_2_/CdIn_2_S_4_/CaIn_2_S_4_	Zr/Cd/In/Ca	—	H_2_ production/Degradation	4931 μmol g^−1^ h^−1^/ASM > 93%	[226]
MOF‐5/CuO@ZnIn_2_S_4_	Zn/Cu/In	—	H_2_ production/Degradation	1938.3 μmol g^−1^ h^−1^/98.7% 2,4‐DCP/97.3% phenol	[228]
UiO‐66‐(COOH)_2_/MoS_2_/ZnIn_2_S_4_	Zr/Mo/Zn/In	2.52	H_2_ production/Cr(VI) reduction	H_2_ production: 18.794 mmol g^−1^ h^−1^/Cr(VI) reduction: 0.06850 min^−1^	[240]
TiO_2_/Ti_3_C_2_–TiC/Ce/Zr‐UiO‐66‐NH_2_	Ti/Ce/Zr	—	H_2_O_2_ and H_2_ production	H_2_O_2_ 1575 μmol g^−1^ h^−1^/H_2_ 570 μmol h^−1^	[238]
Co‐MOF/MXene/BiVO_4_	Co/Ti/Bi/V	2.53	Photoelectrochemical Water Splitting	Photocurrent: 4.26 mA/cm^2^	[260]
NiCo_2_O_4_@MOF‐801@MIL‐88A	Ni/Co/Zr/Fe	2.55	Degradation/Cr(VI) reduction	85% meropenem/100%	[232]
SiW_12_@UiO‐67/M/G–CdS	Si/W/Zr/Mo/Cd	2.3	H_2_ production	1.27 mmol h^−1^	[254]
Ni_*x*_Mo_1‐x_S_2_/MOF‐5@g‐C_3_N_4_	Ni/Mo/Zn	—	H_2_ production	319 μmol in 5h	[256]
NH_2_‐MIL‐125(Ti)/g‐C_3_N_4_/NiPd	Ti/Ni/Pd	—	H_2_ production	8.7 μmol g^−1^ h^−1^	[255]
NH_2_‐UiO‐66/CoFe_2_O_4_/CdIn_2_S_4_	Zr/Co/Fe/Cd/In	1.97	H_2_ production	283.26 μmol h^−1^	[261]
PdS@UiOS@CZS	Pd/Zr/Cd/Zn	2.59	H_2_ production	460.8 μmol h^−1^	[239]
Au@UiOS@ZIS	Au/Zr/Zn/In	2.59	H_2_ production	391.6 μmol h^−1^	[118]
Ni_2_P@UiO‐66‐NH_2_/Zn_0.5_Cd_0.5_S	Ni.Zr/Zn/Cd	—	H_2_ production	40.91 mmol g^–1^ h^–1^	[236]
Na_*x*_‐C_3_N_4_/Pt@UiO‐66	Na/Pt/Zr	—	H_2_ production	471.4 μmol g^−1^ h^−1^	[237]
BiVO_4_/MOF−808/CN/CQD	Bi/V/Zr	2.33	Oxidative desulfurization	99.5% DBT	[258]
CoFe‐MOF/TiO_2_/PVDF	Co/Fe/Ti	—	Removal	92.9% TC	[247]
g‐C_3_N_4_/Ti_3_C_2_/Sn‐Bi‐MOF	Ti/Sn/Bi	2.5	CO_2_ reduction	CO yield: 36.33 μmol g^−1^ h^−1^	[257]
CdS‐P25/ZIF‐67	Cd/Ti/Cu	1.89	CO_2_ reduction	Photocurrent: 3.47 μA cm^−2^	[242]
BiVO_4_@Au@UiO‐66‐NH_2_	Bi/V/Au/Zr	—	CO_2_ reduction	CO production: 232.7 μmol g^−1^ h^−1^	[262]

Abbreviations: AB92, acid blue 92; AMC, amoxicillin; ASM, asulam; DBT, dibenzothiophene; DCF, diclofenac; IPF, ibuprofen; LVE, levofloxacin; TB, trypan blue; VS, vesuvin.

## 4 MMOFs/Single‐Metallic Partner Composites

**FIGURE 9:**
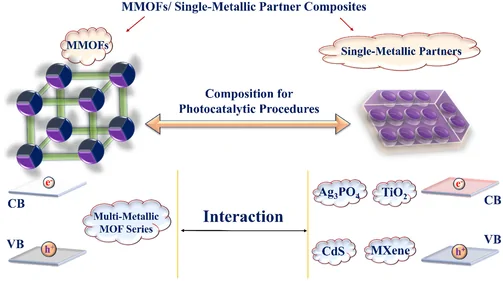
Representation of MMOFs/single‐metallic partner composites as photocatalysts.

Doping metal ions into MOF structures provides more active sites for the occurrence of photocatalytic reactions. MMCT between different metal ions can enhance the charge transfer and enhance the photocatalytic efficiency. Transition‐metal ions are usually introduced into the structure of MOFs to improve the catalytic performance because the d–d transitions in them lie in the visible light range [263]. Based on the data in Table 3, most of the reported composites have been used in the photocatalytic degradation of pollutants, and a smaller number of other applications such as carbon dioxide reduction and bacterial destruction have also been reported. Ag_3_PO_4_ and TiO_2_ semiconductors are among the most widely used materials in the preparation of composites. Also, the schematic description of multimetallic‐organic rameworks/single‐metallic partner composites is exhibited in Figure 9.

**TABLE 3:** Summary of the photocatalytic performance for MMOF/single‐metallic partner composites.

Name	Metals	Band gap (eV)	Application	Performance	References
ZnO@ [Na_2_Zn_3_(btc)2(μ‐HCOO)_2_(μ‐H_2_O)_8_]*n*	Na/Zn	3.15	Degradation	97.53% MB	[264]
Ce@MIL‐101‐NH_2_/Ag_3_PO_4_	Ce/Cr/Ag	2.18	Degradation	MO, RhB, and TC of 73, 88, and 68%, respectively	[265]
CeO_2_@ MIL‐53(Fe/Co)	Ce/Fe/Co	—	Degradation	ATZ 99%	[40]
Cu/Zr‐MOF@CdS	Cu/Zr/Cd	2.37	CO_2_ methanation	52.2 μmol g^−1^ h^−1^	[29]
NH_2_‐MIL‐88B (Co/Fe)/Ag_3_PO_4_	Co/Fe/Ag	—	Degradation	76.2 % RhB	[266]
NH_2_‐MIL‐88B (Fe/Co)/Ag_3_PO_4_/(PPy)	Co/Fe/Ag	—	Degradation	90% RhB	[28]
ZnFe‐MOF/AgCl	Zn/Fe/Ag	2.87	Degradation	90.33% LVE	[50]
Co@MIL‐88B(Fe)/Al_2_O_3_	Co/Fe/Al	2.15	Degradation	90% phenol	[42]
TiO_2_@CoNi‐MOF	Ti/Co/Ni	—	CO_2_ reduction	41.65 μmol g^−1^ h^−1^	[49]
Sn–Bi–MOF/Ti_3_C_2_	Sn/Bi/Ti	—	Degradation	96.2% TC	[267]
TiO_2_/TP‐222(Zn)	Ti/Zr/Zn	—	Degradation	68% 2,4‐DNP	[32]

Abbreviation: ATZ, atrazine.

**FIGURE 10:**
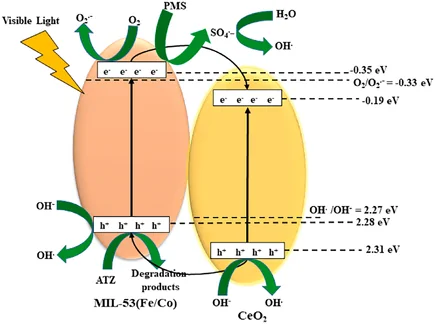
Heterojunction configuration between CeO_2_ and MIL‐53(Fe/Co). Reproduced with permission [40]. Copyright 2022, Elsevier.

In the combination of TiO_2_ NPs and MOF, a photoexcitation process is generated, but the undesirable recombination of photoexcited electronic processes during photocatalysis is reduced due to the newly formed structures [268]. In the work of Wang et al., the rate of CO_2_ reduction to CO reached 31.32 µmol g^−1^ h^−1^, which is about 7 times higher than that of mere TiO_2_. The enhanced photocatalytic efficiency after incorporation may be primarily due to the accelerated electron transfer. Furthermore, the dispersion of TiO_2_ particles in the CuTCPP@UiO‐66 MOF leads to the creation of accessible reactive sites, resulting in enhanced CO_2_ adsorption compared to that of pristine TiO_2_ [269]. The enhanced light harvesting and the inhibitory agent against the recombination of charge carriers greatly enhance the photocatalytic process. In the work of Wang et al., two factors realize these two processes: the metalloporphyrin‐containing MOF sufficiently enhances the light absorption rate, and the other is through the interaction between the MOF and TiO_2_ in the Z‐scheme mechanism, which facilitates the separation of photoexcited charges. They synthesized the composite copper(II)‐porphyrin zirconium MOF PCN‐224(Cu)/TiO_2_ as a photocatalyst to promote the CO_2_ reduction process under simulated solar light irradiation (λ > 300 nm). The rate of CO production in the absence of the cocatalyst and sacrifice reagent was 37.21 μmol g^−1^ h^−1^, about 10 and 45.4 times that of pristine PCN‐224(Cu) (3.72 μmol g^−1^ h^−1^) and mere TiO_2_ (0.82 μmol g^−1^ h^−1^), respectively [270]. Chen et al. enhanced the visible light absorption of the MOF by using the ligand 1‐(carboxymethyl)‐pyrazole‐4‐carboxylic acid and Fe doping in the preparation of the ZnFe‐MOF/AgCl‐x. In the highly conjugated system of the ligand, the transfer of π electrons is facilitated, resulting in enhanced light absorption. During the photocatalytic process, AgCl gradually decomposes, and Ag NPs are formed. The silver NPs act as a bridge during the photocatalytic process and accelerate the charge transfer in the Z‐pattern heterogeneity. Charge transfer between Zn^2+^ and Fe^2+^ and unsaturated metal sites in MOFs as catalytically active centers by promoting the adsorption and reaction of substrate molecules enhance the performance of the photocatalytic process. The photocatalytic degradation performance of the LVF pollutant was investigated. The ZnFe‐MOF/AgCl‐50 material can degrade 90.33% of LVE in 21 min [50]. Porphyrin MOFs, which contain metal‐porphyrin linkages, have several porphyrin‐attributed functions in catalysis, such as light harvesting and oxygen conversion [271]. Zhang et al. used a porph‐MOF, PCN‐222(Zn), which consists of zirconium clusters connected by the zinc‐tetrakis(4‐carboxyphenyl)porphyrin (ZnTCPP) ligand, for obtaining the TiO_2_ NPs and PCN‐222(Zn) composite photocatalysts (TP‐222(Zn)). TiO_2_ NPs are linked to 4‐mercaptopyridine (4‐PySH) via the thiol group and are also coordinated to ZnTCPP in PCN‐222 (Zn) via pyridine. PCN‐222(Zn) has advantages such as a high surface area, large open channels, high stability, and absorption range of almost the entire visible light region (with a band gap energy of 1.74 eV). TiO_2_ NPs act as electron acceptors from MOFs, effectively promoting separation and optical charge transfer, thereby prolonging the electron lifetime. The photocatalytic efficiency for the degradation of RhB and 2,4‐dinitrophenol (2,4‐DNP) was carried out under visible light irradiation by the TP‐222(Zn) composite and was significantly enhanced (degradation of 50 mg L ^−1^ of RhB solution using a 2.4 mg catalyst in 270 min) [32]. Furthermore, NPs with a high surface area exhibit good semiconducting properties. MOFs, with their porosity, can host NPs, and the fast recombination (e^−^/h^+^) between the MOF structures and the metal NPs can reduce the band gap [272]. Two metals, with similar ionic radii and coordination arrangement, can form stronger synergistic interactions compared to metals in different SBUs. Roy et al. [40]. prepared a heterogeneous photocatalyst by growing the bimetallic MOF MIL‐53 (Fe/Co) on CeO_2_ NPs dispersed in an aqueous medium and investigated its performance for the activation of peroxymonosulfate (PMS) in water under visible light irradiation. The catalyst/PMS system was employed to degrade ATZ in an aqueous medium. The continuous heterogeneous bonding between CeO_2_ and MIL‐53 (Fe/Co) reduces the e^−^/h^+^ recombination, increases the carrier migration rate, and improves the photocatalytic activity. Based on the UV–Vis DRS study, Eg of CeO_2_ and MIL‐53(Fe/Co) were achieved as 2.31 and 2.63 eV, respectively. CeO_2_ NPs with high photosensitivity and suitable charge mobility in the MOF framework enhance the visible light‐harvesting capability. The ECB of MIL‐53 (Fe/Co) (−0.35 eV) is higher than that of CeO_2_ (−0.19 eV); therefore, the e^−^s are transferred from the CB of MIL‐53(Fe/Co) to the CB of CeO_2_ upon visible light irradiation. On the other hand, the photoexcited h^+^s are transferred from the VB of CeO_2_ to that of MIL‐53(Fe/Co) because the VB of CeO_2_ (2.31 eV) is higher than the VB of MIL‐53(Fe/Co) (2.28 eV). In this way, the rate of recombination of the excited charge carriers decreases (Figure 10). With the intervention of PMS as an oxidant, the composite photocatalyst produced both radical (SO_4_^⋅−^, OH^⋅^ and O_2_^⋅−^) and nonradical ($O_{2}^{1}$ and h^+^) species that mineralized ATZ molecules under visible light irradiation. The catalytic reaction rate is more affected by surface‐bound reactions than by the adsorption of reactant molecules.

**FIGURE 11:**
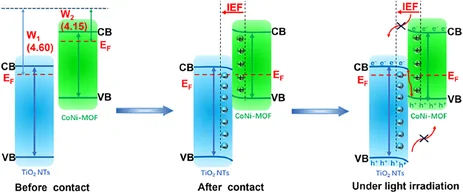
S‐scheme charge transfer route between TiO_2_ and CoNi‐MOF. Reproduced with permission [49]. Copyright 2023, Elsevier.

**FIGURE 12:**
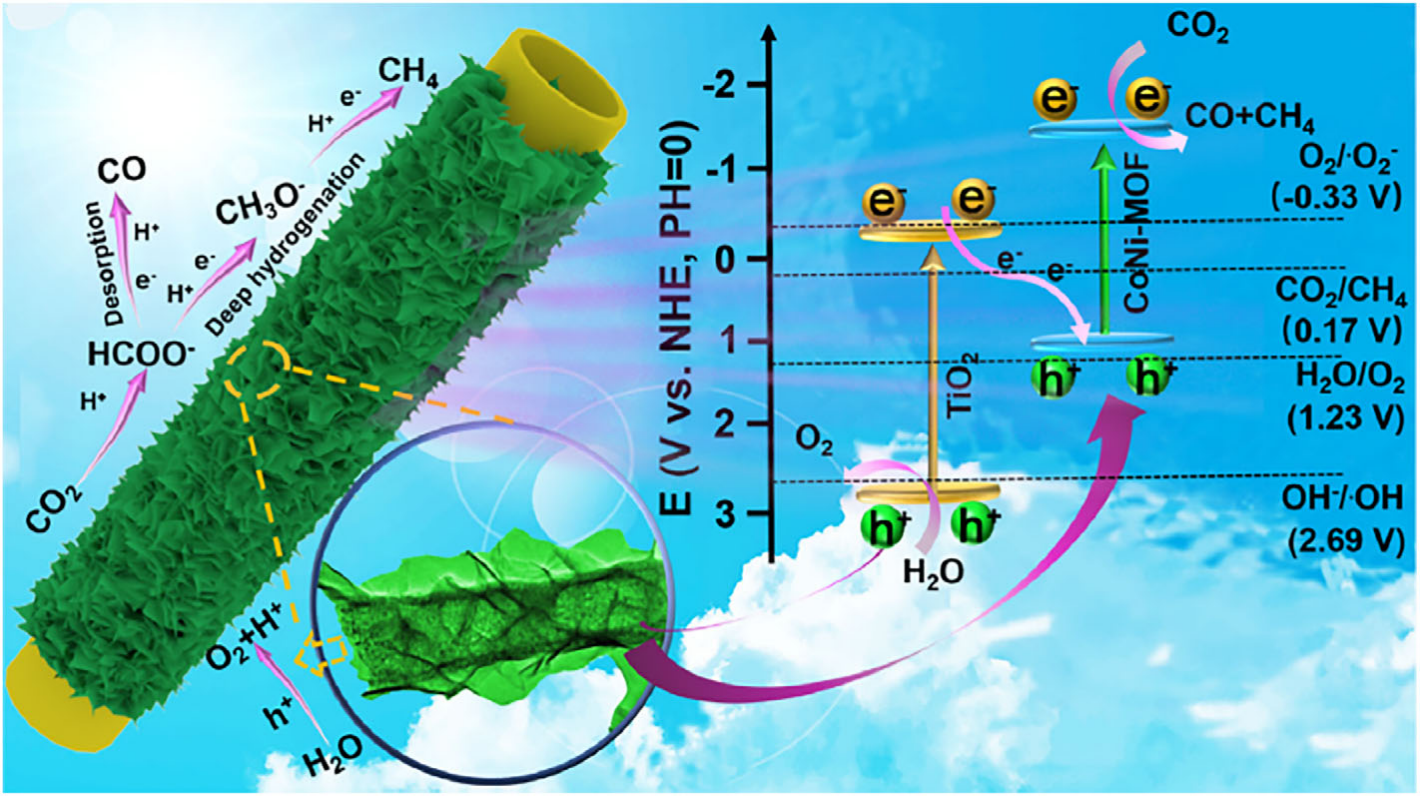
Photocatalytic CO_2_ reduction process over TiO_2_@CoNi‐MOF NTs. Reproduced with permission [49]. Copyright 2023, Elsevier.

Liang et al. [49]. synthesized a hierarchical S‐scheme using TiO_2_@CoNi‐MOF based on heterogeneous nanotubes (NTs) heterogeneous photocatalyst. In the TiO_2_@CoNi‐MOF heterogeneous junction, under the influence of light irradiation, electrons generated from the TiO_2_ and CoNi‐MOF VBs are transferred to their respective CBs. The synergy of Coulomb interaction, internal electric field, and interface band bending leads to the transfer of electrons from CB TiO_2_ to the VB CoNi‐MOF. Therefore, these electrons can recombine with holes within the VB CoNi‐MOF (Figure 11). According to the results, the photoinduced charge transfer path of TiO_2_@CoNi‐MOF NTs is consistent with the S‐scheme mechanism (Figures 11 and 12). COO^−^ species trap protons and produce ^.^COOH radicals, leading to the formation of H_2_O and CO. Most of the COO^−^ intermediate species react with protons to form CHO* and CH_3_O* intermediates, resulting in the formation of CH_4_ by dehydrogenation (Figure 12). The optimized TiO_2_@CoNi‐MOF NTs show significantly higher CO_2_ photoreduction performance than the single component samples (TiO_2_ and CoNi‐MOF), and CH_4_ yield is the amount of 41.65 µmol g^−1^ h^−1^ with a 93.2% selectivity.

**FIGURE 13:**
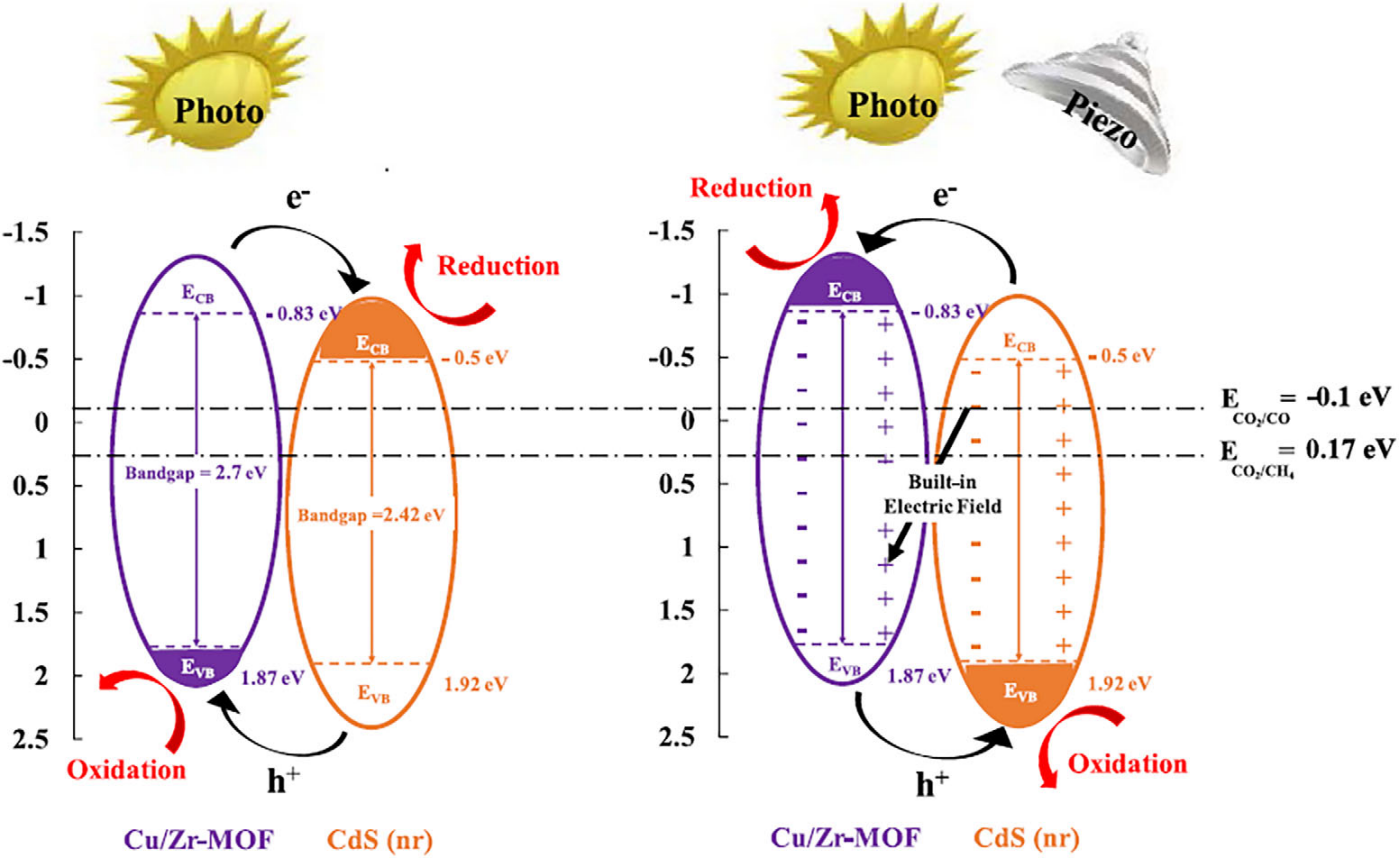
Possible piezo‐photo catalyst mechanism of Cu/Zr‐MOF@CdS for charge transfer route. Reproduced with permission [29]. Copyright 2024, Elsevier.

**FIGURE 14:**
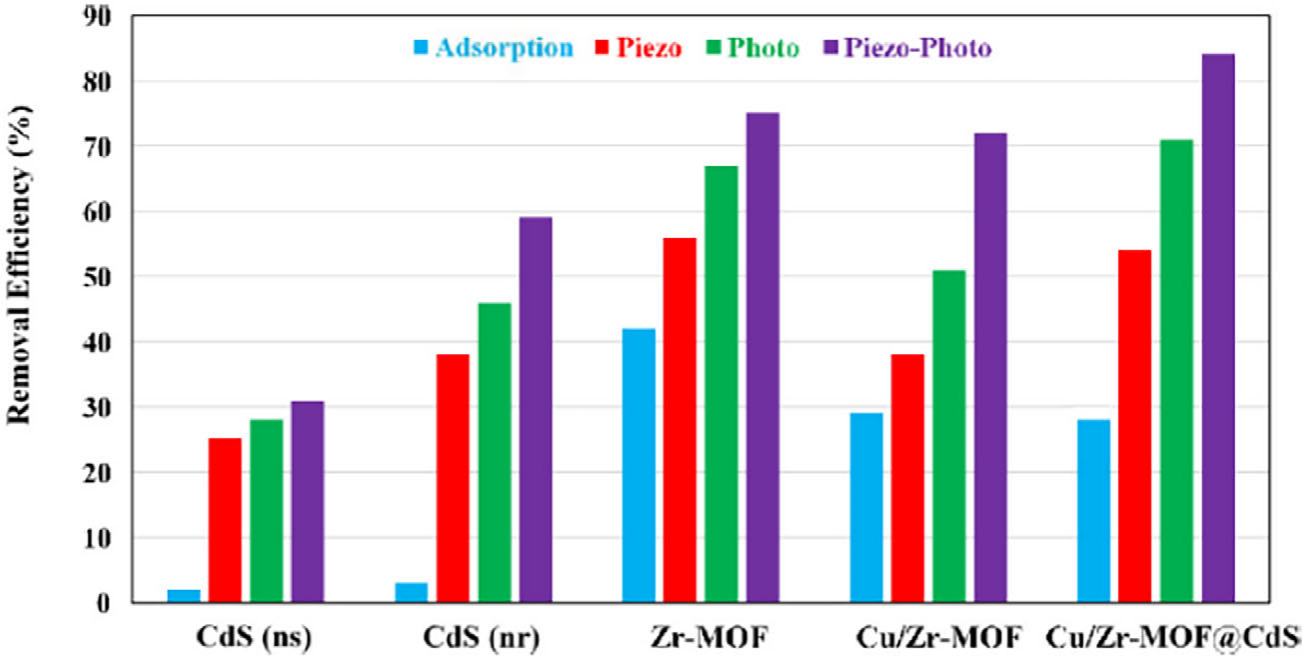
Dye degradation results of different catalysts under photo, piezo, and photo‐piezo processes. Reproduced with permission [29]. Copyright 2024, Elsevier.

NH_2_‐MIL‐125 loaded with Ru NPs was synthesized and combined with MnO_*x*_ to prepare the Ru@MIL‐125/MnO_*x*_ composite catalyst by Wang et al. [273]. The photocatalytic nitrogen fixation activity of this composite is about 10 times higher than that of untreated NH_2_‐MIL‐125. Ru@MIL‐125/MnO_*x*_ also shows higher OER activity after the introduction of MnO_*x*_. According to the results, MnO_*x*_ can act as an active center of the OER and can absorb holes. Ru is an electron absorber and is the site of the photocatalytic nitrogen reduction reaction. Therefore, the electron and hole recombination is effectively reduced by transferring the electrons and holes generated by light to Ru and MnO_*x*_, respectively. Furthermore, piezoelectric materials can be used in the field of photocatalysis due to their ability to convert mechanical energy into electrical energy and vice versa. Piezoelectric materials can improve the efficiency of the photocatalytic process by separating the charge and facilitating the migration of photocarriers [269]. Liang et al. used cadmium sulfide to impart additional piezoelectric properties and copper in the synthetic Cu/Zr‐MOF@CdS composite to enhance CH_4_ selectivity and CO_2_ conversion. One way to reduce greenhouse gases is through CO_2_ methanation; however, we face practical challenges in converting CO_2_ to methane due to thermodynamic factors due to the lower energy state of CH_4_ compared to CO_2_. The reduction of CO_2_ to CO requires two photogenerated electrons, while for CH_4_ production, eight electrons and eight protons are required. Therefore, CH_4_ production occurs in a kinetically unfavorable process with multistep proton/electron transfer [274]. Therefore, the development of high‐performance photocatalysts for the selective and efficient reduction of carbon dioxide to methane is of great importance. The photocatalytic process is one of the most suitable methods for CO_2_ conversion [49]. Both CdS and Zr‐MOF impart piezoelectric properties to the catalyst such that by integrating them, the process mechanism is transformed from a type‐II scheme to a Z‐scheme. As shown in Figure 13, during the photocatalytic process, the CB surface of CdS is reduced, and oxidation occurs at the VB of Cu/Zr‐MOF. Under the piezo‐photo process, electrons are transferred from CdS to Cu/Zr‐MOF, which leads to reduced recombination and increased charge separation efficiency [29]. Also, according to the report of Makdee et al., the presence of copper in the Zr‐MOF network controls the band gap of the nanorod composite, leading to lower CO production and higher CH_4_ selectivity (from 31% to 41%) [275]. The reduction occurred in the CB of CdS, which has a lower potential than the CB in the MOF, and the oxidation reaction occurred in the VB of the Cu/Zr‐MOF, which has a higher potential than the VB of CdS. In addition, by adding piezoelectric properties to the photocatalytic process, a strong internal electric field was generated in the composite framework. This phenomenon led to the formation of a new electron‐transport pathway and a heterogeneous bonding scheme. Finally, in the Piezo‐photo process, 99% conversion of CO_2_ was observed along with 35.5% selectivity of CH_4_, which was significantly higher than that of the separate photo process. The photocatalytic performance for dye degradation was investigated (Figure 14). These investigations were carried out under three different conditions, piezo, photo, and piezo‐photo processes. According to the results, the photo degradation process is more effective than the piezo process. Interestingly, CdS(nr) has much better piezo and photocatalytic properties compared to CdS(ns) [29]. This feature is due to the morphology and high crystallinity of the materials, which cause the generation of an internal electric field. However, the Cu/Zr‐MOF@CdS nanorod composite and CdS(nr) showed higher dye degradation under piezo, photo, and piezo‐photo processes [276]. The photo, piezo, and piezo‐photo catalytic performances of various catalysts including CdS, Zr‐MOF, Cu/Zr‐MOF, and Cu/Zr‐MOF@CdS were investigated in CO_2_ methanation. The photocatalytic performances of all catalysts were higher than their piezocatalytic efficiencies. Furthermore, the results showed that CdS did not contribute to CH_4_ production in the photocatalytic and piezo catalytic reactions, and only partial CH_4_ production was achieved through the photocatalyticalytic reaction, which resulted from the synergistic effect of the integration of the piezo and photo processes. As mentioned before, piezoelectric energy brought about a built‐in electricity field leading to the bending of the ECB level; this phenomenon also would ameliorate the CO_2_ conversion harshly. The Cu/Zr‐MOF@CdS composite showed 99% CO_2_ conversion and 35% selectivity for CH_4_ production in the piezo‐photo process, while 80% was observed for the CO_2_ photoconversion process alone [29].

Ag_3_PO_4_ is one of the proper semiconductors to prepare the MMOF composite because of its photocatalytic ability in the visible light range (with a band gap of around 2.4 eV) to produce^**.**^OH. Also, combining other materials with Ag_3_PO_4_ prevents undesirable effects such as optical reduction of silver and charge recombination [277]. Lee et al. used Co/Fe NH_2_‐MIL‐88B (Co/Fe) in the composite (referred to as MILx) and the associated heterogeneous bond with Ag_3_PO_4_ (referred to as Ag/MILx) to perform the adsorption reaction for photochemical reactions and the pollutant adsorption reaction RhB in water. The removal of RhB by the combined effect of adsorption and photocatalytic degradation was evaluated by the photocatalytic reaction. At a Co/Fe molar ratio of 0.2 in the heterogeneous cocatalyst, the RhB adsorption performance was increased by 28%, and the RhB degradation was improved by 1.5 times compared to the composite formed by Ag_3_PO_4_ and NH_2_‐MIL‐8 monometallic [266]. Lee et al. in the other work applied the composite NH_2_‐MIL‐88B (Fe/Co)/Ag_3_PO_4_ for wastewater treatment through the degradation of dyes and organic pollutants. The photocatalytic method is increasingly important due to the lack of secondary pollutants. They synthesized this photocatalytic composite by heterojunction formation of NH_2_‐MIL‐88B (Fe/Co) (n‐type) and Ag_3_PO_4_ (p‐type) and designed the optimal photocatalytic reaction for RhB removal from water by increasing the electrical conductivity to facilitate charge transfer in the heterojunction. Conductive Z‐pattern heterophotocatalysis by implementing polypyrrole (PPy) at the interface between Ag_3_PO_4_ and the MIL has accelerated the photocatalytic process by increasing light absorption and conductivity and generating reactive species. The removal of RhB was mainly carried out by the adsorption process for 180 min. Therefore, MIL alone lacks a photocatalytic reaction. The composite Ag/PPy/MIL@f decomposed RhB about 4 times faster than Ag/MIL@f, where about 90% of RhB was removed within 60 min [28]. Morais et al. used the photocatalytic advantages of Ag_3_PO_4_ to synthesize the MMOF composite Ag_3_PO_4_/NiCo‐MOF to evaluate bactericidal properties against *Staphylococcus aureus* (*S. aureus*) and *Escherichia coli* (*E coli*) bacteria with and without exposure to light emitted by white LEDs (WLED). When incubated with Ag_3_PO_4_ under WLED irradiation, a decrease in the minimum bactericidal concentration (MBC) was observed to a value of 64 μg/mL. The Ag_3_PO_4_/NiCoMOF‐5wt% composite, incubated without light, showed MBC values of 64 μg/mL for *S. aureus* and 32 μg/mL for *E. coli*, respectively. According to the results, the composite in the absence of WLED showed superior bactericidal results [28]. Advanced radical‐assisted oxidation of sulfate (SR‐AOPs) has been of interest due to its higher oxidative potential (2.5–3.1 V vs. 1.8–2.7 V ⋅OH) and longer half‐life (30–40 μs vs. 20 ns OH). Most often, sulfate radicals are generated through the activation of peroxydisulfate and PMS. On the one hand, cobalt is an effective dopant to suppress electron–hole recombination and enhance visible light absorption in the photocatalytic process. In addition, cobalt can activate persulfate (PS) to generate sulfate radicals with a high oxidation potential (*E*_0_ = 2.5–3.1 V) and improve the separation of free electron–hole pairs, which can enhance pollutant degradation. In the report of Hou et al., cobalt‐doped MIL‐88B(Fe) (xCoMIL‐88B(Fe)) was synthesized and grown on an Al_2_O_3_ support for wastewater treatment with a PMR system. In the PMR system, the 5CoMIL‐88B(Fe) membrane was used for phenol removal under light irradiation with and without the addition of the PMS. The radicals generated in the visible‐light‐activated MRS‐5CoMIL‐88B(Fe)@Al_2_O_3_ were able to remove phenol. Additional Co doping decomposed the structure and increased the BET surface area, leading to the preparation of metal sites and nonbonded organic bonds for phenol adsorption. The addition of PMS to the 5CoMIL‐88B(Fe)@Al_2_O_3_ membrane showed more than 90% phenol removal efficiency [42]. Furthermore, the formation of a Schottky junction is one way to facilitate electron transfer and charge separation between composite components. In the study of Cao et al. [267]., the photocatalytic ability of the Sn–Bi–MOF/Ti_3_C_2_ heterojunction with the Schottky junction was examined to degrade and mineralize TC under illumination. After 90 min of processes, a degradation efficiency of 96.2% and a mineralization rate of 45.5% were obtained. The decomposition efficiency of the TC solution was 9.9%, indicating that in the photo‐TC system, self‐photodegradation of TC molecules does not occur. The degradation efficiencies of the Ti_3_C_2_ and Sn–Bi–MOF catalysts for the TC solution after irradiation for 90 min were 69.1% and 79.7%, respectively. The photocatalytic performance of Sn–Bi–MOF/Ti_3_C_2_ was better than that of the Sn–Bi–MOF with doped Ti_3_C_2_. Sn–Bi MOF/Ti_3_C_2_ showed the best photocatalytic activity for the Ti_3_C_2_ doping of 0.05 g with a degradation efficiency of 96.2% in 90 min. However, when the doping amount was increased to 0.07 g, the photocatalytic performance decreased, which may be due to the agglomeration of Ti_3_C_2_ obstacle the formation of heterojunctions.

In summary, MMOFs/single‐metallic partner composites have received much less attention than other MMOF‐based composite systems. The incorporation of multiple metal ions into the MOF nodes can introduce additional active sites, modify the electronic structure, and tune the Fermi level. Combining these MMOFs with suitable semiconductor partners provides an effective strategy for developing photocatalysts for applications such as pollutant degradation, CO_2_ reduction, and H_2_ production. Many of the reported systems have been interpreted using proposed S‐scheme charge–transfer pathways to explain their enhanced photocatalytic performance. However, these mechanistic assignments are often based on indirect evidence and should be interpreted with caution. Further studies combining complementary characterization techniques with theoretical calculations will help provide a clearer understanding of the charge–transfer mechanism in these composites.

## 5 MMOFs/Multimetallic Partner Composites

**FIGURE 15:**
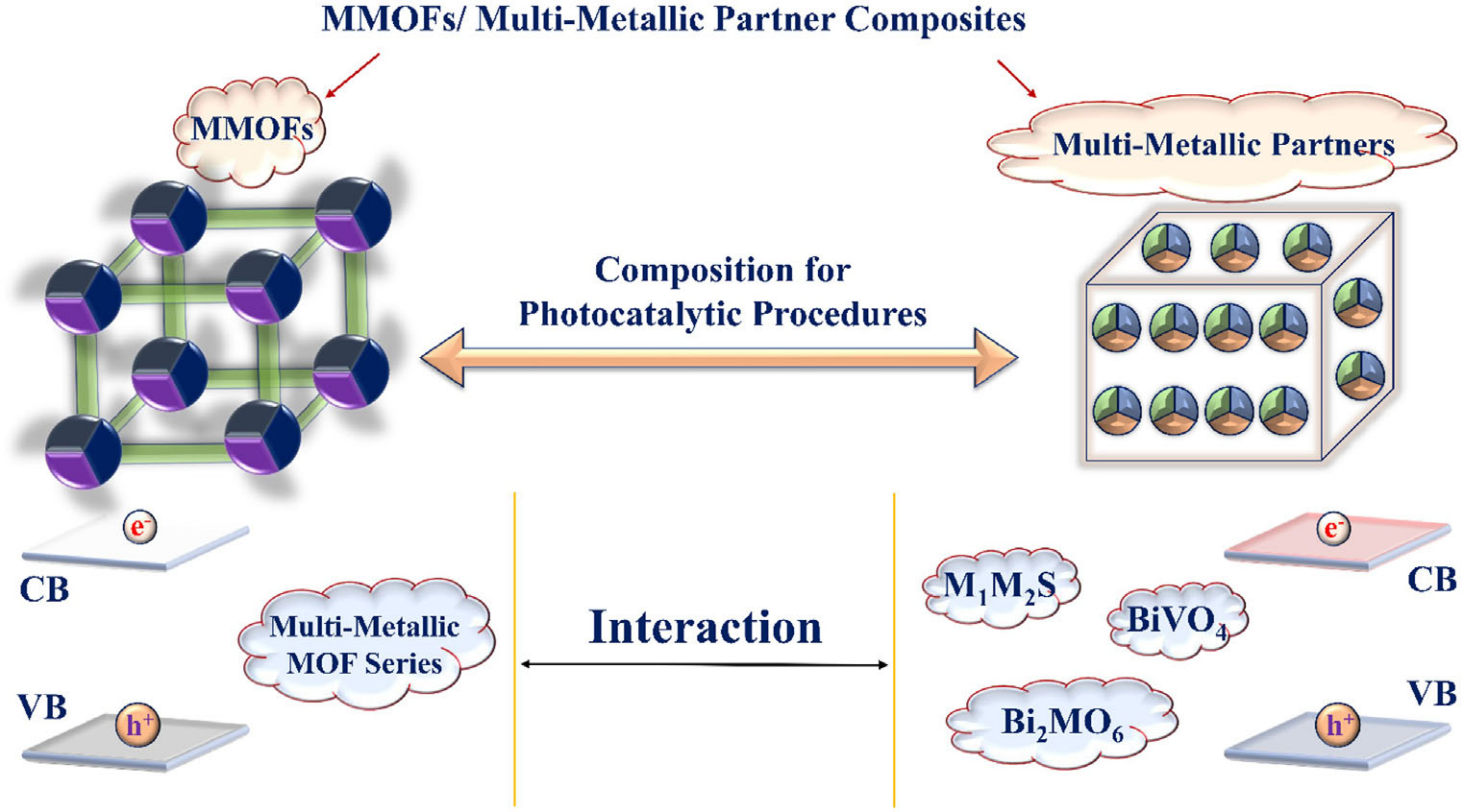
Schematic representation of MMOFs/multimetallic partner composites as photocatalyst.

This section assesses how MMOF integrates with multimetal materials (like CdIn_*x*_S_*y*_, ZnIn_*x*_S_*y*_, bimetallic oxides, and other photocatalysis candidates) to form composites (Figure 15). The main purpose of constructing these heterojunctions is to improve charge separation, while the presence of multiple metal ions can also provide additional active sites and tune the electronic structure of the composites. This material is far more widely studied for H_2_ production than for other uses like degradation or CO_2_ reduction. MMOF/multimetal partner composites, with their sophisticated and engineered active sites, are applicable to H_2_ production and hold substantial potential within other photocatalytic processes. In many reported systems, the enhanced photocatalytic performance has been interpreted using proposed charge–transfer mechanisms such as Z‐scheme or S‐scheme heterojunctions. However, these mechanistic assignments are often based on indirect evidence and should therefore be interpreted with appropriate caution.

As observed in Table 4, MMOF/multimetallic partner composites have potential in diverse photocatalysis procedures. MMOFs can induce active sites, high specific surface area, and enhanced charge separation by producing mix‐valency, while the multimetal partner, besides providing more active sites, can reduce the band gap. For example, in multimetal partners containing Bi, the 6s orbital of Bi can reduce the band gap, or Ce doping into MOFs can produce an oxygen vacancy and regulate electron density around the second metal ion. Also, doping heteroatoms into the structure of MOFs can increase the attainable active sites and enhance the photocatalytic activity. The created heterojunctions of MMOF/multimetallic partner composites can buttress light absorption features, transformation of electron–hole pairs, reduce photo corrosion effect, and decrease Gibbs energy, which can be seen in diverse applications such as oxidations, degradations, CO_2_ reduction, and H_2_ production [278, 279, 281, 284, 285, 288, 289, 291].

**TABLE 4:** A summary of the photocatalytic performance for MMOF/multimetallic partner composites.

Name	Metals	Band gap (eV)	Application	Performance	References
ZnTi‐LDH/Cu‐FeTCPP	Fe/Cu/Zn/Ti	2.53	CO_2_ reduction	CO yield: 37.80 μmol g^−1^	[153]
Ce/Zr‐UiO‐66‐NH_2_/CdIn_2_S_4_	Zr/Ce/Cd/In	—	CO_2_ reduction	CO yield: 6.01 μmol g^−1^ h^−1^	[278]
Fe‐MOF(Ti)/BMO_0.4_	Fe/Mo/Ti/Bi	—	Removal	Cr(VI) removal efficiency: 96.5%	[279]
AGV/0.25HBF	Fe/Ag/V/Bi	2.62	Degradation	Degradation of RhB: reached 77.1% and 99.4%	[280]
Bi_2_MoO_6_/NH_2_‐UiO‐66(Zr/Ce)	Zr/Ce/Mo/Bi	—	Degradation	Photodegradation efficiency: 93.7% for oxytetracycline	[281]
ZnFe_2_O_4_@Co/Ni‐MOF	Fe/Ni/Co/Zn	2.34	Degradation	Removal efficiency of CR: 98%	[282]
Fe_3_O_4_@SiO_2_@PCN‐222(Fe)	Fe/Zr/Si	—	Degradation	Rate constant for RhB: 0.0202 min^−1^	[283]
BiVO_4_/NS‐FeCo‐MOFs	Fe/Co/V/Bi	2.30	Water splitting	P.d: 5.23 mA cm^−2^ at 1.23 V (RHE), IPCE: 74. 4% at 450 nm	[284]
Zn_0.5_Cd_0.5_S/NiCoZn‐LDH	Ni/Co/Zn/Cd	2.44	H_2_ production	1100 μmol g^−1^ h^−1^	[285]
ZCS@Cu_0.75_/Fe‐MOF	Fe/Cu/Zn/Cd	—	H_2_ production	1563.96 μmol g^−1^ h^−1^	[286]
Pd@UiO‐66‐NH_2_@ZnIn_2_S_4_	Zr/Zn/Pd/In	2.55	H_2_ production	5.26 mmol g^−1^ h^−1^	[287]
Zn_0.2_Cd_0.8_S/Zn_0.2_Cd_0.8_‐MOF	Zn/Cd	—	H_2_ production	13.3 mmol g^−1^ h^−1^	[288]
CuIn‐PMOFs@CdIn_2_S_4_	Cu/Cd/In	—	H_2_ production	7527.54 μmol g^−1^ h^−1^	[289]
NiCoP@ZnCoMOF	Ni/Co/Zn	—	H_2_ production	8583.4 μmol g^−1^ h^−1^	[290]

Abbreviations: BMO, Bi_2_MoO_6_; IPCE, incident photon‐to‐charge conversion efficiency; P.d., photocurrent density.

**FIGURE 16:**
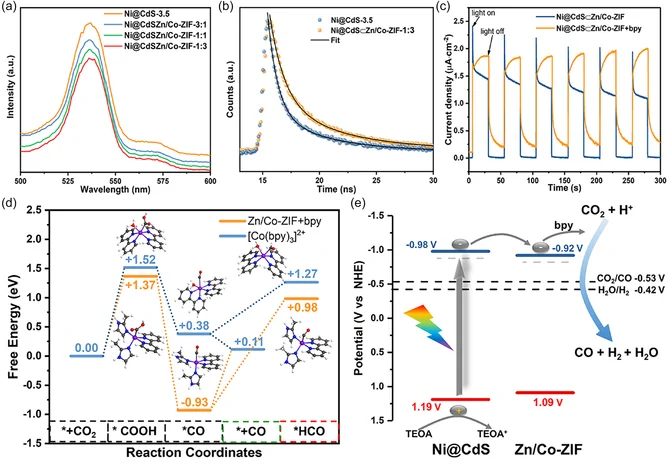
(a) Steady‐state PL spectra of Ni@CdS⊂Zn/Co‐ZIF. (b) Transient PL spectra of Ni@CdS‐3.5 and Ni@CdS⊂Zn/Co‐ZIF‐1:3. (c) Photocurrent responses of CO_2_ reduction system. (d) DFT calculations. (e) Schematic illustrations of the band‐level alignment in CO_2_RR and HER process. Reproduced with permission [293]. Copyright 2022, American Chemical Society.

For instance, a bimetallic NiFe‐MOFs cocatalyst was synthesized and constructed with BiVO_4_ as a novel composite photoanode. The photocurrent density is augmented by about 300%, and its stability is also remarkably boosted. The superior photocatalytic activity of PEC is related to the bimetallic MOFs that can facilitate the transfer/separation of carriers and have a higher specific surface area with providing rich active sites, thereby elevating the water oxidation procedure [292]. In another instance, the NiCoP@ZnCo‐MOF composite illustrates the premier activity toward H_2_ production application via data activity of H_2_ production rate of 8583.4 μmol g^−1^ h^−1^, and the apparent quantum yield (AQY) of 20.1% at 590 nm, which is higher compared to the ZnCo‐MOF. This high photocatalytic activity can be attributed to the operative n−n heterojunction charge separation and transport and swift H_2_ production reaction dynamics by the NiCoP cocatalyst [290]. In another instance, Ni@CdS⊂Zn/Co‐ZIF exhibits high activity toward CO_2_ reduction, as their performance and mechanism are displayed in Figure 16. The high photocatalytic activity can be related to the “2 + 2” synergic interactions, which regulate Ni doping into CdS NPs, buttressing the light‐harvesting ability and the solar energy utilization, while coating a bimetallic MOF shell on the hybrid Ni@CdS NPs not only optimized the CO_2_ adsorption‐activation procedure but also improved photogenerated charge/carrier separation and transport [293].

## 6 MMOFs/Carbon‐Based Partner Composites

**FIGURE 17:**
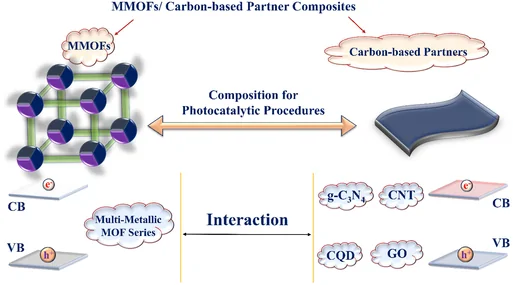
Schematic representation of MMOFs/carbon‐based partner composites as photocatalysts.

Multimetallic‐organic framework/carbon‐based partner composites have a dual architecture: an MMOF component and a carbon‐based component (Figure 17). Carbon nanotubes (CNTs), graphene materials, graphdiyne, etc., are all examples of carbon‐based partners. However, because of their relatively low photocatalytic activity compared to other MMOF composites, these materials haven’t been studied extensively. However, modifying their structures and synthesis methods may allow them to be used in photocatalysis in the future. The most commonly used applications for these materials are pertinent to degradation applications. Primarily, the MMOF offers active sites, whereas carbon materials impart conductivity and stability to the resulting composites. Besides degradation, H_2_ production and CO_2_ reduction, while reported, are under‐researched and offer avenues for future study, leveraging the same properties as degradation. In many reported MMOF/carbon‐based composites, the enhanced photocatalytic performance has been interpreted using proposed Z‐scheme or S‐scheme charge–transfer mechanisms. However, these mechanistic assignments are often based on indirect evidence and should therefore be interpreted with appropriate caution.

As can be observed in Table 5, MMOFs/carbon‐based partner composites have potential in diverse photocatalysis procedures, such as degradation [294], CO_2_ reduction [303], H_2_ production [301], cycloaddition [304], and antibiotic applications [298]. MMOFs and their interaction with carbon‐based materials can be beneficial for photocatalysts. When the carbon materials form heterogeneous bonds with the MMOF composite, electron flow occurs from the carbon material to the MMOF structure until an energy balance is established in the system. As a result, an internal electric field is generated at the interface and drives the charge transfer from the carbon material to the MMOF. The electric field facilitates the transfer of charges, which leads to improved photocatalytic performance [303]. For instance, in the H_2_ production applications, NaBH_4_ can be used as an H_2_ source, but its limitation in ambient conditions leads us to produce MMOF/carbon‐based partner composites. MOFs, due to their porous architecture, can augment activity, while materials such as CNT can induce stability to the structure which increase durability of the heterojunction [295]. Furthermore, in MMOF/GO‐based partner composites, the presence of MMOF can produce radicals that can affect catalysis efficiency by altering the interconvertible oxidation states, while GO induces enhancement to the band gap and visible light assimilation. MMOF/GO‐based partner composites can buttress charge transfer and water dispersibility, which can be beneficial for photocatalysis procedures [296, 297]. Also, in MMOF/graphitic carbon nitride composites, the Z‐scheme mechanism can be triggered. in some cases, photogenerated electron and hole recombination can be optimized properly, resulting in better photocatalytic activity compared to pristine MMOF without a partner [299, 305]. Eventually, MMOF/g‐C_*n*_H_2n−2_ composites can also trigger the S‐scheme mechanism and boost electron–hole features and efficient transportation charge. g‐C_*n*_H_2n−2_ can induce light absorption features in the MMOF that can buttress photocatalytic activity [306].

**TABLE 5:** Summary of the photocatalytic performance for MMOF/carbon‐based partner composites.

Name	Metals	Band gap (eV)	Application	Performance	References
GCN/Mn‐FeBTC	Mn/Fe	2.65	Degradation	Elimination of RR‐195 dye: 100%	[294]
Pd@Ce‐MOF@CNT	Pd/Ce	—	Degradation	98% RhB	[295]
CoZn@MOF/GO	Co/Zn	2.66	Degradation	TCn removal: 100%	[296]
NiCu@INA/rGO‐20	Ni/Cu	2.89	Degradation	TCn removal: 89%	[297]
NiFe‐MOF/CQD	Ni/Fe	—	Degradation	Rifampicin in a photo‐Fenton system: 84.9%	[298]
ZnCMOF/g‐C_3_N_4_	Zn/Co	—	H_2_ production	1040.1 μmol g^−1^ h^−1^	[299]
g‐C_3_N_4_/UiO‐66(Zr/Ce)	Zr/Ce	2.48	CO_2_ reduction	CH_3_OH: 54.71 μmol g^−1^ h^−1^ and C_2_H_5_OH: 38.10 μmol g^−1^ h^−1^	[300]
PVDF/Cu–NH_2_‐MIL‐125(Ti)	Cu/Ti	—	H_2_ production	389.1 μmol h^−1^ m^−2^	[301]
Graphdiyne/CoMo‐MOF	Co/Mo	—	H_2_ production	300 μmol	[302]

Abbreviations: GCN, graphitic carbon nitrides; RR‐195, reactive red 195; TCn, un‐metabolized tetracycline.

**FIGURE 18:**
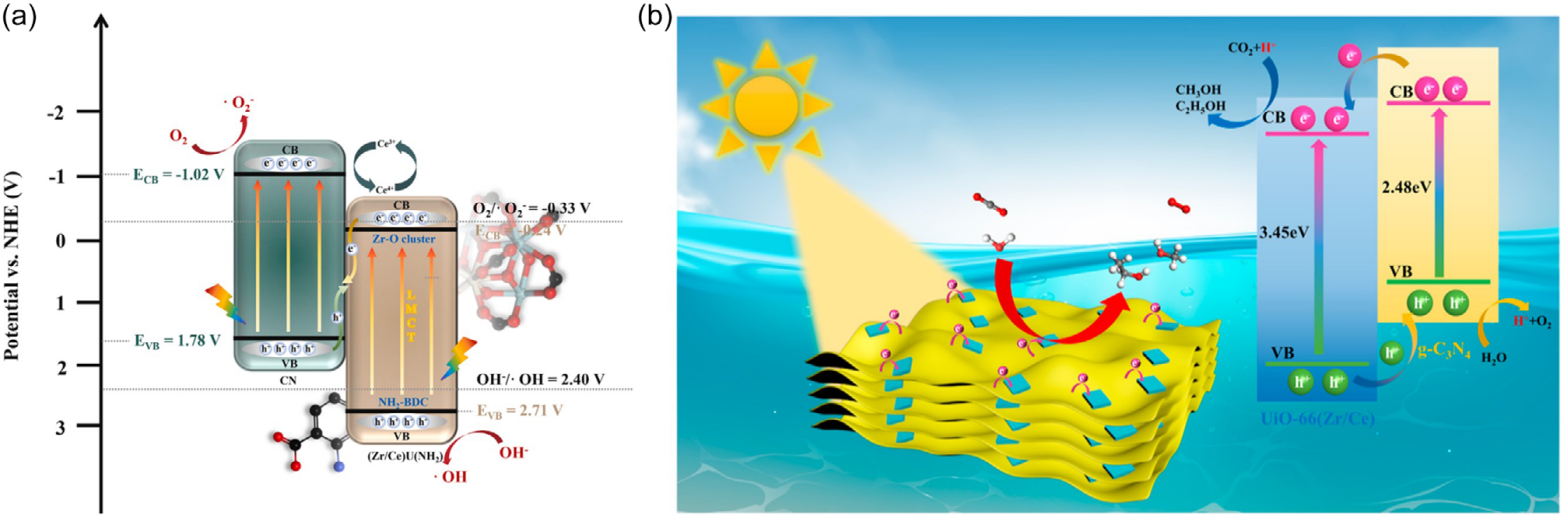
(a) Proposed mechanism of (Zr/Ce)U(NH_2_)@CN in TC removal, reproduced with permission [307]. Copyright 2023, Elsevier. (b) Proposed mechanism of g‐C_3_N_4_/UiO‐66(Zr/Ce) in the CO_2_ reduction. Reproduced with permission [300]. Copyright 2023, American Chemical Society.

For instance, (Zr/Ce)U(NH_2_)@CN was used for TC removal via triggering the Z‐scheme heterojunction due to the incorporation of g‐C_3_N_4_. g‐C_3_N_4_ could enhance the precursor dispersion, while (Zr/Ce)U(NH_2_) with decreased particle size grew densely on it. Data activity demonstrates that the kinetic constant was 23.73 and 5.89 times higher for UiO‐66 and CN, whose mechanism of TC degradation is exhibited in Figure 18a. Zr/Ce buttress LMCT due to replacement of Ce with Zr in the architecture, and the 4f orbital of Ce^4+^ can augment the light absorption and boost the LMCT. The (Zr/Ce)U(NH_2_)@CN Z‐scheme heterojunction improves electron transfer. Eventually, the electrons on the CB (MOF) recombined with the VB (g‐C_3_N_4_) holes, releasing g‐C_3_N_4_ electrons with potent reduction capability and VB holes with potent oxidation ability, which can interact with the generated radicals and efficiently remove TC [307]. In another instance, g‐C_3_N_4_/UiO‐66(Zr/Ce) is used for the CO_2_ reduction and exhibits the conversion of CO_2_ to CH_3_OH and C_2_H_5_OH in order 54.71, and 38.10 μmol g^−1^ h^−1^, respectively. The mechanism (Figure 18b) illustrated that g‐C_3_N_4_ with a narrow band gap generates electrons under visible light irradiation, and photoelectrons are transported to bimetallic MOF through the N−Zr/Ce−O chemical bond via the interaction of g‐C_3_N_4_ and UiO‐66(Zr/Ce), thereby reducing CO_2_ adsorbed on bimetallic MOF to CH_3_OH and C_2_H_5_OH [300].

## 7 Conclusion

MMOFs‐based composites show great potential in photocatalytic reactions. Introducing additional metal ions into MOFs has emerged as an effective strategy for improving the photocatalytic efficiency. MMCT between different metal ions facilitates charge transfer and charge carrier separation. In addition, the presence of metals with different natures leads to an increase in active sites and improved light‐absorption properties. Common oxide‐based multimetal composites MTi_2_O_6_, Bi_2_MO_6,_ and MVO_4_ have limitations such as low solar energy conversion efficiency, rapid recombination of the generated electron–hole pairs, wide band gaps, and absorption of energies higher than visible radiation for desirable photocatalytic performance. Due to the unique structural features of MOFs, such as high surface area, regular porosity, and various structural design capabilities, such as the use of proper functional groups and organic ligands, these materials are ideal options to overcome the limitations of conventional semiconductors. The high surface area of MOFs not only increases the adsorption capacity but also facilitates the interaction with reactants, prevents the aggregation of NPs in the composite, and therefore increases the catalytic efficiency. On the other hand, the limitations of MOFs in the photocatalytic process, such as weak conductivity and low stability, can be largely overcome by coupling them with other materials. Many studies have attributed the enhanced photocatalytic performance of MMOF composites to the formation of proposed Z‐scheme or S‐scheme heterojunctions, which are thought to promote efficient charge separation while preserving strong redox capability. In recent years, S‐scheme heterojunctions have attracted increasing attention because they provide a favorable framework for maintaining highly reducing electrons and strongly oxidizing holes while facilitating charge separation. Consequently, the S‐scheme design has emerged as an important strategy for developing high‐performance MMOF‐based photocatalysts. These mechanistic models are commonly used to explain the enhanced photocatalytic performance reported for MMOF‐based composites in applications such as pollutant degradation, CO_2_ reduction, and H_2_ production. However, in many reported systems, the assignment of Z‐scheme or S‐scheme charge–transfer pathways is primarily based on indirect experimental evidence rather than direct mechanistic verification. Therefore, these mechanistic interpretations should be considered with appropriate caution and, where possible, supported by complementary characterization techniques and theoretical calculations to achieve a more reliable understanding of charge–transfer processes. The meaningful relationships between the structural features of MOFs and rational coupling with other materials can aid to enhance the resulting photocatalytic efficiency. Understanding the mechanism of action of the components of multimetallic MOF composites is crucial for developing new methods to produce advanced structures with optimal catalytic performance. Currently, the solvothermal method is commonly used to prepare MMOF composites, which is not cost‐effective and is not easy to perform on a large scale. Therefore, finding an easy and rapid method could lead to further development of these materials. In addition, future research could focus on methods to prepare highly stable MOFs containing photoactive ligands or introducing semiconducting groups into the MOF structure through post‐synthetic methods. In addition, the relatively low photocatalytic activity of some MMOFs and partner materials, including certain carbon‐based materials and metal oxides/sulfides, remains a challenge. Developing new synthetic strategies to optimize their electronic structure and interfacial interactions will be important for further improving the photocatalytic performance. In addition, the use of more standardized photocatalytic testing and reporting protocols would facilitate more meaningful comparisons among MMOF‐based photocatalysts and help identify the most effective material design strategies. Future research should place greater emphasis on the rational design and comprehensive mechanistic characterization of MMOF composites, particularly those based on S‐scheme heterojunctions. Although techniques such as ESR spectroscopy, Mott‐Schottky analysis, XPS, and photoelectrochemical measurements are widely used to investigate charge–transfer processes, each provides only partial information and cannot independently establish the exact charge–transfer pathway. Combining complementary characterization techniques, particularly in situ/operando methods, with theoretical calculations will provide a more reliable understanding of charge–transfer dynamics and heterojunction mechanisms at the atomic level. Such advances in mechanistic understanding, together with the rational design of stable and efficient MMOF composites, are expected to accelerate the development of next‐generation photocatalysts for sustainable energy conversion and environmental remediation.

## Funding

This study was supported by the Tarbiat Modares University.

## Conflicts of Interest

The authors declare no conflicts of interest.

## Data Availability

No new data were created or analysed in this study. Data sharing is not applicable to this article.

## References

[1] D. Li , S. H. Yu , and H. L. Jiang , “From UV to Near‐Infrared Light‐Responsive Metal–organic Framework Composites: Plasmon and Upconversion Enhanced Photocatalysis,” Advanced Materials 30 (2018): 1707377. 10.1002/adma.201707377 29766571

[2] S.‐J. Wang , P. S. Abhari , B. Habibi , S. Z. Hashemi , X.‐W. Yan , and A. Morsali , “Trimetallic MOF‐Derived Ultrathin Nanosheet Photocatalyst for Efficient Visible‐light Antibiotic Degradation,” Inorganic Chemistry Communications 190 (2026): 116844.

[3] X. Yang and D. Wang , “Photocatalysis: From Fundamental Principles to Materials and Applications,” ACS Applied Energy Materials 1 (2018): 6657–6693.

[4] J.‐D. Xiao and H.‐L. Jiang , “Metal–organic Frameworks for Photocatalysis and Photothermal Catalysis,” Accounts of Chemical Research 52 (2018): 356–366. 10.1021/acs.accounts.8b00521 30571078

[5] R. Heu , M. Ateia , D. Awfa , P. Punyapalakul , and C. Yoshimura , “Photocatalytic Degradation of Organic Micropollutants in Water by Zr‐MOF/GO Composites,” Journal of Composites Science 4 (2020): 54.

[6] S. Abedi and A. Morsali , “Ordered Mesoporous Metal–organic Frameworks Incorporated With Amorphous TiO_2_ as Photocatalyst for Selective Aerobic Oxidation in Sunlight Irradiation,” ACS Catalysis 4 (2014): 1398–1403.

[7] F. Afshariazar and A. Morsali , “Mixed‐Valence Metal–organic Frameworks: Concepts, Opportunities, and Prospects,” Chemical Society Reviews 54 (2025): 1318–1383. 10.1039/d4cs01061b 39704326

[8] Y. R. Wang , H. M. Ding , X. Y. Ma , et al., “Imparting CO_2_ Electroreduction Auxiliary for Integrated Morphology Tuning and Performance Boosting in a Porphyrin‐Based Covalent Organic Framework,” Angewandte Chemie International Edition 61 (2022): e202114648. 10.1002/anie.202114648 34806265

[9] Z. Davoudi , Z. Sharifzadeh , S. A. A. Razavi , and A. Morsali , “Probing the Adsorption Behavior of the Amine Environment in as(III) Direct Removal via Postsynthetic Functionalization of MOF‐808 With Amino Acids,” Inorganic Chemistry 64 (2025): 24662–24673, 10.1021/acs.inorgchem.5c04322. 41345055

[10] Y. Peng , S. Sanati , A. Morsali , and H. García , “Metal–Organic Frameworks as Electrocatalysts,” Angewandte Chemie International Edition 62 (2023): e202214707, 10.1002/anie.202214707. 36468543

[11] M. Nazari and A. Morsali , “Trimetallic‐Organic Framework/MXene Composite as an Oxygen Evolution Reaction Electrocatalyst With Elevated Intrinsic Activity,” Journal of Materials Chemistry A 12 (2024): 4826–4834, 10.1039/D4TA00131A.

[12] S. Sanati , A. Morsali , and H. García , “First‐Row Transition Metal‐Based Materials Derived From Bimetallic Metal–organic Frameworks as Highly Efficient Electrocatalysts for Electrochemical Water Splitting,” Energy & Environmental Science 15 (2022): 3119–3151, 10.1039/D1EE03614A.

[13] S. Sharifinia , M. Mazraeh , and A. Morsali , “The Impact of Mixed‐Linker Engineering on MOF's Electrocatalytic Activity,” Coordination Chemistry Reviews 560 (2026): 217782, 10.1016/j.ccr.2026.217782.

[14] K.‐G. Liu , F. Bigdeli , A. Panjehpour , S. Hwa Jhung , H. A. J. Al Lawati , and A. Morsali , “Potential Applications of MOF Composites as Selective Membranes for Separation of Gases,” Coordination Chemistry Reviews 496 (2023): 215413.

[15] T. Yang , F. Bigdeli , S. Z. Hashemi , S. Karimi , K.‐G. Liu , and A. Morsali , “Recent Advances in Selective Separation of H_2_/CO_2_ Gases by Composites of MOFs,” Renewable and Sustainable Energy Reviews 229 (2026): 116630, 10.1016/j.rser.2025.116630.

[16] R. R. Moslehabadi , Z. Hedayati , M. Mazraeh , S. Asghar , F. Rouhani , and A. Morsali , “Sacrificial MOFs on usage: Multifunctional Compounds,” Journal of Materials Chemistry B 13 (2025): 7554–7608, 10.1039/D5TB00764J. 40468787

[17] L. E. Kreno , K. Leong , O. K. Farha , M. Allendorf , R. P. Van Duyne , and J. T. Hupp , “Metal–Organic Framework Materials as Chemical Sensors,” Chemical Reviews 112 (2012): 1105–1125, 10.1021/cr200324t. 22070233

[18] L. Wu , L. Zhang , R. Liu , et al., “2D/2D g‐C_3_N_4_/1T‐MoS_2_ Nanohybrids as Schottky Heterojunction Photocatalysts for Nuclear Wastewater Pretreatment,” ACS ES&T Water 1 (2021): 2197–2205.

[19] Q. Wang , Q. Gao , A. M. Al‐Enizi , A. Nafady , and S. Ma , “Recent Advances in MOF‐Based Photocatalysis: Environmental Remediation Under Visible Light,” Inorganic Chemistry Frontiers 7 (2020): 300–339.

[20] H.‐Q. Xu , S. Yang , X. Ma , J. Huang , and H.‐L. Jiang , “Unveiling Charge‐Separation Dynamics in CdS/Metal–organic Framework Composites for Enhanced Photocatalysis,” ACS Catalysis 8 (2018): 11615–11621.

[21] M. Mazraeh , Z. Hedayati , A. Primo , A. Dhakshinamoorthy , A. Morsali , and H. Garcia , “Recent Advances in Multi‐Metallic Organic Frameworks and Their Derivatives for Catalysis, Electro‐/Photocatalysis and Sensing Applications,” Journal of Materials Chemistry A 14 (2026): 13238–13285, 10.1039/d5ta06669g.

[22] K.‐G. Liu , F. Bigdeli , A. Panjehpour , et al., “Metal Organic Framework Composites for Reduction of CO_2_ ,” Coordination Chemistry Reviews 493 (2023): 215257.

[23] F. L. Li , P. Wang , X. Huang , et al., “Large‐Scale, Bottom‐up Synthesis of Binary Metal–organic Framework Nanosheets for Efficient Water Oxidation,” Angewandte Chemie 131 (2019): 7125–7130. 10.1002/anie.201902588 30913361

[24] R. Abazari , S. Sanati , and A. Morsali , “Mixed Metal Fe_2_Ni MIL‐88B Metal–organic Frameworks Decorated on Reduced Graphene Oxide as a Robust and Highly Efficient Electrocatalyst for Alkaline Water Oxidation,” Inorganic Chemistry 61 (2022): 3396–3405. 10.1021/acs.inorgchem.1c03216 35157424

[25] M. Mazraeh , A. Dhakshinamoorthy , A. Morsali , and H. Garcia , “Multi‐Metallic Organic Framework‐Based Composites as Electrocatalysts,” Small 22 (2026): e11734, 10.1002/smll.202511734. PMC13040138 41718691

[26] D. Yang , and B. C. Gates , “Catalysis by Metal Organic Frameworks: Perspective and Suggestions for Future Research,” ACS Catalysis 9 (2019): 1779–1798.

[27] C. Li , L. Zhang , J. Chen , et al., “Recent Development and Applications of Electrical Conductive MOFs,” Nanoscale 13 (2021): 485–509. 10.1039/d0nr06396g 33404574

[28] J. Lee and J. Kim , “Cooperative Design of the Ag_3_PO_4_/NH_2_‐MIL‐88B (Fe/Co) Heterojunction Integrated With Conductive Polypyrrole for Advanced Photocatalytic Water Purification,” ACS Omega 9 (2024): 5942–5953. 10.1021/acsomega.3c09625 PMC10851240 38343939

[29] M. E. Farshchi , K. Asgharizadeh , H. Jalili , et al., “Bimetallic MOF@CdS Nanorod Composite for Highly Efficient Piezo‐Photocatalytic CO_2_ Methanation Under Visible Light,” Journal of Environmental Chemical Engineering 12 (2024): 113909.

[30] M. Alvaro , E. Carbonell , B. Ferrer , F. X. Llabrés i Xamena , and H. Garcia , “Semiconductor Behavior of a Metal‐Organic Framework (MOF),” Chemistry – A European Journal 13 (2007): 5106–5112. 10.1002/chem.200601003 17385196

[31] X. Li , Y. Pi , Q. Xia , Z. Li , and J. Xiao , “TiO_2_ Encapsulated in Salicylaldehyde‐NH_2_‐MIL‐101 (Cr) for Enhanced Visible Light‐Driven Photodegradation of MB,” Applied Catalysis B: Environmental 191 (2016): 192–201.

[32] Y. Zhao , Y. Dong , F. Lu , et al., “Coordinative Integration of a Metal‐Porphyrinic Framework and TiO_2_ Nanoparticles for the Formation of Composite Photocatalysts With Enhanced Visible‐Light‐Driven Photocatalytic Activities,” Journal of Materials Chemistry A 5 (2017): 15380–15389.

[33] L.‐M. Yang , G.‐Y. Fang , J. Ma , E. Ganz , and S. S. Han , “Band Gap Engineering of Paradigm MOF‐5,” Crystal Growth & Design 14 (2014): 2532–2541.

[34] J. Nicks , K. Sasitharan , R. R. Prasad , D. J. Ashworth , and J. A. Foster , “Metal–organic Framework Nanosheets: Programmable 2D Materials for Catalysis, Sensing, Electronics, and Separation Applications,” Advanced Functional Materials 31 (2021): 2103723.

[35] A. Melillo , M. Cabrero‐Antonino , S. Navalón , M. Álvaro , B. Ferrer , and H. García , “Enhancing Visible‐Light Photocatalytic Activity for Overall Water Splitting in UiO‐66 by Controlling Metal Node Composition,” Applied Catalysis B: Environmental 278 (2020): 119345.

[36] C. L. Warkentin , Z. Yu , A. Sarkar , and R. R. Frontiera , “Decoding Chemical and Physical Processes Driving Plasmonic Photocatalysis Using Surface‐Enhanced Raman Spectroscopies,” Accounts of Chemical Research 54 (2021): 2457–2466. 10.1021/acs.accounts.1c00088 33957039

[37] F. Liu , J. Cao , Z. Yang , et al., “Heterogeneous Activation of Peroxymonosulfate by Cobalt‐Doped MIL‐53 (Al) for Efficient Tetracycline Degradation in Water: Coexistence of Radical and Non‐Radical Reactions,” Journal of Colloid and Interface Science 581 (2021): 195–204. 10.1016/j.jcis.2020.07.100 32771731

[38] L. Chen , H.‐F. Wang , C. Li , and Q. Xu , “Bimetallic Metal–organic Frameworks and Their Derivatives,” Chemical Science 11 (2020): 5369–5403. 10.1039/d0sc01432j PMC8159423 34094065

[39] J. Li , M. Zhang , Q. Li , and J. Yang , “Enhanced Visible Light Activity on Direct Contact Z‐Scheme g‐C_3_N_4_‐TiO_2_ Photocatalyst,” Applied Surface Science 391 (2017): 184–193.

[40] D. Roy , S. Neogi , and S. De , “Visible Light Assisted Activation of Peroxymonosulfate by Bimetallic MOF Based Heterojunction MIL‐53 (Fe/Co)/CeO_2_ for Atrazine Degradation: Pivotal Roles of Dual Redox Cycle for Reactive Species Generation,” Chemical Engineering Journal 430 (2022): 133069.

[41] R. Medhi , M. D. Marquez , and T. R. Lee , “Visible‐Light‐Active Doped Metal Oxide Nanoparticles: Review of Their Synthesis, Properties, and Applications,” ACS Applied Nano Materials 3 (2020): 6156–6185.

[42] L.‐B. Hou , H. N. Catherine , K. Harada , M. Yoshida , Y.‐L. Chen , and C. Hu , “Reactive Seeding Growth of Cobalt‐Doped MIL‐88B (Fe) on Al_2_O_3_ Membrane for Phenol Removal in a Photocatalytic Membrane Reactor,” Journal of Membrane Science 680 (2023): 121730.

[43] S. Abednatanzi , P. G. Derakhshandeh , H. Depauw , et al., “Mixed‐Metal Metal–organic Frameworks,” Chemical Society Reviews 48 (2019): 2535–2565. 10.1039/c8cs00337h 30989162

[44] K. Tian , Y. Ma , Y. Liu , et al., “Hierarchically Structured Hollow Bimetallic ZnNi MOF Microspheres as a Sensing Platform for Adenosine Detection,” Sensors and Actuators B: Chemical 303 (2020): 127199.

[45] M. Zhang , W. Xu , T. Li , H. Zhu , and Y. Zheng , “In Situ Growth of Tetrametallic FeCoMnNi‐MOF‐74 on Nickel Foam as Efficient Bifunctional Electrocatalysts for the Evolution Reaction of Oxygen and Hydrogen,” Inorganic Chemistry 59 (2020): 15467–15477. 10.1021/acs.inorgchem.0c02504 32991151

[46] S. Wang , Z. Li , F. Duan , et al., “Bimetallic Cerium/Copper Organic Framework‐Derived Cerium and Copper Oxides Embedded by Mesoporous Carbon: Label‐Free Aptasensor for Ultrasensitive Tobramycin Detection,” Analytica Chimica Acta 1047 (2019): 150–162. 10.1016/j.aca.2018.09.064 30567645

[47] S. Kim and A. Muthurasu , “Metal‐Organic Framework–assisted Bimetallic Ni@Cu Microsphere for Enzyme‐Free Electrochemical Sensing of Glucose,” Journal of Electroanalytical Chemistry 873 (2020): 114356.

[48] D. S. Raja , C.‐L. Huang , Y.‐A. Chen , Y. Choi , and S.‐Y. Lu , “Composition‐Balanced Trimetallic MOFs as Ultra‐Efficient Electrocatalysts for Oxygen Evolution Reaction at High Current Densities,” Applied Catalysis B: Environmental 279 (2020): 119375.

[49] S. Liang , Y. Chen , W. Han , Y. Jiao , W. Li , and G. Tian , “Hierarchical S‐Scheme Titanium Dioxide@Cobalt‐Nickel Based Metal–organic Framework Nanotube Photocatalyst for Selective Carbon Dioxide Photoreduction to Methane,” Journal of Colloid and Interface Science 630 (2023): 11–22. 10.1016/j.jcis.2022.09.115 36215820

[50] J. Chen , S. Zhang , Y. Xu , et al., “Multiple Strategies Enhance Visible‐Light Photocatalytic Degradation Levofloxacin of Fe‐Doped MOF/AgCl Z‐Scheme Heterojunction Composites Based on Pyrazole Carboxylic Ligand,” Applied Surface Science 677 (2024): 160994.

[51] N. Q. Tran , T. T. Truong , T. T. N. Tran , et al., “Multi‐Metallic Metal–Organic Framework Nanosheets With 3D Flower‐Like Nanostructure‐Based Natural Seawater Splitting Toward Stable Industrial‐Scale Current Density,” ACS Sustainable Chemistry & Engineering 12 (2024): 1038–1050.

[52] J. Liu , X. Sun , B. Jiang , et al., “UiO‐66‐NH_2_ Octahedral Nanocrystals Decorated With ZnFe_2_O_4_ Nanoparticles for Photocatalytic Alcohol Oxidation,” ACS Applied Nano Materials 5 (2022): 2231–2240.

[53] W. Shi , Y. Fu , H. Sun , et al., “Construction of 0D/3D CoFe_2_O_4_/MIL‐101 (Fe) Complement Each Other S‐Scheme Heterojunction for Effectively Boosted Photocatalytic Degradation of Tetracycline,” Inorganic Chemistry Communications 146 (2022): 110140.

[54] Y. Zhang , Y. Yang , S. Yang , E. Quispe‐Cardenas , and M. R. Hoffmann , “Application of Heterojunction Ni–Sb–SnO_2_ Anodes for Electrochemical Water Treatment,” ACS ES&T Engineering 1 (2021): 1236–1245.

[55] S. Mansingh , K. K. Das , N. Priyadarshini , et al., “Minireview Elaborating S‐Scheme Charge Dynamic Photocatalysts: Journey From Z to S, Mechanism of Charge Flow, Characterization Proof, and H_2_O_2_ Evolution,” Energy & Fuels 37 (2023): 9873–9894.

[56] K. S. Ganesh , L. Fan , B. Wang , P. Jeevan Kumar , and B. Zhu , “Built‐in Electric Field for Efficient Charge Separation and Ionic Transport in LiCoO_2_/SnO_2_ Semiconductor Junction Fuel Cells,” ACS Applied Energy Materials 5 (2022): 12513–12522.

[57] M. Sayed , L. Zhang , H. Garcia , H. Yu , and J. Yu , “S‐Scheme Shapes Heterojunction Photocatalysis,” Accounts of Chemical Research 59 (2026): 1138–1153. 10.1021/acs.accounts.5c00899 41838418

[58] X. Wu , M. Sayed , G. Wang , W. Yu , and B. Zhu , “COF‐Based S‐Scheme Heterojunction Photocatalyst,” Advanced Materials 38 (2026): e11322. 10.1002/adma.202511322 40970807

[59] L. Zhang , J. Zhang , J. Yu , and H. García , “Charge‐Transfer Dynamics in S‐Scheme Photocatalyst,” Nature Reviews Chemistry 9 (2025): 328–342. 10.1038/s41570-025-00698-3 40097789

[60] H. Hu , L. Han , M. Yu , Z. Wang , and X. W. D. Lou , “Metal–organic‐Framework‐Engaged Formation of Co Nanoparticle‐Embedded Carbon@Co_9_S_8_ Double‐Shelled Nanocages for Efficient Oxygen Reduction,” Energy & Environmental Science 9 (2016): 107–111.

[61] F. Guo , H. Yang , L. Liu , et al., “Hollow Capsules of Doped Carbon Incorporating Metal@Metal Sulfide and Metal@metal Oxide Core–shell Nanoparticles Derived From Metal–organic Framework Composites for Efficient Oxygen Electrocatalysis,” Journal of Materials Chemistry A 7 (2019): 3624–3631.

[62] D. Mukherjee , B. Van der Bruggen , and B. Mandal , “Advancements in Visible Light Responsive MOF Composites for Photocatalytic Decontamination of Textile Wastewater: A Review,” Chemosphere 295 (2022): 133835. 10.1016/j.chemosphere.2022.133835 35122821

[63] M. Xie , Y. Ma , D. Lin , C. Xu , F. Xie , and W. Zeng , “Bimetal–organic Framework MIL‐53 (Co–Fe): An Efficient and Robust Electrocatalyst for the Oxygen Evolution Reaction,” Nanoscale 12 (2020): 67–71. 10.1039/c9nr06883j 31807741

[64] F. Li , Y. Tian , S. Su , et al., “Theoretical and Experimental Exploration of Tri‐Metallic Organic Frameworks (t‐MOFs) for Efficient Electrocatalytic Oxygen Evolution Reaction,” Applied Catalysis B: Environmental 299 (2021): 120665.

[65] H.‐L. Jiang , B. Liu , T. Akita , M. Haruta , H. Sakurai , and Q. Xu , “Au@ZIF‐8: CO Oxidation Over Gold Nanoparticles Deposited to Metal−Organic Framework,” Journal of the American Chemical Society 131 (2009): 11302–11303. 10.1021/ja9047653 19637919

[66] H.‐L. Jiang , T. Akita , T. Ishida , M. Haruta , and Q. Xu , “Synergistic Catalysis of Au@Ag Core−shell Nanoparticles Stabilized on Metal− Organic Framework,” Journal of the American Chemical Society 133 (2011): 1304–1306. 10.1021/ja1099006 21214205

[67] M. Ciprian , P. Xu , S. Chaemchuen , et al., “MoO_3_ Nanoparticle Formation on Zeolitic Imidazolate Framework‐8 by Rotary Chemical Vapor Deposition,” Microporous and Mesoporous Materials 267 (2018): 185–191.

[68] W. Zhou , L. Gao , Y. Zhang , and T. Hu , “Composites of Ni‐MOF and Polyaniline Hydrogel for Carbon Monoxide Resistant Excellent Catalysts of Ethanol Oxidation Reaction,” International Journal of Hydrogen Energy 46 (2021): 27128–27137.

[69] M. Kang , D. W. Kang , and C. S. Hong , “Post‐Synthetic Diamine‐Functionalization of MOF‐74 Type Frameworks for Effective Carbon Dioxide Separation,” Dalton Transactions 48 (2019): 2263–2270. 10.1039/c8dt04339f 30693920

[70] B. Li , J. G. Ma , and P. Cheng , “Integration of Metal Nanoparticles Into Metal–organic Frameworks for Composite Catalysts: Design and Synthetic Strategy,” Small 15 (2019): 1804849. 10.1002/smll.201804849 30756464

[71] J. Guo , Y. Wan , Y. Zhu , M. Zhao , and Z. Tang , “Advanced Photocatalysts Based on Metal Nanoparticle/Metal‐Organic Framework Composites,” Nano Research 14 (2021): 2037–2052.

[72] Q. Han , Y. Dong , C. Xu , et al., “Immobilization of Metal–organic Framework MIL‐100 (Fe) on the Surface of BiVO_4_: A New Platform for Enhanced Visible‐Light‐Driven Water Oxidation,” ACS Applied Materials & Interfaces 12 (2020): 10410–10419. 10.1021/acsami.9b21507 32030977

[73] S. Yu , J. Li , Y. Du , Y. Wang , Y. Zhang , and Z. Wu , “Sulfur‐Modified MOFs as Efficient Electrocatalysts for Overall Water Splitting,” Coordination Chemistry Reviews 520 (2024): 216144.

[74] A. Akhoondi , U. Feleni , B. Bethi , A. O. Idris , and A. Hojjati‐Najafabadi , “Advances in Metal‐Based Vanadate Compound Photocatalysts: Synthesis, Properties and Applications,” Synthesis and Sintering 1 (2021): 151–168.

[75] T. Yao , Y. Tan , Y. Zhou , Y. Chen , and M. Xiang , “Preparation of Core‐Shell MOF‐5/Bi_2_WO_6_ Composite for the Enhanced Photocatalytic Degradation of Pollutants,” Journal of Solid State Chemistry 308 (2022): 122882.

[76] Q. Li , L. Li , X. Long , et al., “Rational Design of MIL‐88A(Fe)/Bi_2_WO_6_ Heterojunctions as an Efficient Photocatalyst for Organic Pollutant Degradation Under Visible Light Irradiation,” Optical Materials 118 (2021): 111260.

[77] H. Yi , L. Qin , D. Huang , et al., “Nano‐Structured Bismuth Tungstate With Controlled Morphology: Fabrication, Modification, Environmental Application and Mechanism Insight,” Chemical Engineering Journal 358 (2019): 480–496.

[78] S. Zhang , M. Du , J. Kuang , et al., “Surface‐Defect‐Rich Mesoporous NH_2_‐MIL‐125 (Ti)@Bi_2_MoO_6_ Core‐Shell Heterojunction With Improved Charge Separation and Enhanced Visible‐Light‐Driven Photocatalytic Performance,” Journal of Colloid and Interface Science 554 (2019): 324–334. 10.1016/j.jcis.2019.07.021 31306944

[79] S. Song , Z. Song , H. Han , et al., “Enhanced Photocatalytic CO_2_ Reduction Activity on the Novel Z‐Scheme Co‐MOF/Bi_2_MoO_6_ to Form CO and CH_4_ ,” Applied Catalysis A: General 683 (2024): 119834.

[80] H. Feng , Y. Sun , Q. Xu , and H. Liu , “Construction of NH_2_‐MIL‐101 (Fe)@Bi_2_MoO_6_ S‐Scheme Heterojunction for Efficient and Selective Photocatalytic CO_2_ Conversion to CO,” Applied Catalysis A: General 664 (2023): 119350.

[81] J. Ding , Z. Yang , C. He , et al., “UiO‐66 (Zr) Coupled With Bi_2_MoO_6_ as Photocatalyst for Visible‐Light Promoted Dye Degradation,” Journal of Colloid and Interface Science 497 (2017): 126–133. 10.1016/j.jcis.2017.02.060 28282564

[82] W. Gu , Q. Li , H. Zhu , and L. Zou , “Facile Interface Engineering of Hierarchical Flower Spherical‐Like Bi‐Metal–organic Framework Microsphere/Bi_2_MoO_6_ Heterostructure for High‐Performance Visible–light Photocatalytic Tetracycline Hydrochloride Degradation,” Journal of Colloid and Interface Science 606 (2022): 1998–2010. 10.1016/j.jcis.2021.10.004 34749447

[83] L. Cheng , M. Xie , Y. Sun , and H. Liu , “Bi_2_WO_6_‐Wrapped 2D Ni‐MOF Sheets With Significantly Improved Photocatalytic Activity by a Direct Z‐Scheme Electron Transfer,” Journal of Alloys and Compounds 896 (2022): 163055.

[84] G. Li , Y. Wang , R. Huang , et al., “In‐Situ Growth UiO‐66‐NH_2_ on the Bi_2_WO_6_ to Fabrication Z‐Scheme Heterojunction With Enhanced Visible‐Light Driven Photocatalytic Degradation Performance,” Colloids and Surfaces A: Physicochemical and Engineering Aspects 603 (2020): 125256.

[85] Q. Su , J. Li , B. Wang , Y. Li , and L. Hou , “Direct Z‐Scheme Bi_2_MoO_6_/UiO‐66‐NH_2_ Heterojunctions for Enhanced Photocatalytic Degradation of Ofloxacin and Ciprofloxacin Under Visible Light,” Applied Catalysis B: Environmental 318 (2022): 121820.

[86] P. Hu , C. Yao , L. Yang , Y. Xin , and Y. Miao , “Boosted Photodegradation of Tetracycline Hydrochloride Over Z‐Scheme MIL‐88B (Fe)/Bi_2_WO_6_ Composites Under Visible Light,” Colloids and Surfaces A: Physicochemical and Engineering Aspects 627 (2021): 127248.

[87] S. Li , C. Wang , Y. Liu , et al., “S‐Scheme MIL‐101 (Fe) Octahedrons Modified Bi_2_WO_6_ Microspheres for Photocatalytic Decontamination of Cr(VI) and Tetracycline Hydrochloride: Synergistic Insights, Reaction Pathways, and Toxicity Analysis,” Chemical Engineering Journal 455 (2023): 140943.

[88] Y. Xu , Y. Zhou , Y. Deng , et al., “Synthesis of Bi_2_WO_6_@NH_2_‐MIL‐125 (Ti): A S‐Scheme Photocatalyst With Enhanced Visible Light Catalytic Activity,” Catalysis Letters 150 (2020): 3470–3480.

[89] Y. Yang , M. Xu , L. Ai , et al., “In Situ Growth of Flower Sphere Bi_2_WO_6_/Bi‐MOF Heterojunction With Enhanced Photocatalytic Degradation of Pollutants: DFT Calculation and Mechanism,” Journal of Environmental Chemical Engineering 11 (2023): 109873.

[90] Z. Zhao , J. Bian , L. Zhao , et al., “Construction of 2D Zn‐MOF/BiVO_4_ S‐Scheme Heterojunction for Efficient Photocatalytic CO_2_ Conversion Under Visible Light Irradiation,” Chinese Journal of Catalysis 43 (2022): 1331–1340.

[91] X. Wang , J. You , Y. Xue , et al., “Structural Regulation of Three‐Dimensional Bismuth Vanadate Nanochannels for Excellent Visible Light Photocatalytic Nitrogen Fixation,” Applied Catalysis B: Environment and Energy 363 (2025): 124817.

[92] T. Zhou , J. Liu , H. Zhan , et al., “Facile Preparation of BiVO_4_/Bi‐MOF Composites for Photocatalytic Dye Removal,” Journal of Physics and Chemistry of Solids 188 (2024): 111917.

[93] H. Sun , Q. Dai , J. Liu , et al., “BiVO_4_–Deposited MIL–101–NH_2_ for Efficient Photocatalytic Elimination of Cr(VI),” Molecules 28 (2023): 1218. 10.3390/molecules28031218 PMC9921149 36770885

[94] A. Hekmat , S. Ghasemi , and M. Vossoughi , “Unveiling the Synergistic Effect of Ionic Liquid and Metal‐Organic Framework on the Efficiency of BiVO_4_: BiVO_4_‐MIL‐100(Fe) as a Visible Light‐Induced Photocatalyst for Basic Red 46 Degradation,” Journal of Environmental Chemical Engineering 11 (2023): 110658.

[95] X. Sun , J. Liu , H. Wang , et al., “In‐Situ Detection Technique for Charge Transfer Behavior of Direct Z‐Scheme BiVO_4_/UiO‐66‐NH_2_ Composites During Photocatalytic Thioanisole Conversion,” Chemical Engineering Journal 472 (2023): 144750.

[96] X. Li , C. Garlisi , Q. Guan , et al., “A Review of Material Aspects in Developing Direct Z‐Scheme Photocatalysts,” Materials Today 47 (2021): 75–107.

[97] H. Shi , C. Li , L. Wang , W. Wang , and X. Meng , “Selective Reduction of Nitrate Into N_2_ by Novel Z‐Scheme NH_2_‐MIL‐101 (Fe)/BiVO_4_ Heterojunction With Enhanced Photocatalytic Activity,” Journal of Hazardous Materials 424 (2022): 127711. 10.1016/j.jhazmat.2021.127711 34799158

[98] H. Li , H. Gong , and Z. Jin , “Phosphorus Modified Ni‐MOF–74/BiVO_4_ S‐Scheme Heterojunction for Enhanced Photocatalytic Hydrogen Evolution,” Applied Catalysis B: Environment and Energy 307 (2022): 121166.

[99] B. Zhai , Y. Chen , Y. Liang , et al., “Modifying Ag_3_VO_4_ With Metal‐Organic Frameworks for Enhanced Photocatalytic Activity Under Visible Light,” Materials Chemistry and Physics 239 (2020): 122078.

[100] E. Akbarzadeh , H. Z. Soheili , M. Hosseinifard , and M. R. Gholami , “Preparation and Characterization of Novel Ag_3_VO_4_/Cu‐MOF/rGO Heterojunction for Photocatalytic Degradation of Organic Pollutants,” Materials Research Bulletin 121 (2020): 110621.

[101] B. Zhai , Y. Chen , and Y. Liang , “In Situ Preparation of Ag_3_VO_4_/MOFs Composites With Enhanced Visible‐Light‐Driven Catalytic Activity,” Journal of Nanoparticle Research 21 (2019): 1–15.

[102] S. K. Sharma , A. Kumar , P. Dhiman , G. Sharma , and F. J. Stadler , “ZIF‐67/Ag_3_VO_4_ Based S‐Scheme Heterojunction for Visible Light Driven Rapid Photocatalytic Removal of Venlafaxine,” Optical Materials 146 (2023): 114541.

[103] A. Kuila , P. Saravanan , S. Routu , P. Gopinath , M. Jang , and C. Wang , “Improved Charge Carrier Dynamics Through a Type II Staggered Ce MOF/Mc BiVO_4_ Nn Heterojunction for Enhanced Visible Light Utilisation,” Applied Surface Science 553 (2021): 149556.

[104] Y. Sun , Y. Li , J. He , et al., “Controlled Synthesis of Mn_x_Cd_1–x_S for Enhanced Visible‐Light Driven Photocatalytic Hydrogen Evolution,” Chinese Journal of Structural Chemistry 42 (2023): 100145.

[105] H. Gong , X. Zhang , G. Wang , Y. Liu , Y. Li , and Z. Jin , “Dodecahedron ZIF‐67 Anchoring ZnCdS Particles for Photocatalytic Hydrogen Evolution,” Molecular Catalysis 485 (2020): 110832.

[106] J. Qiu , X.‐F. Zhang , X. Zhang , et al., “Constructing Cd_0.5_Zn_0.5_S@ZIF‐8 Nanocomposites Through Self‐Assembly Strategy to Enhance Cr(VI) Photocatalytic Reduction,” Journal of Hazardous Materials 349 (2018): 234–241. 10.1016/j.jhazmat.2018.02.009 29428684

[107] Y. Su , Z. Zhang , H. Liu , and Y. Wang , “Cd_0.2_Zn_0.8_S@UiO‐66‐NH_2_ Nanocomposites as Efficient and Stable Visible‐Light‐Driven Photocatalyst for H_2_ Evolution and CO_2_ Reduction,” Applied Catalysis B: Environmental 200 (2017): 448–457.

[108] S. P. Tripathy , S. Subudhi , A. Ray , et al., “MgIn_2_S_4_/UiO‐66‐NH_2_ MOF‐Based Heterostructure: Visible‐Light‐Responsive Z‐Scheme‐Mediated Synergistically Enhanced Photocatalytic Performance Toward Hydrogen and Oxygen Evolution,” Langmuir 39 (2023): 7294–7306. 10.1021/acs.langmuir.3c00151 37184616

[109] M. Li , J. Li , and Z. Jin , “0D/2D Spatial Structure of Cd_x_Zn_1−x_S/Ni‐MOF‐74 for Efficient Photocatalytic Hydrogen Evolution,” Dalton Transactions 49 (2020): 5143–5156. 10.1039/d0dt00271b 32236209

[110] E. M. Khudhair , W. N. Khudhair , S. H. Ammar , and A. S. Mahdi , “Assembling ZIF‐67@Cd_0.5_Zn_0.5_S Nanocomposites With an Enhanced Photocatalytic Activity,” Inorganic Chemistry Communications 142 (2022): 109639.

[111] S. Yang , S. Peng , C. Zhang , X. He , and Y. Cai , “Synthesis of Cd* _x_ * Zn_1− *x* _S@MIL‐101(Cr) Composite Catalysts for the Photodegradation of Methylene Blue,” Nano 13 (2018): 1850118.

[112] M. Ayyob , A. Daud , Z. Sun , et al., “Metal‐Organic Framework‐Templated ZnIn_2_S_4_ Nanomaterials for High‐Performance Photocatalytic H_2_‐Evolution,” International Journal of Hydrogen Energy 64 (2024): 914–925.

[113] S. Zhang , M. Du , Z. Xing , Z. Li , K. Pan , and W. Zhou , “Defect‐Rich and Electron‐Rich Mesoporous Ti‐MOFs Based NH_2_‐MIL‐125(Ti)@ZnIn_2_S_4_/CdS Hierarchical Tandem Heterojunctions With Improved Charge Separation and Enhanced Solar‐Driven Photocatalytic Performance,” Applied Catalysis B: Environmental 262 (2020): 118202.

[114] G. Yadav and M. Ahmaruzzaman , “ZnIn_2_S_4_ and ZnIn_2_S_4_ Based Advanced Hybrid Materials: Structure, Morphology and Applications in Environment and Energy,” Inorganic Chemistry Communications 138 (2022): 109288.

[115] Q. Zhang , H. Gu , X. Wang , et al., “Robust Hollow Tubular ZnIn_2_S_4_ Modified With Embedded Metal‐Organic‐Framework‐Layers: Extraordinarily High Photocatalytic Hydrogen Evolution Activity Under Simulated and Real Sunlight Irradiation,” Applied Catalysis B: Environmental 298 (2021): 120632.

[116] J. Zhou , D. Chen , L. Bai , L. Qin , X. Sun , and Y. Huang , “Decoration of WS_2_ as an Effective Noble‐Metal Free Cocatalyst on ZnIn_2_S_4_ for Enhanced Visible Light Photocatalytic Hydrogen Evolution,” International Journal of Hydrogen Energy 43 (2018): 18261–18269.

[117] S. Liu , X. Zhou , C. Yang , C. Wei , and Y. Hu , “Cu Atoms on UiO‐66‐NH_2_/ZnIn_2_S_4_ Nanosheets Enhance Photocatalytic Performance for Recovering Hydrogen Energy From Organic Wastewater Treatment,” Applied Catalysis B: Environmental 330 (2023): 122572.

[118] S. Mao , J.‐W. Shi , G. Sun , et al., “Au nanodots@thiol‐UiO66@ ZnIn_2_S_4_ Nanosheets With Significantly Enhanced Visible‐Light Photocatalytic H_2_ Evolution: The Effect of Different Au Positions on the Transfer of Electron‐Hole Pairs,” Applied Catalysis B: Environmental 282 (2021): 119550.

[119] X. Peng , L. Ye , Y. Ding , L. Yi , C. Zhang , and Z. Wen , “Nanohybrid Photocatalysts With ZnIn_2_S_4_ Nanosheets Encapsulated UiO‐66 Octahedral Nanoparticles for Visible‐Light‐Driven Hydrogen Generation,” Applied Catalysis B: Environmental 260 (2020): 118152.

[120] Z. Jin , H. Gong , and H. Li , “Visible‐Light‐Driven Two Dimensional Metal‐Organic Framework Modified Manganese Cadmium Sulfide for Efficient Photocatalytic Hydrogen Evolution,” Journal of Colloid and Interface Science 603 (2021): 344–355. 10.1016/j.jcis.2021.06.111 34197984

[121] J. Fan , D. Wu , X. Deng , Y. Zhao , C. Liu , and Q. Liang , “Carbon Dots as an Electron Acceptor in the ZnIn_2_S_4_@MIL‐88A Heterojunction for Enhanced Visible‐Light‐Driven Photocatalytic Hydrogen Evolution,” Langmuir 39 (2023): 12467–12475. 10.1021/acs.langmuir.3c01680 37620251

[122] Q. Chen , J. Li , L. Cheng , and H. Liu , “Construction of CdLa_2_S_4_/MIL‐88A(Fe) Heterojunctions for Enhanced Photocatalytic H_2_‐Evolution Activity via a Direct Z‐Scheme Electron Transfer,” Chemical Engineering Journal 379 (2020): 122389.

[123] P. Zhu , C. Feng , Q. Liang , M. Zhou , Z. Li , and S. Xu , “Cu‐MOF Modified Cd_0.5_Zn_0.5_S Nanoparticles to Form S‐Scheme Heterojunction for Efficient Photocatalytic H_2_ Evolution,” Ceramics International 49 (2023): 20706–20714.

[124] P. Jin , L. Wang , X. Ma , et al., “Construction of Hierarchical ZnIn_2_S_4_@PCN‐224 Heterojunction for Boosting Photocatalytic Performance in Hydrogen Production and Degradation of Tetracycline Hydrochloride,” Applied Catalysis B: Environmental 284 (2021): 119762.

[125] H. Chen , J. Wu , Y. Zhu , et al., “Cu‐MOF Modified ZnIn_2_S_4_ Nanosheet Composite Catalyst for Photocatalytic Hydrogen Production,” Renewable Energy 228 (2024): 120672.

[126] C. Zhao , Y. Zhang , H. Jiang , et al., “Combined Effects of Octahedron NH_2_‐UiO‐66 and Flowerlike ZnIn_2_S_4_ Microspheres for Photocatalytic Dye Degradation and Hydrogen Evolution Under Visible Light,” The Journal of Physical Chemistry C 123 (2019): 18037–18049.

[127] B. Deng , Q. Chen , Y. Liu , et al., “Quasi‐Type‐II Cu‐in‐Zn‐S/Ni‐MOF Heterostructure With Prolonged Carrier Lifetime for Photocatalytic Hydrogen Production,” Journal of Colloid and Interface Science 662 (2024): 1016–1025. 10.1016/j.jcis.2024.02.095 38387363

[128] J. Li , X. Li , Z. Yin , X. Wang , H. Ma , and L. Wang , “Synergetic Effect of Facet Junction and Specific Facet Activation of ZnFe_2_O_4_ Nanoparticles on Photocatalytic Activity Improvement,” ACS Applied Materials & Interfaces 11 (2019): 29004–29013. 10.1021/acsami.9b11836 31314495

[129] A. Adewuyi , “Ferrite Doped Metal–organic Framework: Novel Material for Photocatalytic Degradation of Antibiotics in the Polluted Water System–a Review,” Environmental Nanotechnology, Monitoring & Management 20 (2023): 100829.

[130] B. Alqassem , F. Banat , G. Palmisano , and M. A. Haija , “Emerging Trends of Ferrite‐Based Nanomaterials as Photocatalysts for Environmental Remediation: A Review and Synthetic Perspective,” Sustainable Materials and Technologies 40 (2024): e00961.

[131] S. B. Bagherzadeh , M. Kazemeini , and N. M. Mahmoodi , “A Study of the DR23 Dye Photocatalytic Degradation Utilizing a Magnetic Hybrid Nanocomposite of MIL‐53 (Fe)/CoFe_2_O_4_: Facile Synthesis and Kinetic Investigations,” Journal of Molecular Liquids 301 (2020): 112427.

[132] V. H. Nguyen , L. G. Bach , Q. T. P. Bui , et al., “Composite Photocatalysts Containing MIL‐53 (Fe) as a Heterogeneous Photo‐Fenton Catalyst for the Decolorization of Rhodamine B Under Visible Light Irradiation,” Journal of Environmental Chemical Engineering 6 (2018): 7434–7441.

[133] T. K. Vo and J. Kim , “Facile Synthesis of Magnetic Framework Composite MgFe_2_O_4_@UiO‐66 (Zr) and Its Applications in the Adsorption–photocatalytic Degradation of Tetracycline,” Environmental Science and Pollution Research 28 (2021): 68261–68275. 10.1007/s11356-021-15423-y 34268686

[134] M. Azqandi , K. Nateq , M. Amarzadeh , et al., “Intensified Photo‐Decontamination of Tetracycline Antibiotic by S‐Scheme Spinel Manganese Ferrite‐Grafted ZIF‐8 Heterojunction Photocatalyst: Mechanism Conception, Degradation Pathway and DFT Studies,” Journal of Environmental Chemical Engineering 12 (2024): 112875.

[135] F. Sharmin and M. Basith , “Simple Low Temperature Technique to Synthesize Sillenite Bismuth Ferrite With Promising Photocatalytic Performance,” ACS Omega 7 (2022): 34901–34911. 10.1021/acsomega.2c03457 PMC9535739 36211068

[136] A. Kaur , M. Kaur , V. Singh , and P. Vyas , “Nanocomposites of Ferrites With TiO_2_, SiO_2_ and Carbon Quantum Dots as Photocatalysts for Degradation of Organic Pollutants and Microbes,” Magnetochemistry (Basel, Switzerland) 9 (2023): 127.

[137] Y. Si , Y. Li , J. Zou , X. Xiong , X. Zeng , and J. Zhou , “Photocatalytic Performance of a Novel MOF/BiFeO3 Composite,” Materials 10 (2017): 1161. 10.3390/ma10101161 PMC5666967 28994708

[138] M. Jamshaid , S. Jabeen , M. Mohany , et al., “Synergistic Photodegradation of Moxifloxacin With BiFeO_3_@ZIF‐67 Heterojunction Co‐Catalyst,” Journal of Molecular Structure 1318 (2024): 139332.

[139] Q. Xiang , Z. Yu , P. Wang , et al., “Construction of Z‐Scheme N‐Doped BiFeO_3_/NH_2_‐MIL‐53 (Fe) With the Synergy of Hydrogen Peroxide and Visible‐Light‐Driven Photo‐Fenton Degradation of Organic Contaminants,” Colloids and Surfaces A: Physicochemical and Engineering Aspects 654 (2022): 130112.

[140] J. Schneider , M. Matsuoka , M. Takeuchi , et al., “Understanding TiO_2_ Photocatalysis: Mechanisms and Materials,” Chemical Reviews 114 (2014): 9919–9986. 10.1021/cr5001892 25234429

[141] H. Liu , X. Chang , X. Liu , G. Li , W. Zhang , and T. An , “Boosting the Photocatalytic Degradation of Ethyl Acetate by a Z‐Scheme Au–TiO_2_@ NH_2_‐UiO‐66 Heterojunction With Ultrafine Au as an Electron Mediator,” Environmental Science: Nano 8 (2021): 2542–2553.

[142] J. Hao , B. Li , J. Dai , et al., “Degradation of Thiosulfate Ions in Sodium Aluminate Solution Photocatalyzed by Co‐TiO_2_@ZIF‐8,” Minerals Engineering 218 (2024): 109038.

[143] B. Yan , L. Zhang , Z. Tang , M. Al‐Mamun , H. Zhao , and X. Su , “Palladium‐Decorated Hierarchical Titania Constructed From the Metal‐Organic Frameworks NH_2_‐MIL‐125 (Ti) as a Robust Photocatalyst for Hydrogen Evolution,” Applied Catalysis B: Environmental 218 (2017): 743–750.

[144] N. Ahmadpour , M. H. Sayadi , and S. Homaeigohar , “A Hierarchical Ca/TiO_2_/NH_2_‐MIL‐125 Nanocomposite Photocatalyst for Solar Visible Light Induced Photodegradation of Organic Dye Pollutants in Water,” RSC Advances 10 (2020): 29808–29820. 10.1039/d0ra05192f PMC9056284 35518266

[145] Z. H. Al‐Azri , W.‐T. Chen , A. Chan , et al., “The Roles of Metal Co‐Catalysts and Reaction Media in Photocatalytic Hydrogen Production: Performance Evaluation of M/TiO_2_ Photocatalysts (M = Pd, Pt, Au) in Different Alcohol–water Mixtures,” Journal of Catalysis 329 (2015): 355–367.

[146] X. Zhang , Y. L. Chen , R.‐S. Liu , and D. P. Tsai , ”Plasmonic Photocatalysis,“ Reports on Progress in Physics 76 (2013): 046401. 10.1088/0034-4885/76/4/046401 23455654

[147] F. Fazlali , A. Hajian , A. Afkhami , and H. Bagheri , “A Superficial Approach for Fabricating Unique Ternary AgI@TiO_2_/Zr‐MOF Composites: An Excellent Interfacial With Improved Photocatalytic Light‐Responsive Under Visible Light,” Journal of Photochemistry and Photobiology A: Chemistry 400 (2020): 112717.

[148] N. Mohaghegh , M. Faraji , and A. Abedini , “Highly Efficient Multifunctional Ag/TiO_2_ Nanotubes/Ti Plate Coated With MIL‐88B (Fe) as a Photocatalyst, Adsorbent, and Disinfectant in Water Treatment,” Applied Physics A 125 (2019): 1–10.

[149] R. Abazari , A. Morsali , and D. P. Dubal , “An Advanced Composite With Ultrafast Photocatalytic Performance for the Degradation of Antibiotics by Natural Sunlight Without Oxidizing the Source Over TMU‐5@Ni–Ti LDH: Mechanistic Insight and Toxicity Assessment,” Inorganic Chemistry Frontiers 7 (2020): 2287–2304.

[150] I. Batool , S. Aroob , F. Anwar , et al., “Synergistic Effect of NiAl‐Layered Double Hydroxide and Cu‐MOF for the Enhanced Photocatalytic Degradation of Methyl Orange and Antibacterial Properties,” Catalysts 14 (2024): 719.

[151] G. Fan , F. Li , D. G. Evans , and X. Duan , “Catalytic Applications of Layered Double Hydroxides: Recent Advances and Perspectives,” Chemical Society Reviews 43 (2014): 7040–7066. 10.1039/c4cs00160e 25001024

[152] I. Maseeh , F. Anwar , S. Aroob , et al., “Multifunctional MgAl LDH/Zn‐MOF S‐Scheme Heterojunction: Efficient Hydrogen Production, Methyl Red Removal, and CO_2_ Adsorption,” Materials Advances 5 (2024): 5080–5095.

[153] C. Zhou , Y. Yang , G. Wu , M. Mu , and X. Yin , “2D Cu‐FeTCPP MOF Assembled on ZnTi‐LDH to Construct 2D/2D Direct Z‐Scheme Heterojunction for Enhanced Photocatalytic CO_2_ Reduction,” Solar Energy 253 (2023): 480–490.

[154] Q. Shi , G. Zhang , M. Cheng , et al., “Dazzling Construction and Derivation of Metal–organic Frameworks With Layered Double Hydroxide Substrates in Environmental Field: Insight Into Synthesis, Performance and Mechanism,” Applied Materials Today 38 (2024): 102192.

[155] C. S. Vennapoosa , S. Varangane , S. Gonuguntla , B. M. Abraham , M. Ahmadipour , and U. Pal , “S‐Scheme ZIF‐67/CuFe‐LDH Heterojunction for High‐Performance Photocatalytic H_2_ Evolution and CO_2_ to MeOH Production,” Inorganic Chemistry 62 (2023): 16451–16463. 10.1021/acs.inorgchem.3c02126 37737088

[156] L. Liu , S. Li , Y. An , et al., “Hybridization of Nanodiamond and CuFe‐LDH as Heterogeneous Photoactivator for Visible‐Light Driven Photo‐Fenton Reaction: Photocatalytic Activity and Mechanism,” Catalysts 9 (2019): 118.

[157] A. Dhakshinamoorthy , Z. Li , S. Yang , and H. Garcia , “Metal–organic Framework Heterojunctions for Photocatalysis,” Chemical Society Reviews 53 (2024): 3002–3035, 10.1039/D3CS00205E. 38353930

[158] D. Sun , J. Li , T. Shen , S. An , B. Qi , and Y.‐F. Song , “In Situ Construction of MIL‐100@NiMn‐LDH Hierarchical Architectures for Highly Selective Photoreduction of CO_2_ to CH_4_ ,” ACS Applied Materials & Interfaces 14 (2022): 16369–16378. 10.1021/acsami.2c02888 35354278

[159] Z. Jin , Y. Li , and Q. Ma , “CoAl LDH@Ni‐MOF‐74 S‐Scheme Heterojunction for Efficient Hydrogen Evolution,” Transactions of Tianjin University 27 (2021): 127–138.

[160] S.‐Y. Xu , J.‐Q. Ye , M.‐Y. Ren , J.‐F. Qian , M.‐Y. He , and Q. Chen , “An In‐Situ Growth of NiAl‐LDH on MIL‐53 (Fe) S‐Scheme Heterojunction With Boosted Carrier Separation for Enhanced Photocatalytic Hydrogen Evolution,” Journal of Environmental Chemical Engineering 13 (2025): 115857.

[161] J. Hua , S. Feng , C. Ma , et al., ”Mof‐199 Loaded Znal‐Ldh for Enhancing S‐Scheme Photocatalytic Reduction of Co_2_ .“ SSRN, 10.2139/ssrn.4805058.

[162] D. Wu , F. He , Y. Dai , et al., “A Heterostructured ZnAl‐LDH@ ZIF‐8 Hybrid as a Bifunctional Photocatalyst/Adsorbent for CO_2_ Reduction Under Visible Light Irradiation,” Chemical Engineering Journal 446 (2022): 137003.

[163] W. Xiong , W. Ouyang , M. Li , et al., “Highly Stable Lead‐Free Perovskite Cs_2_AgBiBr_6_/UiO‐66 Z‐Scheme Heterostructures With Enhanced Photocatalytic Activity for CO_2_ Photoreduction and Organic Dyes Degradation,” Applied Surface Science 644 (2024): 158807.

[164] Y. Xi , X. Zhang , Y. Shen , et al., “Aspect Ratio Dependent Photocatalytic Enhancement of CsPbBr_3_ in CO_2_ Reduction With Two‐Dimensional Metal Organic Framework as a Cocatalyst,” Applied Catalysis B: Environmental 297 (2021): 120411.

[165] Z.‐C. Kong , J.‐F. Liao , Y.‐J. Dong , et al., “Core@Shell CsPbBr_3_@ Zeolitic Imidazolate Framework Nanocomposite for Efficient Photocatalytic CO_2_ Reduction,” ACS Energy Letters 3 (2018): 2656–2662.

[166] S. Wan , M. Ou , Q. Zhong , and X. Wang , “Perovskite‐Type CsPbBr_3_ Quantum Dots/UiO‐66 (NH_2_) Nanojunction as Efficient Visible‐Light‐Driven Photocatalyst for CO_2_ Reduction,” Chemical Engineering Journal 358 (2019): 1287–1295.

[167] L. Ding , F. Bai , B. Borjigin , Y. Li , H. Li , and X. Wang , “Embedding Cs_2_AgBiBr_6_ QDs Into Ce‐UiO‐66‐H to In Situ Construct a Novel Bifunctional Material for Capturing and Photocatalytic Reduction of CO_2_ ,” Chemical Engineering Journal 446 (2022): 137102.

[168] A. Asadi , N. Khosroshahi , and V. Safarifard , “Engineering Water‐Stable Metal Halide Perovskite/MOF‐808 Heterojunction Through Mechanochemical Method: S‐Scheme Photocatalytic System for Multifunctional Applications,” Materials Science in Semiconductor Processing 165 (2023): 107707.

[169] S. Sharma , N. Jacob , G. K. Grandhi , et al., “Synergistic Metal Halide Perovskite@Metal‐Organic Framework Hybrids for Photocatalytic CO_2_ Reduction,” iScience 27 (2024): 110924. 10.1016/j.isci.2024.110924 PMC11439556 39346676

[170] A. Afzalinia , M. Mirzaee , and M. A. Amani , “Design of an S‐Scheme Photo‐Catalyst Utilizing a Cu‐Doped Perovskite and MOF‐5 for Simultaneous Degradation of Organic Pollutants Under LED Light Irradiation: Application of EXRSM Method for Spectra Separation and BBD‐RSM Modeling,” Spectrochimica Acta Part A: Molecular and Biomolecular Spectroscopy 287 (2023): 122116, 10.1016/j.saa.2022.122116. 36403539

[171] Z. Xia , B. Liu , Y. Xiao , W. Hu , M. Deng , and C. Lü , “Integrating Hybrid Perovskite Nanocrystals Into Metal–Organic Framework as Efficient S‐Scheme Heterojunction Photocatalyst for Synergistically Boosting Controlled Radical Photopolymerization Under 980 nm NIR Light,” ACS Applied Materials & Interfaces 15 (2023): 57119–57133, 10.1021/acsami.3c13496. 38032100

[172] B. Liu , Z. Xia , W. Hu , S. Li , and C. Lü , “Efficient Oxygen‐Accelerated Near‐Infrared Photoinduced Atom Transfer Radical Polymerization Mediated With S‐Scheme Heterojunction Photocatalyst Composed of Lead‐Free Halide Perovskite Encapsulated Into Metal–Organic Framework,” Macromolecules 57 (2024): 10283–10296, 10.1021/acs.macromol.4c01764.

[173] W. Wang , B. Ibarlucea , C. Huang , et al., “Multi‐Metallic MOF Based Composites for Environmental Applications: Synergizing Metal Centers and Interactions,” Nanoscale Horizons 9 (2024): 1432–1474. 10.1039/d4nh00140k 38984482

[174] M. Kaur , S. K. Mehta , P. Devi , and S. K. Kansal , “Bi_2_WO_6_/NH_2_‐MIL‐88B(Fe) Heterostructure: An Efficient Sunlight Driven Photocatalyst for the Degradation of Antibiotic Tetracycline in Aqueous Medium,” Advanced Powder Technology 32 (2021): 4788–4804.

[175] Y. Tan , Y. Zhou , Y. Deng , et al., “A Novel UiO‐66‐NH_2_/Bi_2_WO_6_ Composite With Enhanced Pollutant Photodegradation Through Interface Charge Transfer,” Colloids and Surfaces A: Physicochemical and Engineering Aspects 622 (2021): 126699.

[176] G. Wu , Q. Liu , L. Ma , et al., “The Promoted Organic Pollutant and Visible‐Light‐Driven Photocatalytic Degradation Efficiency of MIL‐101(Fe)/Bi_2_WO_6_ Z‐Scheme Heterojunction Assisting and Mechanism,” Colloids and Surfaces A: Physicochemical and Engineering Aspects 656 (2023): 130413.

[177] S. Yin , Y. Chen , C. Gao , et al., “In‐Situ Preparation of MIL‐125 (Ti)/Bi_2_WO_6_ Photocatalyst With Accelerating Charge Carriers for the Photodegradation of Tetracycline Hydrochloride,” Journal of Photochemistry and Photobiology A: Chemistry 387 (2020): 112149.

[178] Q. Wang , X. Qian , H. Xu , G. He , and H. Chen , “Enriched Surface Oxygen Vacancies of Bi_2_WO_6_/NH_2_‐MIL‐68 (in) Z‐Scheme Heterojunction With Boosted Visible‐Light Photocatalytic Degradation for Levofloxacin: Performance, Degradation Pathway and Mechanism Insight,” Separation and Purification Technology 306 (2023): 122577.

[179] Y. Xia , S.‐K. Shang , X.‐R. Zeng , J. Zhou , and Y.‐Y. Li , “A Novel Bi_2_MoO_6_/ZIF‐8 Composite for Enhanced Visible Light Photocatalytic Activity,” Nanomaterials 9 (2019): 545. 10.3390/nano9040545 PMC6523258 30987268

[180] Z. Sha , J. Sun , H. S. O. Chan , S. Jaenicke , and J. Wu , “Bismuth Tungstate Incorporated Zirconium Metal–organic Framework Composite With Enhanced Visible‐Light Photocatalytic Performance,” RSC Advances 4 (2014): 64977–64984.

[181] L. Yang , Y. Xin , C. Yao , and Y. Miao , “In Situ Preparation of Bi_2_WO_6_/CAU‐17 Photocatalyst With Excellent Photocatalytic Activity for Dye Degradation,” Journal of Materials Science: Materials in Electronics 32 (2021): 13382–13395.

[182] D. Wang , H. Li , Q. Han , W. Jiang , C. Liu , and G. Che , “Optimized Design of BiVO_4_/NH_2_‐MIL‐53(Fe) Heterostructure for Enhanced Photocatalytic Degradation of Methylene Blue and Ciprofloxacin Under Visible Light,” Journal of Physics and Chemistry of Solids 154 (2021): 110027.

[183] H. E. Emam , H. B. Ahmed , E. Gomaa , M. H. Helal , and R. M. Abdelhameed , “Doping of Silver Vanadate and Silver Tungstate Nanoparticles for Enhancement the Photocatalytic Activity of MIL‐125‐NH_2_ in Dye Degradation,” Journal of Photochemistry and Photobiology A: Chemistry 383 (2019): 111986.

[184] Y.‐H. Si , Y.‐Y. Li , Y. Xia , et al., “Fabrication of Novel ZIF‐8@BiVO_4_ Composite with Enhanced Photocatalytic Performance,” Crystals 8 (2018): 432.

[185] S. Mosleh , M. Rahimi , M. Ghaedi , and K. Dashtian , “HKUST‐1‐MOF–BiVO_4_ Hybrid as a New Sonophotocatalyst for Simultaneous Degradation of Disulfine Blue and Rose Bengal Dyes: Optimization and Statistical Modelling,” RSC Advances 6 (2016): 61516–61527.

[186] J. Chen , F. Chao , X. Mu , et al., “ZnIn_2_S_4_/UiO‐66‐(SH)_2_ Composites as Efficient Visible‐Light Photocatalyst for RhB Degradation,” Inorganic Chemistry Communications 102 (2019): 25–29.

[187] A. Faraji , N. Mehrdadi , N. M. Mahmoodi , M. Baghdadi , and A. Pardakhti , “Enhanced Photocatalytic Activity by Synergic Action of ZIF‐8 and NiFe_2_O_4_ Under Visible Light Irradiation,” Journal of Molecular Structure 1223 (2021): 129028.

[188] H. Tian , J. Peng , T. Lv , C. Sun , and H. He , “Preparation and Performance Study of MgFe_2_O_4_/Metal–organic Framework Composite for Rapid Removal of Organic Dyes From Water,” Journal of Solid State Chemistry 257 (2018): 40–48.

[189] L. R. Rad , M. Irani , and M. Anbia , “Electrocoagulation‐Photocatalysis Sequential Combined Method for the Removal of Anticancer Drugs From Pharmaceutical Wastewater Using NH_2_‐MIL‐125 (Ti)/Cobalt Ferrite Nanorods Composite Photocatalyst,” Journal of Environmental Chemical Engineering 12 (2024): 113302.

[190] Y. Luo , G. Shi , S. Yu , et al., “Novel MIL‐88B (Fe)/ZnTi‐LDH High‐Low Junctions for Adsorption and Photodegradation of Tetracycline: Characteristics, Performance, and Mechanisms,” Chemical Engineering Journal 473 (2023): 145198.

[191] G. R. Jarre‐Vera , B. F. Rivadeneira‐Mendoza , K. J. Fernández‐Andrade , et al., “Structured Design of a Hydrochar‐Supported LDH/MOF Composite for Improved Photocatalytic Applications,” Results in Engineering 24 (2024): 103424.

[192] A. Bhuyan and M. Ahmaruzzaman , “Ultrasonic‐Assisted Synthesis of Highly Efficient and Robust Metal Oxide QDs Immobilized‐MOF‐5/Ni‐Co‐LDH Photocatalyst for Sunlight‐Mediated Degradation of Multiple Toxic Dyes,” Journal of Alloys and Compounds 972 (2024): 172781.

[193] W. Cai , X. Yu , Y. Cao , et al., “Electron‐Coupled Enhanced Interfacial Interaction of Ce‐MOF/Bi_2_MoO_6_ Heterostructure for Boosted Photoreduction CO_2_ ,” Journal of Environmental Chemical Engineering 10 (2022): 107461.

[194] Z. Guo , W. Zheng , X. Yan , et al., “Ionic Liquid Tuning Nanocage Size of MOFs Through a Two‐Step Adsorption/Infiltration Strategy for Enhanced Gas Screening of Mixed‐Matrix Membranes,” Journal of Membrane Science 605 (2020): 118101.

[195] Y. Zhou , X. Zhao , Q. Liang , et al., “In Situ Growth of CdIn_2_S_4_ on NH_2_‐MIL‐125 as Efficient Photocatalysts for H_2_ Production Under Visible‐Light Irradiation,” Journal of Physics and Chemistry of Solids 173 (2023): 111096.

[196] H. Liu , J. Zhang , and D. Ao , “Construction of Heterostructured ZnIn_2_S_4_@NH_2_‐MIL‐125 (Ti) Nanocomposites for Visible‐Light‐Driven H_2_ Production,” Applied Catalysis B: Environmental 221 (2018): 433–442.

[197] J. Cai , B. Liu , S. Zhang , et al., “ZnIn_2_S_4_/MOF S‐Scheme Photocatalyst for H_2_ Production and Its Femtosecond Transient Absorption Mechanism,” Journal of Materials Science & Technology 197 (2024): 183–193.

[198] L. Cui , X. Zou , Y. Liu , et al., “Dramatic Enhancement of Photocatalytic H_2_ Evolution Over Hydrolyzed MOF‐5 Coupled Zn_0.2_Cd_0.8_S Heterojunction,” Journal of Colloid and Interface Science 577 (2020): 233–241. 10.1016/j.jcis.2020.05.023 32485407

[199] Y. Bi , K. Xu , Y. Wang , et al., “Efficient Metal–organic Framework‐Based Dual Co‐Catalysts System Assist CdS for Hydrogen Production From Photolysis of Water,” Journal of Colloid and Interface Science 661 (2024): 501–511. 10.1016/j.jcis.2024.01.158 38308890

[200] X. Zhou , S. Liu , C. Yang , J. Qin , and Y. Hu , “Photocatalytic Hydrogen Energy Recovery From Sulfide‐Containing Wastewater Using Thiol‐UiO‐66 Modified Mn_0.5_Cd_0.5_S Nanocomposites,” Separation and Purification Technology 316 (2023): 123772.

[201] Y. Guo , J. Li , X. Yang , Y. Lou , and J. Chen , “Zn0.5Cd0. 5S/MIL‐125‐NH_2_ (Ti) Nanocomposites: Highly Efficient and Stable Photocatalyst for Hydrogen Production Under Visible Light,” Inorganic Chemistry Communications 112 (2020): 107714.

[202] Y. Wang , H. Jin , Y. Li , J. Fang , and C. Chen , “Ce‐Based Organic Framework Enhanced the Hydrogen Evolution Ability of ZnCdS Photocatalyst,” International Journal of Hydrogen Energy 47 (2022): 962–970.

[203] Y. Cao , G. Wang , H. Liu , Y. Li , Z. Jin , and Q. Ma , “Regular Octahedron Cu‐MOFs Modifies Mn_0.05_Cd_0.95_S Nanoparticles to Form a S‐Scheme Heterojunction for Photocatalytic Hydrogen Evolution,” International Journal of Hydrogen Energy 46 (2021): 7230–7240.

[204] Y. Wang , Y. Zhang , Z. Jiang , et al., “Controlled Fabrication and Enhanced Visible‐Light Photocatalytic Hydrogen Production of Au@CdS/MIL‐101 Heterostructure,” Applied Catalysis B: Environmental 185 (2016): 307–314.

[205] M. A. A. El‐Khair , A. G. Al‐Gamal , K. I. Kabel , W. S. Gado , and A. S. Morshedy , “Harvesting the Synergistic Effect of CuFe_2_O_4_@Ni‐MOF Nanomagnetic Photocatalyst for Enhanced Visible Light‐Driven Green Hydrogen Production,” International Journal of Hydrogen Energy 101 (2025): 280–294.

[206] M. Shanmugam , N. Agamendran , and K. Sekar , “Efficient Charge Transfer of Pn Heterojunction UiO‐66‐NH_2_/CuFe_2_O_4_ Composite for Photocatalytic Hydrogen Production,” Catalysts 14 (2024): 341.

[207] G. Wang , Y. Li , L. Xu , Z. Jin , and Y. Wang , “Facile Synthesis of Difunctional NiV LDH@ZIF‐67 Pn Junction: Serve as Prominent Photocatalyst for Hydrogen Evolution and Supercapacitor Electrode as Well,” Renewable Energy 162 (2020): 535–549.

[208] S. Sk , A. Jamma , D. S. Gavali , et al., “Modulated Ultrathin NiCo‐LDH Nanosheet‐Decorated Zr^3+^‐Rich Defective NH_2_–UiO‐66 Nanostructure for Efficient Photocatalytic Hydrogen Evolution,” ACS Applied Materials & Interfaces 15 (2023): 55822–55836. 10.1021/acsami.3c13009 37994833

[209] K. Wang , S. Liu , Y. Li , G. Wang , M. Yang , and Z. Jin , “Phosphorus ZIF‐67@NiAl LDH S‐Scheme Heterojunction for Efficient Photocatalytic Hydrogen Production,” Applied Surface Science 601 (2022): 154174.

[210] M. Sohail , H. Kim , and T. W. Kim , “Enhanced Photocatalytic Performance of a Ti‐Based Metal‐Organic Framework for Hydrogen Production: Hybridization With ZnCr‐LDH Nanosheets,” Scientific Reports 9 (2019): 7584. 10.1038/s41598-019-44008-6 PMC6527569 31110219

[211] L. Zhang , J. Qiu , D. Dai , Y. Zhou , X. Liu , and J. Yao , “Cr‐Metal‐organic Framework Coordination with ZnIn_2_S_4_ Nanosheets for Photocatalytic Reduction of Cr(VI),” Journal of Cleaner Production 341 (2022): 130891.

[212] R. Yuan , J. Qiu , C. Yue , et al., “Self‐Assembled Hierarchical and Bifunctional MIL‐88A (Fe)@ZnIn_2_S_4_ Heterostructure as a Reusable Sunlight‐Driven Photocatalyst for Highly Efficient Water Purification,” Chemical Engineering Journal 401 (2020): 126020.

[213] H. Peng , L. Wang , H. Li , and X. Zheng , “Visible‐Light Photoreduction of Cr(VI) Over CdIn_2_S_4_/MOF‐808 Composites,” Surfaces and Interfaces 51 (2024): 104567.

[214] N. Khosroshahi , M. D. Goudarzi , M. E. Gilvan , and V. Safarifard , “Collocation of MnFe_2_O_4_ and UiO‐66‐NH_2_: An Efficient and Reusable Nanocatalyst for Achieving High‐Performance in Hexavalent Chromium Reduction,” Journal of Molecular Structure 1263 (2022): 132994.

[215] J. He , J. Hu , Y. Hu , et al., “Hierarchical S‐Scheme Heterostructure of CdIn_2_S_4_@UiO‐66‐NH_2_ Toward Synchronously Boosting Photocatalytic Removal of Cr(VI) and Tetracycline,” Inorganic Chemistry 61 (2022): 19961–19973. 10.1021/acs.inorgchem.2c03240 36417671

[216] N. Khosroshahi , M. Bakhtian , and V. Safarifard , “Mechanochemical Synthesis of Ferrite/MOF Nanocomposite: Efficient Photocatalyst for the Removal of Meropenem and Hexavalent Chromium From Water,” Journal of Photochemistry and Photobiology A: Chemistry 431 (2022): 114033.

[217] E.‐Z. Deng , Y.‐Z. Fan , H.‐P. Wang , Y. Li , C. Peng , and J. Liu , “Engineering a Z‐Scheme Heterostructure on ZnIn_2_S_4_@NH_2_‐MIL‐125 Composites for Boosting the Photocatalytic Performance,” Inorganic Chemistry 63 (2024): 1449–1461. 10.1021/acs.inorgchem.3c03968 38221879

[218] H. Haroon and K. Majid , “Enhanced d–d Transitions in HKUST/Bi_2_WO_6_ Nanocomposite Mediated Visible‐Light Driven Selective Conversion of Benzyl Alcohol to Benzaldehyde,” New Journal of Chemistry 44 (2020): 18380–18388.

[219] Q. Zhang , J.‐B. Liu , L. Chen , et al., “An Etching and Re‐Growth Method for the Synthesis of Bismuth Ferrite/MIL‐53 (Fe) Nanocomposite as Efficient Photocatalyst for Selective Oxidation of Aromatic Alcohols,” Applied Catalysis B: Environmental 264 (2020): 118529.

[220] X.‐Y. Lin , M.‐Y. Qi , Z.‐R. Tang , and Y.‐J. Xu , “Photochemical Dehydrogenation of N‐Heterocycles Over MOF‐Supported CdS Nanoparticles With Nickel Modification,” Applied Catalysis B: Environmental 317 (2022): 121708.

[221] J. Yang , X. He , J. Dai , R. Tian , and D. Yuan , “Photo‐Assisted Enhancement Performance for Rapid Detoxification of Chemical Warfare Agent Simulants Over Versatile ZnIn_2_S_4_/UiO‐66‐NH_2_ Nanocomposite Catalysts,” Journal of Hazardous Materials 417 (2021): 126056. 10.1016/j.jhazmat.2021.126056 33992917

[222] R. Bariki , S. K. Pradhan , S. Panda , S. K. Nayak , A. R. Pati , and B. Mishra , “Hierarchical UiO‐66−NH_2_/CuInS_2_ S‐Scheme Photocatalyst With Controlled Topology for Enhanced Photocatalytic N_2_ Fixation and H_2_O_2_ Production,” Langmuir 39 (2023): 7707–7722. 10.1021/acs.langmuir.3c00519 37212348

[223] M. Z. Hussain , Z. Yang , B. Van Der Linden , et al., “MOF‐Derived Multi‐heterostructured Composites for Enhanced Photocatalytic Hydrogen Evolution: Deciphering the Roles of Different Components,” Energy & Fuels 36 (2022): 12212–12225.

[224] K. Balu , B. Avula , M. Durai , et al., “Fabrication of Bi_2_O_3_/Bismuth Titanates Modified With Metal–organic Framework‐in_2_S_3_/CdIn_2_S_4_ Materials for Electrocatalytic H_2_ Production and Its Photoactivity,” Langmuir 39 (2023): 15055–15066. 10.1021/acs.langmuir.3c02031 PMC10601539 37842923

[225] J. Mou , Y. Xu , D. Zhong , et al., “Efficient Visible‐Light‐Responsive Photocatalytic Fuel Cell With a ZnIn_2_S_4_/UiO‐66/TiO_2_/Ti Photoanode for Simultaneous RhB Degradation and Electricity Generation,” Journal of Electronic Materials 51 (2022): 6121–6133.

[226] R. Bariki , S. K. Pradhan , S. Panda , et al., “In‐Situ Synthesis of Structurally Oriented Hierarchical UiO‐66–NH_2_/CdIn_2_S_4_/CaIn_2_S_4_ Heterostructure With Dual S‐Scheme Engineering for Photocatalytic Renewable H_2_ Production and Asulam Degradation,” Separation and Purification Technology 314 (2023): 123558.

[227] S. Liu , X. Jiang , G. I. Waterhouse , Z.‐M. Zhang , and L.‐m. Yu , “A Novel Z‐Scheme NH_2_‐MIL‐125 (Ti)/Ti_3_C_2_ QDs/ZnIn_2_S_4_ Photocatalyst With Fast Interfacial Electron Transfer Properties for Visible Light‐Driven Antibiotic Degradation and Hydrogen Evolution,” Separation and Purification Technology 294 (2022): 121094.

[228] Y. Yang , Z. Xing , W. Kong , et al., “Metal–organic Framework (MOF)‐5/CuO@ZnIn_2_S_4_ Core–shell Z‐Scheme Tandem Heterojunctions for Improved Charge Separation and Enhanced Photocatalytic Performance,” Nanoscale 14 (2022): 14741–14749. 10.1039/d2nr03557j 36172834

[229] N. Mohaghegh , M. Tasviri , E. Rahimi , and M. Gholami , “Comparative Studies on Ag_3_PO_4_/BiPO_4_–metal‐Organic Framework–graphene‐Based Nanocomposites for Photocatalysis Application,” Applied Surface Science 351 (2015): 216–224.

[230] T. Zhou , G. Zhang , H. Zhang , et al., “Highly Efficient Visible‐Light‐Driven Photocatalytic Degradation of Rhodamine B by a Novel Z‐Scheme Ag_3_PO_4_/MIL‐101/NiFe_2_O_4_ Composite,” Catalysis Science & Technology 8 (2018): 2402–2416.

[231] S. Mosleh , M. Rahimi , M. Ghaedi , and K. Dashtian , “Sonophotocatalytic Degradation of Trypan Blue and Vesuvine Dyes in the Presence of Blue Light Active Photocatalyst of Ag_3_PO_4_/Bi_2_S_3_‐HKUST‐1‐MOF: Central Composite Optimization and Synergistic Effect Study,” Ultrasonics Sonochemistry 32 (2016): 387–397. 10.1016/j.ultsonch.2016.04.007 27150785

[232] M. Bakhtian , N. Khosroshahi , and V. Safarifard , “Efficient Removal of Inorganic and Organic Pollutants Over a NiCo_2_O_4_@MOF‐801@MIL88a Photocatalyst: The Significance of Ternary Heterojunction Engineering,” ACS Omega 7 (2022): 42901–42915. 10.1021/acsomega.2c05000 PMC9713798 36467958

[233] M. Hajiali , M. Farhadian , and S. Tangestaninejad , “Novel ZnO Nanorods/Bi_2_MoO_6_/MIL‐101 (Fe) Heterostructure Immobilized on FTO With Boosting Photocatalytic Activity for Tetracycline Degradation: Reaction Mechanism and Toxicity Assessment,” Applied Surface Science 602 (2022): 154389.

[234] S. G. Khasevani , and M. R. Gholami , “Synthesis of BiOI/ZnFe_2_O_4_ –Metal–Organic Framework and g‐C_3_N_4_ ‐Based Nanocomposites for Applications in Photocatalysis,” Industrial & Engineering Chemistry Research 58 (2019): 9806–9818.

[235] S. M. Flihh and S. H. Ammar , “Fabrication and Photocatalytic Degradation Activity of Core/Shell ZIF‐67@CoWO_4_@CoS Heterostructure Photocatalysts Under Visible Light,” Environmental Nanotechnology, Monitoring & Management 16 (2021): 100595.

[236] A. Wang , L. Zhang , X. Li , et al., “Synthesis of Ternary Ni_2_P@UiO‐66‐NH_2_/Zn_0.5_Cd_0.5_S Composite Materials With Significantly Improved Photocatalytic H_2_ Production Performance,” Chinese Journal of Catalysis 43 (2022): 1295–1305, 10.1016/S18722067(21)639128.

[237] Y. Pan , D. Li , and H. L. Jiang , “Sodium‐Doped C_3_N_4_/MOF Heterojunction Composites With Tunable Band Structures for Photocatalysis: Interplay Between Light Harvesting and Electron Transfer,” Chemistry–A European Journal 24 (2018): 18403–18407. 10.1002/chem.201803555 30156036

[238] L. Biswal , S. P. Tripathy , S. Dash , S. Das , S. Subudhi , and K. Parida , “Aggrandized Photocatalytic H_2_O_2_ and H_2_ Production by a TiO_2_/Ti_3_C_2_–TiC/Mixed Metal Ce–Zr MOF Composite: An Interfacial Engineered Solid‐State‐Mediator‐Based Z‐Scheme Heterostructure,” Materials Advances 5 (2024): 4452–4466.

[239] S. Mao , J.‐W. Shi , G. Sun , et al., “PdS Quantum Dots as a Hole Attractor Encapsulated Into the MOF@Cd_0.5_Zn_0.5_S Heterostructure for Boosting Photocatalytic Hydrogen Evolution Under Visible Light,” ACS Applied Materials & Interfaces 14 (2022): 48770–48779. 10.1021/acsami.2c15052 36259606

[240] F. Mu , Q. Cai , H. Hu , et al., “Construction of 3D Hierarchical Microarchitectures of Z‐Scheme UiO‐66‐(COOH)_2_/ZnIn_2_S_4_ Hybrid Decorated With Non‐Noble MoS_2_ Cocatalyst: A Highly Efficient Photocatalyst for Hydrogen Evolution and Cr(VI) Reduction,” Chemical Engineering Journal 384 (2020): 123352.

[241] S. Liu , X. Jiang , G. I. N. Waterhouse , Z.‐M. Zhang , and L.‐M. Yu , “Construction of Z‐Scheme Titanium‐MOF/Plasmonic Silver Nanoparticle/NiFe Layered Double Hydroxide Photocatalysts With Enhanced Dye and Antibiotic Degradation Activity Under Visible Light,” Separation and Purification Technology 278 (2021): 119525.

[242] L. Wang , Z. Zhang , Q. Han , et al., “Preparation of CdS‐P25/ZIF‐67 Composite Material and Its Photocatalytic CO_2_ Reduction Performance,” Applied Surface Science 584 (2022): 152645.

[243] L. Li , X. Yu , L. Xu , and Y. Zhao , “Fabrication of a Novel Type Visible‐Light‐Driven Heterojunction Photocatalyst: Metal‐Porphyrinic Metal Organic Framework Coupled With PW_12_/TiO_2_ ,” Chemical Engineering Journal 386 (2020): 123955.

[244] F. Wang , Y. T. Zhang , Y. Xu , et al., “Enhanced Photodegradation of Rhodamine B by Coupling Direct Solid‐State Z‐Scheme N‐K_2_Ti_4_O_9_/g‐C_3_N_4_ Heterojunction With High Adsorption Capacity of UiO‐66,” Journal of Environmental Chemical Engineering 4 (2016): 3364–3373.

[245] S. B. Bagherzadeh , M. Kazemeini , and N. M. Mahmoodi , “Preparation of Novel and Highly Active Magnetic Ternary Structures (metal‐Organic Framework/Cobalt Ferrite/Graphene Oxide) for Effective Visible‐Light‐Driven Photocatalytic and Photo‐Fenton‐Like Degradation of Organic Contaminants,” Journal of Colloid and Interface Science 602 (2021): 73–94. 10.1016/j.jcis.2021.05.181 34118607

[246] H. Longxing , Z. Yuyao , L. Wencong , Y. Lu , and H. Hu , “Easily Recyclable Photocatalyst Bi_2_WO_6_/MOF/PVDF Composite Film for Efficient Degradation of Aqueous Refractory Organic Pollutants Under Visible‐Light Irradiation,” Journal of Materials Science 54 (2019): 6238–6257.

[247] C. Chen , S. Wang , F. Han , X. Zhou , and B. Li , “Synergy of Rapid Adsorption and Photo‐Fenton‐Like Degradation in CoFe‐MOF/TiO_2_/PVDF Composite Membrane for Efficient Removal of Antibiotics From Water,” Separation and Purification Technology 333 (2024): 125942.

[248] B. Rabeie and N. M. Mahmoodi , “Green and Environmentally Friendly Architecture of Starch‐Based Ternary Magnetic Biocomposite (Starch/MIL100/CoFe_2_O_4_): Synthesis and Photocatalytic Degradation of Tetracycline and Dye,” International Journal of Biological Macromolecules 274 (2024): 133318. 10.1016/j.ijbiomac.2024.133318 38917917

[249] S. Sharafinia , A. Farrokhnia , E. G. Lemraski , and A. Rashidi , “Decoration of ZnFe_2_O_4_ and UiO‐66 Over g‐C_3_N_4_ as Magnetically Novel Reusable Visible Light Photocatalyst for Degradation of Rh–B,” Optical Materials 132 (2022): 112838.

[250] E. M. El‐Fawal , S. A. Younis , and T. Zaki , “Designing AgFeO_2_‐Graphene/Cu_2_(BTC)_3_ MOF Heterojunction Photocatalysts for Enhanced Treatment of Pharmaceutical Wastewater Under Sunlight,” Journal of Photochemistry and Photobiology A: Chemistry 401 (2020): 112746.

[251] J. An , J. Wang , Y. Li , et al., “Schottky Junctions Integrated With S‐Scheme Heterojunctions in Recyclable Loaded ZnIn_2_S_4_/UiO‐66/Calcined Graphite Felt Photocatalysts With Enhanced Carrier Separation for Photocatalytic Degradation,” Separation and Purification Technology 359 (2025): 130814.

[252] Q. Wei , S. Xiong , W. Li , et al., “Double Z‐Scheme System of α‐SnWO_4_/UiO‐66 (NH_2_)/g‐C_3_N_4_ Ternary Heterojunction With Enhanced Photocatalytic Performance for Ibuprofen Degradation and H_2_ Evolution,” Journal of Alloys and Compounds 885 (2021): 160984.

[253] K. Abbassian and M. Rahmani , “Triple Heterojunction Photocatalytic Composites Based on CQDs/TiO_2_@Ti‐TPA‐MOF/Cu_0. 5_Zn_0.5_S for Visible‐Light‐Degradation,” Journal of Water Process Engineering 61 (2024): 105308.

[254] Y. Bu , F. Li , Y. Zhang , R. Liu , X. Luo , and L. Xu , “Immobilizing CdS Nanoparticles and MoS_2_/RGO on Zr‐Based Metal–organic Framework 12‐Tungstosilicate@UiO‐67 Toward Enhanced Photocatalytic H_2_ Evolution,” RSC Advances 6 (2016): 40560–40566.

[255] J. Xu , J. Gao , C. Wang , Y. Yang , and L. Wang , “NH_2_‐MIL‐125 (Ti)/Graphitic Carbon Nitride Heterostructure Decorated With NiPd Co‐Catalysts for Efficient Photocatalytic Hydrogen Production,” Applied Catalysis B: Environmental 219 (2017): 101–108.

[256] Y. Zhang , Z. Jin , and G. Wang , “Charge Transfer Behaviors Over MOF‐5@g‐C_3_N_4_ With NixMo_1−x_S_2_ Modification,” International Journal of Hydrogen Energy 43 (2018): 9914–9923.

[257] Z. Song , S. Song , W. Zhang , et al., “The Unique Z‐Scheme g‐C_3_N_4_/Ti_3_C_2_/Sn‐Bi‐MOF Photocatalyst for Enhancing CO_2_ Reduction Activity,” Fuel 366 (2024): 131154.

[258] M. B. Nguyen and H. V. Doan , “Enhanced Oxidative Desulfurization of Dibenzothiophene Under Visible Light Using Carbon Quantum Dot‐Decorated Novel Z‐Scheme BiVO_4_/MOF‐808/CN Photocatalyst: Mechanism, Performance and Stability,” Journal of the Taiwan Institute of Chemical Engineers 164 (2024): 105691.

[259] J. Li , Y. Jing , S. Zhang , et al., “WO_3_@MOF Heterostructure In‐Situ Transformed and Deposited on La‐Bi_2_WO_6_‐BiFeWO_x_ Composite With Enhanced Photocatalytic Performance for Degradation of Harmful and Toxic Substances Produced by Tobacco,” Chemical Engineering Journal 499 (2024): 156508.

[260] S. Zhong , B. Kang , X. Cheng , P. Chen , and B. Fang , “Construction of a Co‐MOF/MXene/BiVO_4_ Composite Photoanode for Efficient Photoelectrochemical Water Splitting,” ACS Sustainable Chemistry & Engineering 12 (2024): 1233–1246.

[261] C. Li , G. Ding , X. Liu , et al., “Photocatalysis Over NH_2_‐UiO‐66/CoFe_2_O_4_/CdIn_2_S_4_ Double p‐n Junction: Significantly Promoting Photocatalytic Performance by Double Internal Electric Fields,” Chemical Engineering Journal 435 (2022): 134740.

[262] Y. Dou , S.‐M. Xu , A. Zhou , et al., “Hierarchically Structured Semiconductor@Noble‐Metal@MOF for High‐Performance Selective Photocatalytic CO_2_ Reduction,” Green Chemical Engineering 1 (2020): 48–55.

[263] V. Moradi , M. B. Jun , A. Blackburn , and R. A. Herring , “Significant Improvement in Visible Light Photocatalytic Activity of Fe Doped TiO_2_ Using an Acid Treatment Process,” Applied Surface Science 427 (2018): 791–799.

[264] A. Ali , M. Muslim , I. Neogi , M. Afzal , A. Alarifi , and M. Ahmad , “Construction of a 3D Metal–organic Framework and Its Composite for Water Remediation via Selective Adsorption and Photocatalytic Degradation of Hazardous Dye,” ACS Omega 7 (2022): 24438–24451. 10.1021/acsomega.2c01869 PMC9301640 35874213

[265] D. Zhao and C. Cai , “Adsorption and Photocatalytic Degradation of Pollutants on Ce‐Doped MIL‐101‐NH_2_/Ag_3_PO_4_ Composites,” Catalysis Communications 136 (2020): 105910.

[266] J. Lee and J. Kim , “Heterostructured Photocatalytic Fabric Composed of Ag_3_PO_4_ Nanoparticle‐Decorated NH_2_‐MIL‐88B(Co/Fe) Crystalline Wires for Rhodamine B Adsorption and Degradation,” ACS Applied Nano Materials 7 (2024): 8362–8375.

[267] Y. Cao , L. Yue , Z. Li , et al., “Construction of Sn‐Bi‐MOF/Ti_3_C_2_ Schottky Junction for Photocatalysis of Tetracycline: Performance and Degradation Mechanism,” Applied Surface Science 609 (2023): 155191.

[268] J. W. Maina , C. Pozo‐Gonzalo , L. Kong , J. Schütz , M. Hill , and L. F. Dumée , “Metal Organic Framework Based Catalysts for CO_2_ Conversion,” Materials Horizons 4 (2017): 345–361.

[269] L. Wang , P. Jin , S. Duan , H. She , J. Huang , and Q. Wang , “In‐Situ Incorporation of Copper(II) Porphyrin Functionalized Zirconium MOF and TiO_2_ for Efficient Photocatalytic CO_2_ Reduction,” Science Bulletin 64 (2019): 926–933. 10.1016/j.scib.2019.05.012 36659757

[270] L. Wang , P. Jin , J. Huang , H. She , and Q. Wang , “Integration of Copper(II)‐Porphyrin Zirconium Metal–organic Framework and Titanium Dioxide to Construct Z‐Scheme System for Highly Improved Photocatalytic CO_2_ Reduction,” ACS Sustainable Chemistry & Engineering 7 (2019): 15660–15670.

[271] W.‐Y. Gao , M. Chrzanowski , and S. Ma , “Metal–metalloporphyrin Frameworks: A Resurging Class of Functional Materials,” Chemical Society Reviews 43 (2014): 5841–5866. 10.1039/c4cs00001c 24676096

[272] Y.‐Z. Chen , Y.‐X. Zhou , H. Wang , et al., “Multifunctional PdAg@ MIL‐101 for One‐Pot Cascade Reactions: Combination of Host–guest Cooperation and Bimetallic Synergy in Catalysis,” ACS Catalysis 5 (2015): 2062–2069.

[273] C. Wang , S. Wang , Y. Ping , et al., “Ru@MIL‐125/MnO_x_ Metal‐Organic‐Framework‐Based Cocatalysts for Photocatalytic Nitrogen Fixation,” Applied Catalysis B: Environment and Energy 347 (2024): 123781.

[274] J. Di , B. Lin , B. Tang , S. Guo , J. Zhou , and Z. Liu , “Engineering Cocatalysts Onto Low‐Dimensional Photocatalysts for CO_2_ Reduction,” Small Structures 2 (2021): 2100046.

[275] A. Makdee , P. Kidkhunthod , Y. Poo‐arporn , and K. C. Chanapattharapol , “Enhanced CH_4_ Selectivity for CO_2_ Methanation Over Ni‐TiO_2_ by Addition of Zr Promoter,” Journal of Environmental Chemical Engineering 10 (2022): 107710.

[276] X. Zhu , H. Xu , C. Bi , et al., “Piezo‐Photocatalysis for Efficient Charge Separation to Promote CO_2_ Photoreduction in Nanoclusters,” Ultrasonics Sonochemistry 101 (2023): 106653. 10.1016/j.ultsonch.2023.106653 PMC10638044 37918293

[277] C. Hou , Y. Li , M. Niu , et al., “Construction of an All‐Solid‐State Z‐Scheme Ag@Ag_3_PO_4_/TiO_2_‐(F_2_) Heterostructure With Enhanced Photocatalytic Activity, Photocorrosion Resistance and Mechanism Insight,” Journal of Alloys and Compounds 925 (2022): 166765.

[278] S. Li , H. Li , Y. Wang , et al., “Mixed‐Valence Bimetallic Ce/Zr‐NH_2_‐UiO‐66 Modified With CdIn_2_S_4_ to Form S‐Scheme Heterojunction for Boosting Photocatalytic CO_2_ Reduction,” Separation and Purification Technology 333 (2024): 125994, 10.1016/j.seppur.2023.125994.

[279] C. Luo , M. Liao , D. Peng , J. Xiong , and H. Zeng , “Fe‐Doped MOF(Ti)/Bi_2_MoO_6_ Heterostructure for Boosting Oxidation/Reduction Activities Under Visible Light,” Colloids and Surfaces a: Physicochemical and Engineering Aspects 706 (2025): 135798, 10.1016/j.colsurfa.2024.135798.

[280] W. Lou , L. Wang , S. Dong , Z. cao , J. Sun , and Y. Zhang , “A Facility Synthesis of Bismuth‐Iron Bimetal MOF Composite Silver Vanadate Applied to Visible Light Photocatalysis,” Optical Materials 126 (2022): 112168, 10.1016/j.optmat.2022.112168.

[281] F. Guo , W. Xiao , C. Ma , et al., “Constructing Gas Transmission Pathways in Two‐Dimensional Composite Material ZIF‐8@ BNNS Mixed‐Matrix Membranes to Enhance CO_2_/N_2_ Separation Performance,” Membranes 13 (2023): 444. 10.3390/membranes13040444 PMC10143403 37103871

[282] K. Javed , N. Abbas , M. Bilal , et al., “Fabrication of a ZnFe_2_O_4_@Co/Ni‐MOF Nanocomposite and Photocatalytic Degradation Study of Azo Dyes,” RSC Advances 14 (2024): 30957–30970. 10.1039/d4ra05283h PMC11429226 39346520

[283] R. Hariri and S. Dehghanpour , “Adsorptive Removal and Visible‐Light Photocatalytic Degradation of Large Cationic and Anionic Dyes Induced by Air‐Bubbles in the Presence of a Magnetic Porphyrinic Metal‐Organic Framework (Fe_3_O_4_@SiO_2_@PCN‐222(Fe,” Journal of Physics and Chemistry of Solids 155 (2021): 110126, 10.1016/j.jpcs.2021.110126.

[284] L. Xia , X. Cheng , L. Jiang , et al., “High‐Performance Bismuth Vanadate Photoanodes Cocatalyzed With Nitrogen, Sulphur Co‐Doped Ferrocobalt‐Metal Organic Frameworks Thin Layer for Photoelectrochemical Water Splitting,” Journal of Colloid and Interface Science 659 (2024): 676–686, 10.1016/j.jcis.2024.01.049. 38211485

[285] Y. Sun , X. Wang , Q. Fu , and C. Pan , “A Novel Hollow Flower‐Like 0D/3D Zn_0.5_Cd_0.5_S/NiCoZn‐LDH Photocatalyst With n‐n Heterojunction for High Hydrogen Production,” Applied Surface Science 564 (2021): 150379, 10.1016/j.apsusc.2021.150379.

[286] B. Zhao , J. Liu , Y. Liu , W. Wen , and Z. Li , “In Situ Growth of ZnCdS Nanoparticles on Bimetallic Cu/Fe‐MOF for Efficient Visible‐Light‐Driven Photocatalytic Hydrogen Production and Urea Synthesis,” Journal of Alloys and Compounds 1011 (2025): 178395, 10.1016/j.jallcom.2024.178395.

[287] M. Cao , F. Yang , Q. Zhang , et al., “Facile Construction of Highly Efficient MOF‐Based Pd@UiO‐66‐NH_2_@ZnIn_2_S_4_ Flower‐Like Nanocomposites for Visible‐Light‐Driven Photocatalytic Hydrogen Production,” Journal of Materials Science & Technology 76 (2021): 189–199, 10.1016/j.jmst.2020.11.028.

[288] S. Ma , X. Wang , K. Wan , B. Liu , Y. Yang , and S. Wang , “Metal‐Organic Framework‐Derived Zn_x_Cd_1‐x_S/Zn_x_Cd_1‐x_‐MOF Heterostructures Promoting Charge Separation for Photocatalytic Hydrogen Evolution,” Separation and Purification Technology 354 (2025): 129089, 10.1016/j.seppur.2024.129089.

[289] S. Wang , G. Hu , Y. Dou , et al., “Z‐Scheme Promoted Interfacial Charge Transfer on Cu/in‐Porphyrin MOFs/CdIn_2_S_4_ Heterostructure for Efficient Photocatalytic H_2_ Evolution,” Separation and Purification Technology 354 (2025): 129220, 10.1016/j.seppur.2024.129220.

[290] B. Antil , L. Kumar , M. R. Das , and S. Deka , “Incorporating NiCoP Cocatalyst Into Hollow Rings of ZnCo‐Metal–Organic Frameworks to Deliver Pt Cocatalyst Like Visible Light Driven Hydrogen Evolution Activity,” ACS Applied Energy Materials 5 (2022): 11113–11121, 10.1021/acsaem.2c01716.

[291] H. Ramezanalizadeh and F. Manteghi , “Immobilization of Mixed Cobalt/Nickel Metal‐Organic Framework on a Magnetic BiFeO_3_: A Highly Efficient Separable Photocatalyst for Degradation of Water Pollutions,” Journal of Photochemistry and Photobiology A: Chemistry 346 (2017): 89–104, 10.1016/j.jphotochem.2017.05.041.

[292] Y. Li , Q. Wang , X. Hu , et al., “Constructing NiFe‐Metal‐Organic Frameworks From NiFe‐Layered Double Hydroxide as a Highly Efficient Cocatalyst for BiVO_4_ Photoanode PEC Water Splitting,” Chemical Engineering Journal 433 (2022): 133592, 10.1016/j.cej.2021.133592.

[293] H.‐Z. Liu , X. Liu , B. Li , H. Luo , J.‐G. Ma , and P. Cheng , “Hybrid Metal–Organic Frameworks Encapsulated Hybrid Ni‐Doped CdS Nanoparticles for Visible‐Light‐Driven CO_2_ Reduction,” ACS Applied Materials & Interfaces 14 (2022): 28123–28132, 10.1021/acsami.2c08776. 35679596

[294] M. B. Nguyen , L. H. T. Nguyen , M. T. Le , et al., “Engineering Direct Z‐Scheme GCN/ bimetallic‐MOF Heterojunctions as Efficient and Recyclable Photocatalysts for Enhancing Degradation of RR. 195 Under Visible Light,” Journal of Industrial and Engineering Chemistry 134 (2024): 217–230, 10.1016/j.jiec.2023.12.052.

[295] E. Halvacı , M. Kaya , Y. E. Serin , O. Ozdemir , A. Aygun , and F. Sen , “Highly Efficient Hydrogen Production and Biochemical Studies With CNT Supported Pd@Ce‐MOF Nanocomposite Structure,” International Journal of Hydrogen Energy 112 (2025): 144–152, 10.1016/j.ijhydene.2025.02.337.

[296] K. Divyarani , S. Sreenivasa , T. M. C. Rao , et al., “Boosting Sulfate Radical Assisted Photocatalytic Advanced Oxidative Degradation of Tetracycline via Few‐Layered CoZn@MOF/GO Nanosheets,” Colloids and Surfaces A: Physicochemical and Engineering Aspects 671 (2023): 131606, 10.1016/j.colsurfa.2023.131606.

[297] K. Divyarani , S. Sreenivasa , V. S. Anusuaya devi , et al., “An Efficient Novel NiCu@INA/rGO MOF Hollow Microsphere Z‐Scheme Heterojunction Catalyst for the Photocatalytic Degradation of Tetracycline and Simultaneous Degradation of Cationic and Anionic Dyes,” Colloids and Surfaces A: Physicochemical and Engineering Aspects 703 (2024): 135415, 10.1016/j.colsurfa.2024.135415.

[298] H. Jin , W. Zhou , Z. Guo , et al., “Consolidating CQDs With NiFe‐MOF Photo‐Fenton Membranes to Effectively Treat Wastewater Containing Antibiotics and Antibiotic‐Resistant Bacteria,” Chemical Engineering Journal 498 (2024): 155740, 10.1016/j.cej.2024.155740.

[299] P. Lv , F. Duan , J. Sheng , et al., “The 2D/2D p–n Heterojunction of ZnCoMOF/g‐C_3_N_4_ With Enhanced Photocatalytic Hydrogen Evolution Under Visible Light Irradiation,” Applied Organometallic Chemistry 35 (2021): e6124, 10.1002/aoc.6124.

[300] W. Wang , S. Song , P. Wang , et al., “Chemical Bonding of g‐C_3_N_4_/UiO‐66(Zr/Ce) From Zr and Ce Single Atoms for Efficient Photocatalytic Reduction of CO_2_ Under Visible Light,” ACS Catalysis 13 (2023): 4597–4610, 10.1021/acscatal.2c06255.

[301] E. Gontarek‐Castro , A. Pancielejko , M. A. Baluk , et al., “Photocatalytic Membranes Based on Cu–NH_2_‐MIL‐125(Ti) Protected by Poly(vinylidene Fluoride) for High and Stable Hydrogen Production,” Materials Horizons 12 (2025): 1504–1515, 10.1039/D4MH01397B. 39714344

[302] L. Ding , M. Lei , T. Wang , J. Wang , and Z. Jin , “Graphdiyne Coordinated CoMo‐MOF Formed S‐Scheme Heterojunction Boosting Photocatalytic Hydrogen Production,” Carbon Letters 34 (2024): 2099–2112.

[303] K. Boukayouht , N. U. M. Nor , Y. Ait‐Khouia , W. L. Queen , N. A. Saidina Amin , and S. El Hankari , “Enhanced Photocatalytic CO_2_ Reduction to Methanol Over Eco‐Friendly CuCo‐ZIF@g‐C_3_N_4_ Synthesized From Recyclable Resources,” Inorganic Chemistry 64 (2025): 4308–4319, 10.1021/acs.inorgchem.4c04838. 39992627

[304] C. Huang , G. Lu , N. Qin , et al., “Enhancement of Output Performance of Triboelectric Nanogenerator by Switchable Stimuli in Metal–Organic Frameworks for Photocatalysis,” ACS Applied Materials & Interfaces 14 (2022): 16424–16434, 10.1021/acsami.2c01251. 35377137

[305] Q. Wen , D. Li , H. Li , et al., “Synergetic Effect of Photocatalysis and Peroxymonosulfate Activated by Co/Mn‐MOF‐74@g‐C_3_N_4_ Z‐Scheme Photocatalyst for Removal of Tetracycline Hydrochloride,” Separation and Purification Technology 313 (2023): 123518, 10.1016/j.seppur.2023.123518.

[306] K. Wang , X. Kong , H. Xie , S. Li , M. Wang , and Z. Jin , “High Performance of Visible‐Light Driven Hydrogen Production Over Graphdiyne (g‐C_n_H_2n−2_)/MOF S‐Scheme Heterojunction,” Dalton Transactions 52 (2023): 8716–8727, 10.1039/D3DT01344H. 37310365

[307] S. Ren , J. Dong , X. Duan , et al., “A Novel (Zr/Ce)UiO‐66(NH_2_)@g‐C_3_N_4_ Z‐Scheme Heterojunction for Boosted Tetracycline Photodegradation via Effective Electron Transfer,” Chemical Engineering Journal 460 (2023): 141884, 10.1016/j.cej.2023.141884.

